# Effect of liposome rigidity on corona formation and its implications for uptake by cells

**DOI:** 10.1038/s41467-026-77231-7

**Published:** 2026-09-01

**Authors:** Xinyu Ma, Feng Zhao, Miguel Cantero-Reviejo, Sander de Weerd, Ilaria Squillante, Giuseppe Portale, Wouter H. Roos, Anna Salvati

**Affiliations:** 1 Nanomedicine and Drug Targeting, Groningen Research Institute of Pharmacy, University of Groningen, Groningen, the Netherlands; 2 Molecular Biophysics, Zernike Institute for Advanced Materials, University of Groningen, Groningen, the Netherlands; 3 Macromolecular Chemistry & New Polymeric Materials, Zernike Institute for Advanced Materials, University of Groningen, Groningen, the Netherlands

**Keywords:** Drug delivery, Endocytosis, Nanobiotechnology

## Abstract

Nanoparticle rigidity influences cellular uptake and in vivo behavior, yet the underlying mechanisms remain unclear. Here we used lipids with identical head groups but different acyl chains to prepare liposomes with comparable surface composition, size and zeta potential, but distinct mechanical properties. Rigid liposomes showed much higher uptake than soft ones, mediated by distinct endocytic mechanisms. Proteomic analysis revealed that rigid and soft liposomes adsorbed very different coronas upon dispersion in serum. RNA interference and competition studies indicated that the enhanced uptake of rigid liposomes is likely driven by the lower abundance of multiple apolipoproteins in their corona, and possibly the higher abundance of apolipoprotein H and of a pattern of low-abundant corona proteins. Importantly, a strong difference in uptake was observed only when liposomes were added to cells in the presence of serum. Hence, the different uptake efficiency is not solely due to the different mechanical properties, but results from the effect of liposome mechanical properties on corona formation.

## Abstract (web summary)

Nanoparticle rigidity affects their cell uptake and in vivo behaviour, but the mechanisms remain unclear. Here, the authors show that rigid and soft liposomes form different coronas in serum and only when the corona is present does the uptake differ.

## Introduction

Nanomedicine uses nanomaterials of different physico-chemical properties, such as size and surface charge, to deliver drugs to their target^1,2^. Nanoparticle mechanical properties, particularly rigidity, have recently drawn significant attention, as they affect in vivo distribution, circulation time, and cellular uptake efficiency^3–5^. Theoretical studies show that rigidity affects membrane bending and wrapping during internalization by cells. Rigid nanoparticles can enforce membrane wrapping more easily, whereas softer ones deform along the membrane before inducing bending, requiring higher energy for internalization^3,5,6^. Many studies reported enhanced phagocytic uptake of rigid nanoparticles in vivo, leading to faster clearance and shorter circulation time^4,7,8^. Instead, results regarding the effect of rigidity on the uptake by cancer cells remain inconsistent and often appear contradictory^4,9–11^. This is likely due to the fact that many factors that can affect uptake efficacy are varied across different studies, such as the explored range of nanoparticle rigidity, nanoparticle material, size, surface charge, and incubation conditions. Overall, a clear fundamental understanding of how nanoparticle rigidity affects nanoparticle interactions at the cell and organism level is still lacking.

Within this context, one aspect that remains largely unexplored is whether corona formation is affected by nanoparticle mechanical properties. Once exposed to biological environments, nanoparticles are known to rapidly adsorb a corona of proteins and other biomolecules, which critically shapes their biological identity^12^. The corona can promote interactions with specific cell receptors and potentially endow nanoparticles with endogenous targeting capabilities, as reported for Onpattro, the first RNA therapeutic, as well as the so-called SORT (selective organ targeting) lipid nanoparticles and several other systems^13–18^. While nanomaterial properties such as size and surface charge are known to modulate corona composition^12,19,20^, surprisingly little is known on whether nanoparticle rigidity affects it as well^21,22^.

To address this question, here we selected liposomes as a model to obtain nanoparticles of different mechanical properties but comparable surface composition, size, and zeta potential. Modulating nanoparticle mechanical properties without affecting other physico-chemical parameters is very challenging. Different models have been developed and used for this purpose, each presenting some advantages and some limitations^11,23–25^. Here, liposomes composed of lipids with different lipid chain saturation were selected as a model system because they allow to modulate nanoparticle mechanical properties without affecting some of the other key physico-chemical properties known to affect nanoparticle biological behavior. In fact, depending on their composition and transition temperature, lipid bilayers can form different phases: solid ordered phase (S_o_) bilayers are rigid, while liquid disordered (L_d_) phase bilayers are soft^26,27^. By selecting lipid mixtures with different lipid chain length and saturation, liposome transition temperature can be modulated^24,28^. Hence, by varying lipid chain saturation and bilayer phase, liposomes of very different mechanical properties can be formed, while keeping their size, charge, and surface composition comparable.

Of note, liposomes with saturated lipids can often agglomerate and are usually stabilized by addition of pegylated lipids. However, inclusion of pegylated lipids reduces corona formation, hence interfering with the aim of this study. Therefore, extensive optimization was performed by tuning liposome charge and lipid mixture composition in order to vary the transition temperature of the bilayers, and obtain two non-pegylated liposomes of distinct mechanical properties that were stable in biological fluids. Next, atomic force microscopy (AFM) was used to quantify nanoparticle deformation under varying imaging forces, providing a direct comparison of their relative rigidity. The cellular uptake of the two liposomes was determined, and their uptake mechanisms were characterized. Mass spectrometry (MS) was used to compare the composition of the coronas formed on the two liposomes. RNA interference, competition experiments, and chemical inhibitors were combined in order to determine whether differences in corona composition could lead to the involvement of different receptors in the uptake of the two formulations. Finally, liposomes were dispersed in biological media of different composition in order to determine the effect of the resulting coronas on uptake efficiency. In this way, we disentangled how nanoparticle mechanical properties affect corona formation and their consequences on uptake by cells.

## Results and discussion

### Optimization and physico-chemical characterization of liposome formulations

To obtain liposomes of same size, zeta potential and surface composition but different rigidity, lipids with identical head groups but varied chain lengths and saturation were selected. Liposomes of ~100 nm were prepared by thin layer hydration and extrusion. Initially, pure zwitterionic liposomes were prepared, with same surface properties but different phase transition temperature (Supplementary Fig. 1)^29^. Uptake was higher for the liposomes with saturated lipids, however, they were not stable in the biological medium used for cell studies (MEM supplemented with 10% v/v fetal bovine serum, hereafter referred to as complete medium, cMEM, Supplementary Fig. 1).

Liposomes agglomeration in biological medium was observed also for formulations where saturated lipids were mixed with increasing amounts of unsaturated lipids in order to lower the transition temperature, T_m_, below 37 °C (Supplementary Fig. 1B). Next, we incorporated negatively charged phosphatidylglycerol (PG) lipids with identical acyl chains to increase the particle charge for improved stability, but without altering the T_m_. Stability in cMEM was achieved only when incorporating high amount of PG lipids (30–50%, see Supplementary Fig. 2 for details). However, possibly because of the strongly negative charge, uptake of the saturated and unsaturated charged liposomes was comparable (Supplementary Fig. 2).

**Fig. 1:**
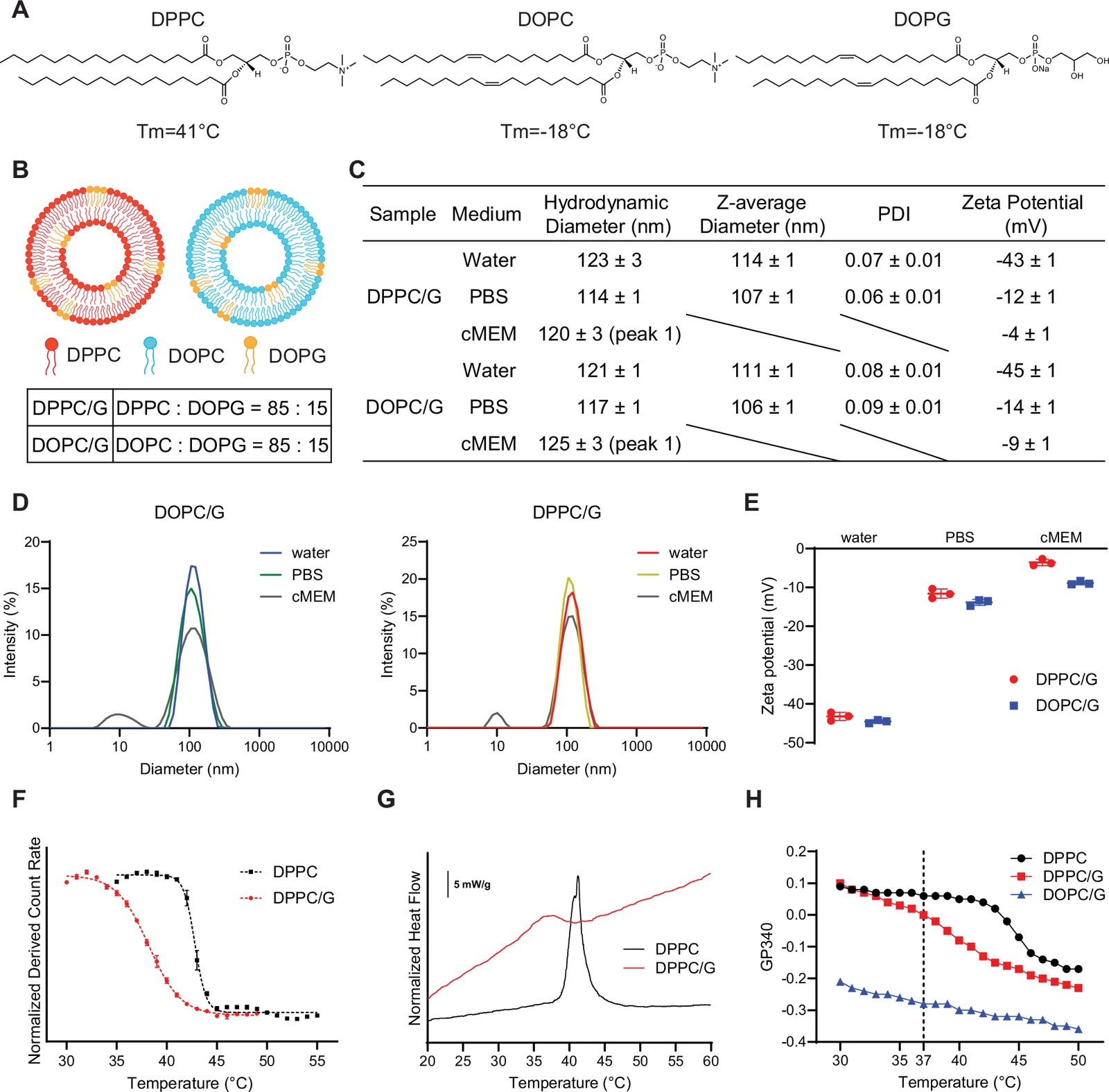
Formulation and characterization of the optimized liposomes. **A** Chemical structure of DPPC, DOPC, and DOPG lipids included in DPPC/G and DOPC/G liposomes, together with their structural formulas and transition temperature (T_m_). **B** Schematic illustration and composition of the two optimized liposome formulations. **C** Hydrodynamic diameter, Z-average diameter, and polydispersity index (PDI) obtained by DLS and zeta potential measurements of liposome dispersions in different media (100 µg/mL liposomes in water, PBS, and cMEM). The hydrodynamic diameter (nm) was obtained by analyzing the DLS data with the General-Purpose Algorithm, and the corresponding size distributions by intensity are shown in panel (**D**). The Z-average diameter and PDI were obtained by cumulant analysis of the data. The results are the average and SD of the results obtained for three different batches of liposomes (*n* = 3). **E** Comparison of the zeta potential of the two liposomes upon dispersion in different media. The results obtained for three different batches of liposomes are shown (*n* = 3), together with their average, indicated by a line, and SD. For each batch, 3 repeated measurements were performed, and the mean value is shown as one data point. **F** Normalized derived count rate changes of 1 mM DPPC and DPPC/G liposome in PBS at increasing temperature as obtained by DLS. The mean and standard deviation of the results obtained for 3 repeated measurements of the same sample in one representative experiment are shown (*n* = 1). **G** Normalized Differential Scanning Calorimetry (DSC) traces of 10 mg/mL DPPC and DPPC/G liposomes in PBS. **H** Laurdan Generalized Polarization ratio (GP_340_) values at increasing temperature for DPPC, DPPC/G, and DOPC/G liposomes (0.4 mg/mL in water). Laurdan was incorporated in liposomes at 1% molar ratio. The GP_340_ values are the average of the results obtained for 3 replicate samples in one representative experiment (*n* = 1), calculated from the fluorescence intensity of laurdan at 440 nm and 490 nm as described in the Methods. Source data are provided as a Source Data file.

Based on these results, we modulated both T_m_ and charge by mixing DPPC or DOPC with unsaturated negatively charged DOPG at the minimum ratio necessary to obtain liposomes that remained stable in biological conditions and still showed different uptake efficiency. This led to a final formulation of DOPC/G and DPPC/G liposomes with an 85:15 PC:PG ratio (Fig. 1A, B). The two liposomes had comparable size, (~110 nm in water and PBS, as measured by DLS and Nanoparticle Tracking Analysis), and were stable over time (Fig. 1C, D & Supplementary Fig. 3A-B). Their zeta potential was also comparable (around −43 mV in H_2_O and −12 mV in PBS, Fig. 1C, E). Importantly, the optimized liposomes were stable upon dispersion in medium with serum, where a second small peak at around 10 nm was also visible from the free serum proteins in solution (Fig. 1D and Supplementary Fig. 3B for results up to 24 h at 37 °C and 5% CO_2_, hence in the conditions used for cell experiments). Interestingly, the zeta potential in medium with serum further decreased towards neutrality as expected upon corona formation, but was slightly different for the two liposomes (−3.6 ± 0.8 mV for DPPC/G and −8.9 ± 0.5 mV for DOPC/G), suggesting potential differences in corona composition (Fig. 1E).

In order to further characterize the optimized liposomes, DLS was employed to determine their transition temperature (T_m_)^30^ by monitoring the count rate (the number of photons detected per second) of the liposome dispersions at increasing temperature (Fig. 1F and Supplementary Fig. 3C for raw data). A discontinuity in the count rate can be observed when there is a change in the optical properties of the liposome, and this indicates a phase transition^30^. The corresponding temperature T_m_ can be calculated by fitting the curve with a Boltzmann model (see Eq. 1 in the “Methods”). The results showed that DPPC/G liposomes exhibited a broader transition with a T_m_ of 38.2 °C, slightly lower than that of pure DPPC liposomes (42.8 °C). In line with this, Differential Scanning Calorimeter (DSC) showed that pure DPPC liposomes exhibited a main transition peak at 41.2 °C. Instead, a much broader peak at around 37.3 °C was observed for DPPC/G liposomes (Fig. 1G and raw data in Supplementary Fig. 3D). Overall, these results confirmed that the T_m_ of DPPC/G liposomes is slightly lower than that of pure DPPC liposomes, as expected due to the addition of 15% DOPG.

We next compared the fluidity of the two formulations upon incorporation of laurdan in the bilayer^31^. Changes in laurdan fluorescence emission are commonly used to characterize membrane fluidity and this is often used as an indicator of membrane rigidity and of the different mechanical properties of liposomes with bilayers in different phases^26^. In fact, when laurdan is incorporated in bilayers in the ordered phase (S_o_, L_o_), it shows maximum emission at around 440 nm, while when inserted in bilayers in the liquid disordered phase (L_d_) the maximum emission is at around 490 nm. Based on this, a Generalized Polarization ratio (GP_340_) can be calculated (as shown in Eq. 2 in the “Methods”). It is important to keep in mind that as a consequence of the different bilayer phase both the mechanical properties, as well as the hydration level of the bilayer are affected. In fact the laurdan GP ratio is also used as an indicator of the bilayer hydration at the liposome surface^32,33^. In the gel phase, tight lipid packing excludes water from the interface, while in the liquid disordered phase, looser packing permits greater water penetration. Therefore, in this model, nanoparticle mechanical properties and the bilayer hydration level are both varied, because they are strongly intertwined and cannot be separated, since they are both consequences of the different bilayer phase.

The results obtained with laurdan fluorescence showed that in line with literature^31,34^, pure DPPC liposomes at 30 °C displayed a high GP_340_ value of 0.1, consistent with laurdan inclusion into a solid ordered phase. The GP_340_ value dropped to below -0.1 (liquid disordered phase) when the temperature increased above 41 °C, consistent with the T_m_ of pure DPPC (Fig. 1H). DOPC/G liposomes instead showed GP_340_ values lower than –0.2 at all temperatures tested, as expected for unsaturated lipids in the liquid disordered phase. In contrast, for DPPC/G liposomes the GP_340_ value decreased gradually at increasing temperature. Watanabe previously reported that laurdan in multiple hydration status shows a more gradual GP_340_ decrease, and suggested this is due to membrane region heterogeneity and/or incomplete phase segregation^35^. It would be interesting to determine whether inclusion of a small fraction of DOPG lipids in the liquid phase into the solid DPPC bilayer indeed leads to the formation of clusters of lipids and phase segregation. Consistent with these results, at 37 °C (as used for cell studies) the GP_340_ value for DPPC/G liposomes was slightly lower than that for DPPC, but considerably higher than for DOPC/G liposomes. Overall, these results confirmed that DPPC/G and DOPC/G liposomes exhibit distinct bilayer fluidity, influenced by their respective T_m_ values.

**Fig. 2:**
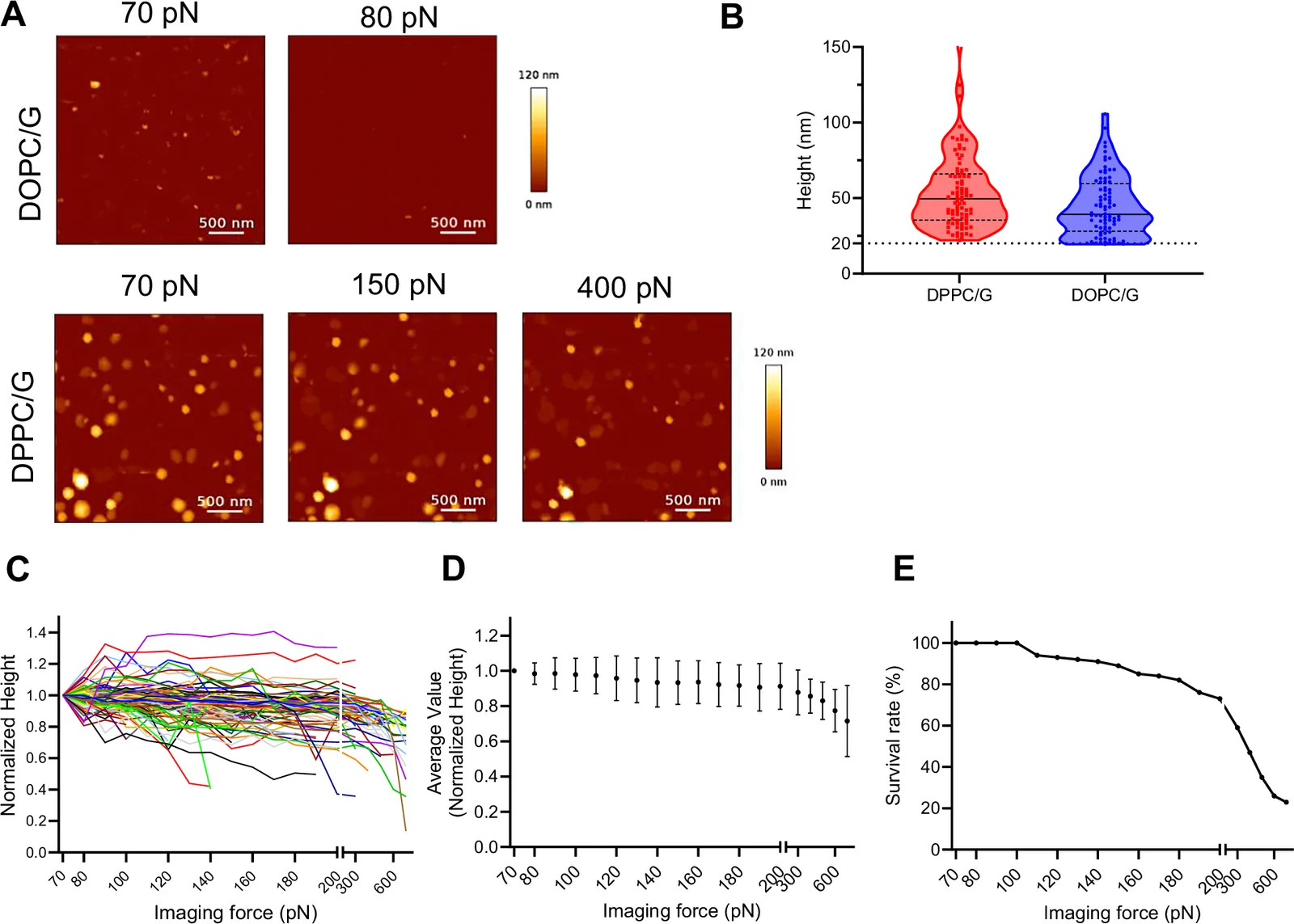
Characterization of liposome mechanical properties by AFM. **A** Topographic images of DOPC/G and DPPC/G liposomes under increasing imaging forces. Most DOPC/G liposomes collapsed at 80 pN while the majority of DPPC/G liposomes still remained intact up until 400 pN. Scale bar: 500 nm. **B** Height of individual liposomes measured at an imaging force of 70 pN. A total of 88 DPPC/G liposomes and 84 DOPC/G liposomes were analysed (*n* = 88 and 84, respectively). The black line indicates the median and the dashed black lines indicate the interquartile range. **C**,** D** Normalized height of DPPC/G liposomes as a function of the imaging force. Panel** C** shows the results obtained for 88 individual particles while **D** shows the mean ± SD of the same results (*n* = 88 individual particles). **E** Survival rate of DPPC/G liposomes at increasing imaging forces from 70 to 700 pN. Normalized height and survival rate data were calculated for liposomes that (i) did not collapse on the surface (height over 20 mm) and (ii) resisted at least three consecutive imaging forces of 70, 80, and 90 pN. Source data are provided as a Source Data file.

Next, atomic force microscopy (AFM) was employed to compare the mechanical properties of the two liposomes. Representative topographic images are shown in Fig. 2A (images at all imaging forces are shown in Supplementary Fig. 4A). By imaging individual liposomes at increasing imaging forces, the decrease in height can be directly measured and provides a comparison of the difference in mechanical properties between samples. At low imaging force (70 pN), the heights of the DPPC/G and DOPC/G liposomes were generally lower than the diameters detected by DLS (Fig. 1D), as typically observed because of liposome deformation upon surface attachment^36^. Notably, even at such low imaging force, DOPC/G liposomes showed a lower height, indicating higher deformability, as well as a greater number of lipid patches (Fig. 2A, B). The presence of these patches further indicated a higher deformability and consequently a higher rate of collapse upon adsorption onto the glass. At increased forces only a few liposomes remained intact. Conversely, DPPC/G liposomes demonstrated greater resilience (see all images in Supplementary Fig. 4A). We next tracked the height change of individual DPPC/G liposomes that withstood at least three consecutive imaging forces (Fig. 2C, D and Supplementary Fig. 4B for raw data) and their survival rates (Fig. 2E). Even after using forces up to 200 pN, 73% of the DPPC/G liposomes remained intact. Analysis of the height vs force at 200 pN indicates a reduction of 10% of the original height measured at 70 pN. Gradually increasing the force further, up to 700 pN, resulted in total collapse of most DPPC/G liposomes, with an estimated survival population of 23%. These results clearly demonstrated the distinct mechanical properties of the two liposome formulations, with DOPC/G liposomes being much softer and brittle than DPPC/G liposomes. To further compare liposome mechanical properties, an analysis of indentation curves was performed. Due to—among others—pressure built up of the adhered vesicles and fluidity of bilayers, it is challenging to obtain elastic parameters^36,37^. Supplementary Fig. S5 shows a relative comparison of the elasticity, denoted as the apparent modulus *M*_*app*_, of the two liposomes. The results confirmed that the *M*_*app*_ of the DPPC/G liposomes is higher than that of the DOPC/G liposomes, consistent with results of increased imaging force experiments. Based on the two methods for the quantitative characterization of nanoparticle mechanical properties by AFM, we refer hereafter to the two liposomes as “soft” and “rigid” liposomes, for simplicity.

### Cellular uptake behavior

**Fig. 3:**
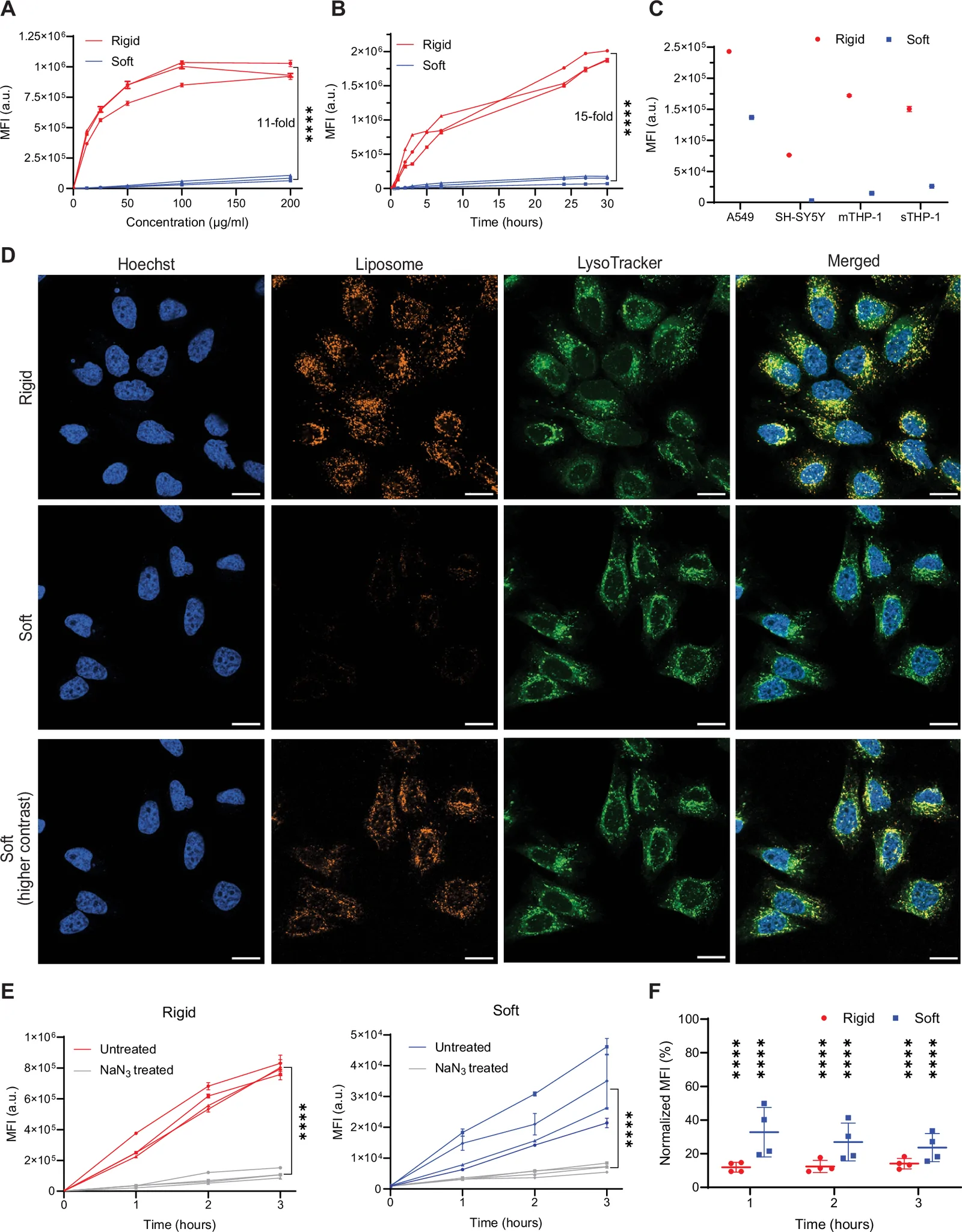
Effect of liposome rigidity on uptake by cells. **A** Uptake of soft and rigid liposomes at different concentrations in HeLa cells after 5 h incubation in cMEM**. B** Uptake kinetics in HeLa cells upon incubation with 50 µg/mL rigid or soft liposomes in cMEM for up to 30 h. In panels **A**,** B**, the results obtained in 3 individual experiments by flow cytometry are shown (*n* = 3). Each symbol represents the mean ± SD of the median cell fluorescence intensity (MFI) obtained from 3 replicate samples. **C** Uptake of soft and rigid liposomes (100 µg/mL) in A549, SH-SY5Y, mTHP-1, sTHP-1 cells after 5 h incubation in the corresponding medium supplemented with 10% FBS. Data are presented as mean ± SD of the MFI of 3 replicate samples in 3 independent experiments (*n* = 3). **D** Confocal fluorescence images of HeLa cells after incubation with 100 µg/mL rigid or soft liposomes in cMEM for 5 h. Higher contrast images for soft liposomes are included to confirm liposome internalization. Blue: Hoechst-stained nuclei. Red: liposomes. Green: LysoTracker Green DND-26-stained lysosomes. Scale bar: 20 µm. **E** Uptake kinetics of 100 µg/mL rigid and soft liposomes in cMEM in standard conditions (Untreated) or in presence of 5 mg/mL sodium azide (NaN_3_ treated) to deplete cell energy. The results obtained in 4 independent experiments are shown (*n* = 4), and each data point is the mean ± SD of the MFI from 3 replicate samples. In **(F)** the results of panel **(E**) are shown after normalization for the uptake in untreated control cells at each time point. The normalized values from 4 independent experiments (*n* = 4) are presented, together with their mean ± SD. An unpaired two-sided one-way analysis of variance (ANOVA) with Dunnett’s correction for multiple comparisons was used for statistical analysis for panel (**F**), and an unpaired two-sided Welch’s t-test was used for the other panels (*p* values are reported when *p* < 0.05, which was considered significant. ****p < 0.0001). Source data are provided as a Source Data file.

Having confirmed the different mechanical properties of the optimized liposomes, we compared their cellular uptake behavior on HeLa cells. Results at different liposome concentrations and over time showed that uptake of the rigid liposomes was much higher than for the soft ones (Fig. 3A, B, up to 15-fold higher at 30 h incubation). Similar outcomes were observed in a range of cell lines, including A549 epithelial cells, SH-SY5Y neuroblastoma cells, THP-1 monocyte (mTHP-1) and PMA-stimulated THP-1 (sTHP-1) as a common model for human macrophages (Fig. 3C), suggesting the higher uptake of rigid liposomes may be a general behavior across different cell types.

Confocal fluorescence microscopy (Fig. 3D) confirmed liposome internalization and the overall higher uptake for the rigid liposomes, consistent with the results obtained by flow cytometry. LysoTracker staining showed that both liposomes were trafficked to the lysosomes, as usual for most nanomaterials. Other studies suggested that some liposomes may enter cells by passive fusion to the cell membrane^9^. Here instead, energy depletion by incubation with the metabolic inhibitor sodium azide (NaN_3_) confirmed that for the liposomes used in this work uptake was energy-dependent rather than by passive fusion (Fig. 3E, F). The strong drop of cell fluorescence in energy depleted cells also indicated that liposome adhesion to the cell membrane was minimal. Consistent with this, no membrane associated staining was observed by confocal microscopy in cells incubated with the liposomes in standard conditions (Supplementary Fig. 7). This was also a confirmation that the fluorescent lipid DiI used for liposome tracing remained associated within the liposomes inside endocytic compartments. Similarly, no membrane-associated staining was observed in energy-depleted cells either (Supplementary Fig. 7). These results confirmed that the lower fluorescence of cells incubated with the soft liposomes was not due to effects of DiI dilution in fused cell membranes on its fluorescence emission, but rather due to a lower uptake efficiency.

**Fig. 4:**
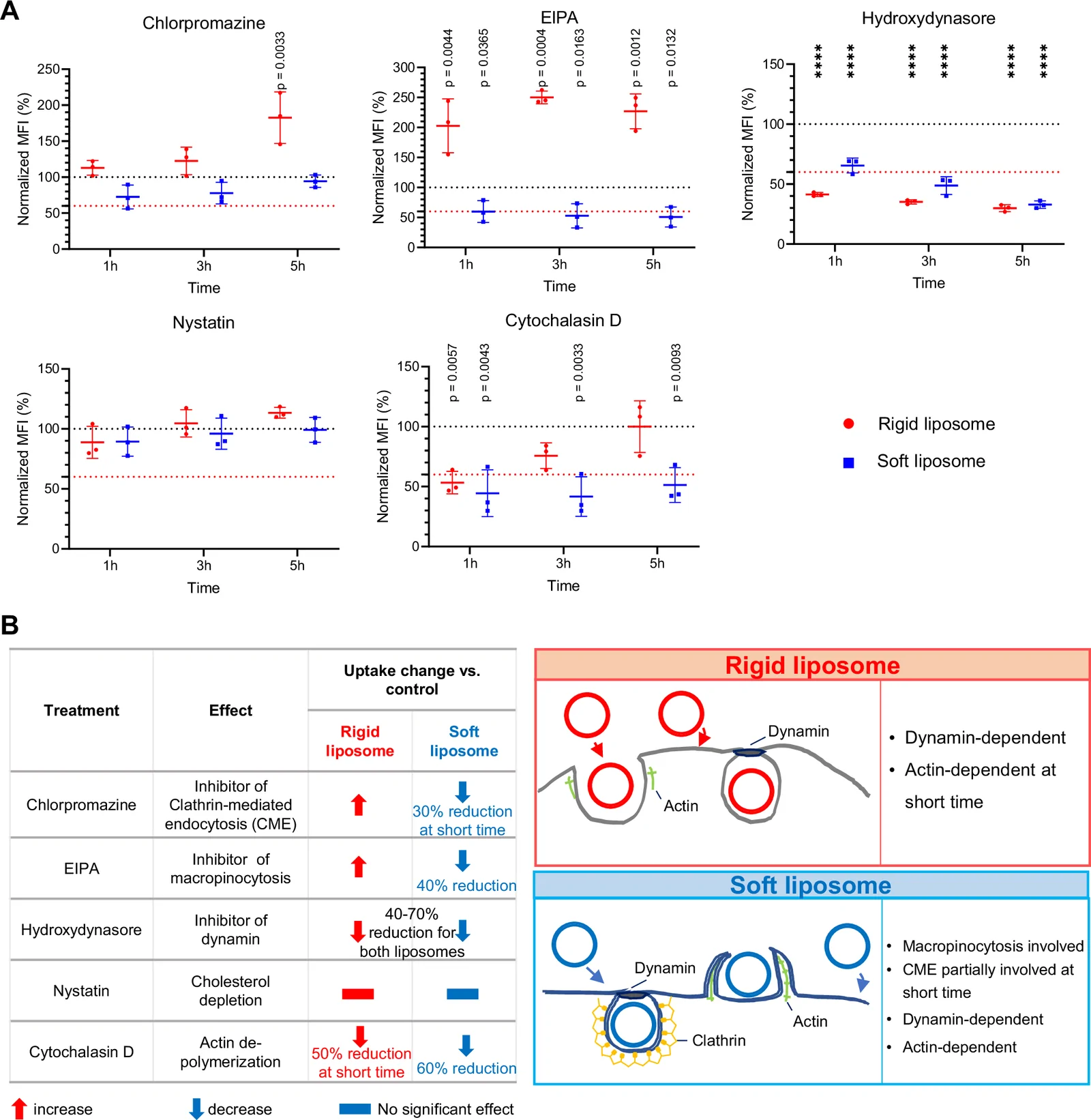
Characterization of liposome uptake mechanisms. **A** Normalized fluorescence intensity of HeLa cells incubated with soft or rigid liposomes (100 µg/mL) in cMEM in the presence of inhibitors (10 µg/mL chlorpromazine; 50 µM EIPA; 10 mg/mL hydroxydynasore; 25 µg/mL nystatin or 2.5 µg/mL cytochalasin D) compared to standard conditions. The results obtained in three independent experiments are shown together with their mean ± SD (*n* = 3). A black dashed line and a red dashed line are drawn in each panel as a reference of 100% and 60% uptake respectively, as an indicative threshold for inhibition efficacy. An unpaired two-sided one-way analysis of variance (ANOVA) with Dunnett’s correction for multiple comparisons was used for normalized data (*p* values are reported when p < 0.05, which was considered significant. ****p < 0.0001). **B** Summary of transport inhibitor effects and schematic illustration of potential uptake mechanisms involved in the uptake of soft and rigid liposomes. Source data are provided as a Source Data file.

As a next step, a panel of common transport inhibitors was used to determine whether the different uptake efficiency observed for the soft and rigid liposomes could be explained by the involvement of different uptake mechanisms. Importantly, additional DLS studies were performed to confirm that the liposome dispersions in medium with serum remained stable also upon addition of the different inhibitors (Supplementary Fig. 8). Agglomeration in the presence of the inhibitors could strongly affect uptake studies and impair their interpretation. The results showed that inhibition of clathrin-mediated endocytosis (CME) with chlorpromazine strongly increased uptake of the rigid liposomes, potentially due to activation of compensatory mechanisms, while for the soft liposomes it reduced uptake of around 30–40% in the first hours (Fig. 4 and Supplementary Fig. S8 for raw data). Similarly, blocking macropinocytosis with EIPA increased uptake of the rigid liposomes, while it reduced uptake of the soft liposomes up to 40%. Incubation with the dynamin inhibitor hydroxydynasore and with nystatin to deplete cholesterol from the cell membrane indicated that for both liposomes endocytosis was dynamin dependent, but not cholesterol dependent, respectively. Finally, blocking actin polymerization with cytochalasin D reduced the uptake of soft liposomes up to ~60–70%, while for the rigid liposomes effects were visible only at shorter incubation times. Overall, the observed reduction of uptake upon incubation with different transport inhibitors confirmed once more that the liposomes were internalized by cells via active mechanisms of endocytosis (as opposed to passive fusion to the cell membrane). More specifically, the results suggested that for both liposomes uptake was actin- and dynamin-dependent, but cells likely internalize the two liposomes via different mechanisms, with macropinocytosis and (at short time) clathrin-mediated endocytosis involved in the uptake of the soft liposomes (Fig. 4B provides a schematic summary of these results).

### Isolation and characterization of the corona composition on soft and rigid liposomes

**Fig. 5:**
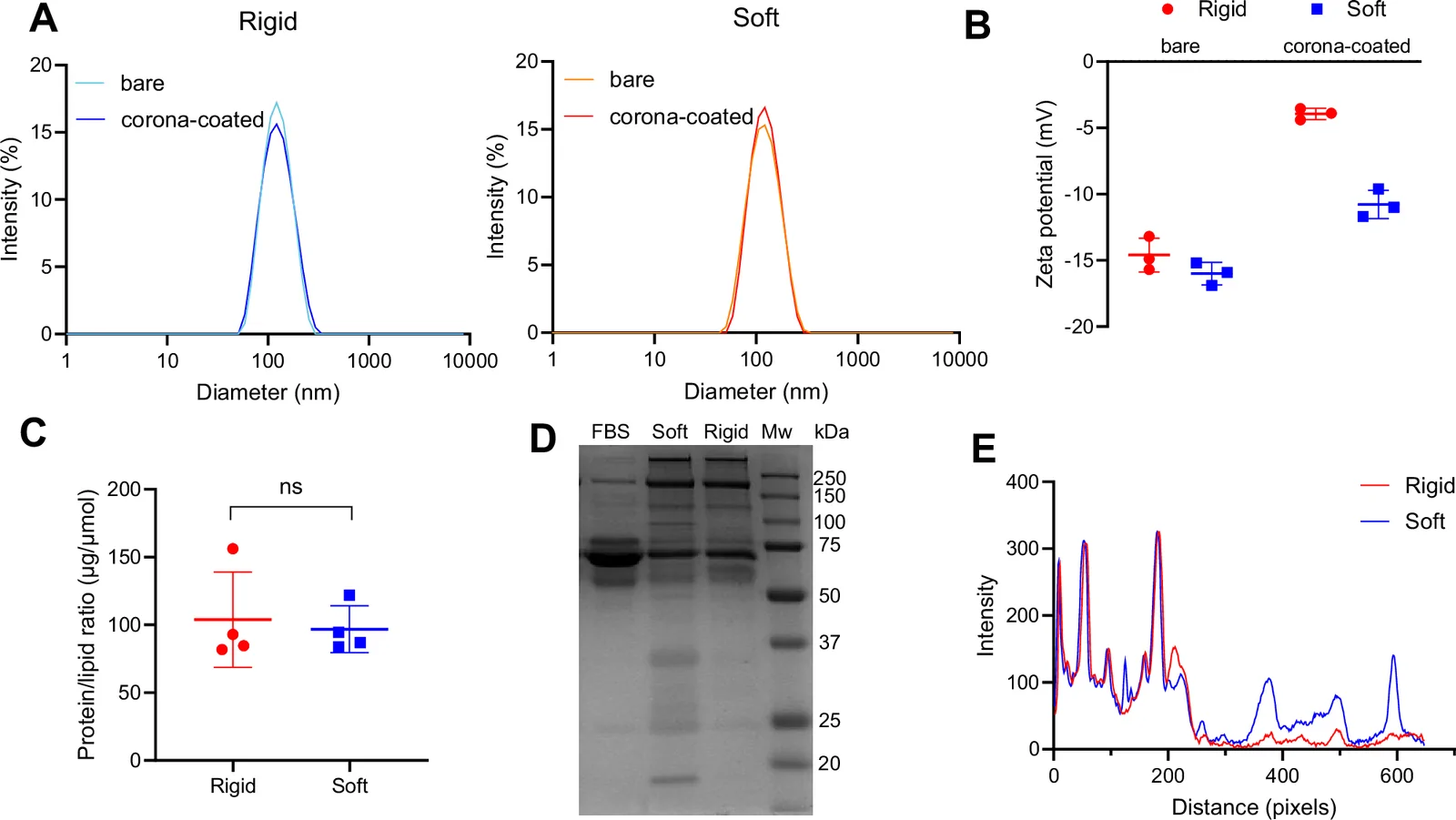
Characterization of corona-coated liposomes. Size distribution by intensity (**A**) and zeta potential (**B**) of 100 µg/mL liposomes (bare) and the corresponding isolated corona-coated liposomes (corona-coated) in PBS. In panel **B**, the results obtained for 3 replicate measurements of 1 sample in a representative experiment are shown (*n* = 1), together with their mean ± SD. **C** Comparison of the amount of proteins absorbed on rigid or soft liposomes after corona isolation. The protein binding capacity (PBC) was defined as the amount of absorbed proteins (μg) per micromole of lipids. The results obtained from 4 independent corona isolation experiments are shown together with their mean ± SD (*n* = 4). **D** SDS-PAGE image of corona proteins adsorbed on rigid or soft liposomes. The same amount of lipids was loaded in each lane (0.15 µmol), together with 15 μg FBS as control (FBS). Corona isolation and SDS-PAGE were repeated for a total of 4 independent experiments with similar results. The result of one representative experiment is shown (the same results are shown also in Supplementary Fig. 9B where additional lanes from the same gel are included as well for further comparisons). **E** Intensity profiles of the bands of corona proteins in the SDS-PAGE image shown in panel (**D**). An unpaired two-sided Welch’s *t* test was used for statistical analysis (*p* values are reported when *p* < 0.05, which was considered significant. ns: *p* > 0.05). Source data are provided as a Source Data file.

To correlate the differences in uptake behavior with potential differences in the protein corona formed upon dispersion in serum, corona-coated liposomes were isolated using size exclusion chromatography (SEC)^38^. During SEC, nanoparticles with their strongly associated proteins (the so-called hard corona) elute first through the column, while the loosely associated proteins (soft corona) and the excess free proteins elute later. Hence, SEC is a common method to separate nanoparticles with their hard corona from the loosely bound soft corona proteins and the free serum proteins in solution^12,38^. FBS samples eluted by SEC were also tested to exclude contamination by particles of size and density comparable to liposomes in FBS (e.g., protein aggregates, lipid nanoparticles, exosomes, etc., Supplementary Fig. 9A)^39^. The isolated hard corona-coated liposomes were well dispersed (Fig. 5A), and—interestingly—displayed slightly different zeta potential (Fig. 5B), suggesting differences in their corona composition. Protein quantification indicated that comparable amount of proteins were absorbed on both rigid and soft liposomes (Fig. 5C). Next, SDS-PAGE was used to separate the isolated corona proteins (Fig. 5D and additional results in Supplementary Fig. S9B). Interestingly, despite having the same size, surface properties and similar amount of adsorbed proteins, the two liposomes adsorbed very different corona proteins, particularly in the low molecular weight range below 50 kDa, where multiple bands with stronger intensity were detected for the soft liposomes (Fig. 5D, E). These results clearly indicated that the two liposomes adsorbed different protein coronas upon dispersion in serum. The different coronas likely resulted from the observed differences in nanoparticle mechanical properties (Fig. 2 and Supplementary Fig. 5), as well as potential additional contributions due to differences in bilayer fluidity and bilayer hydration levels, that in this model are strongly intertwined.

**Fig. 6:**
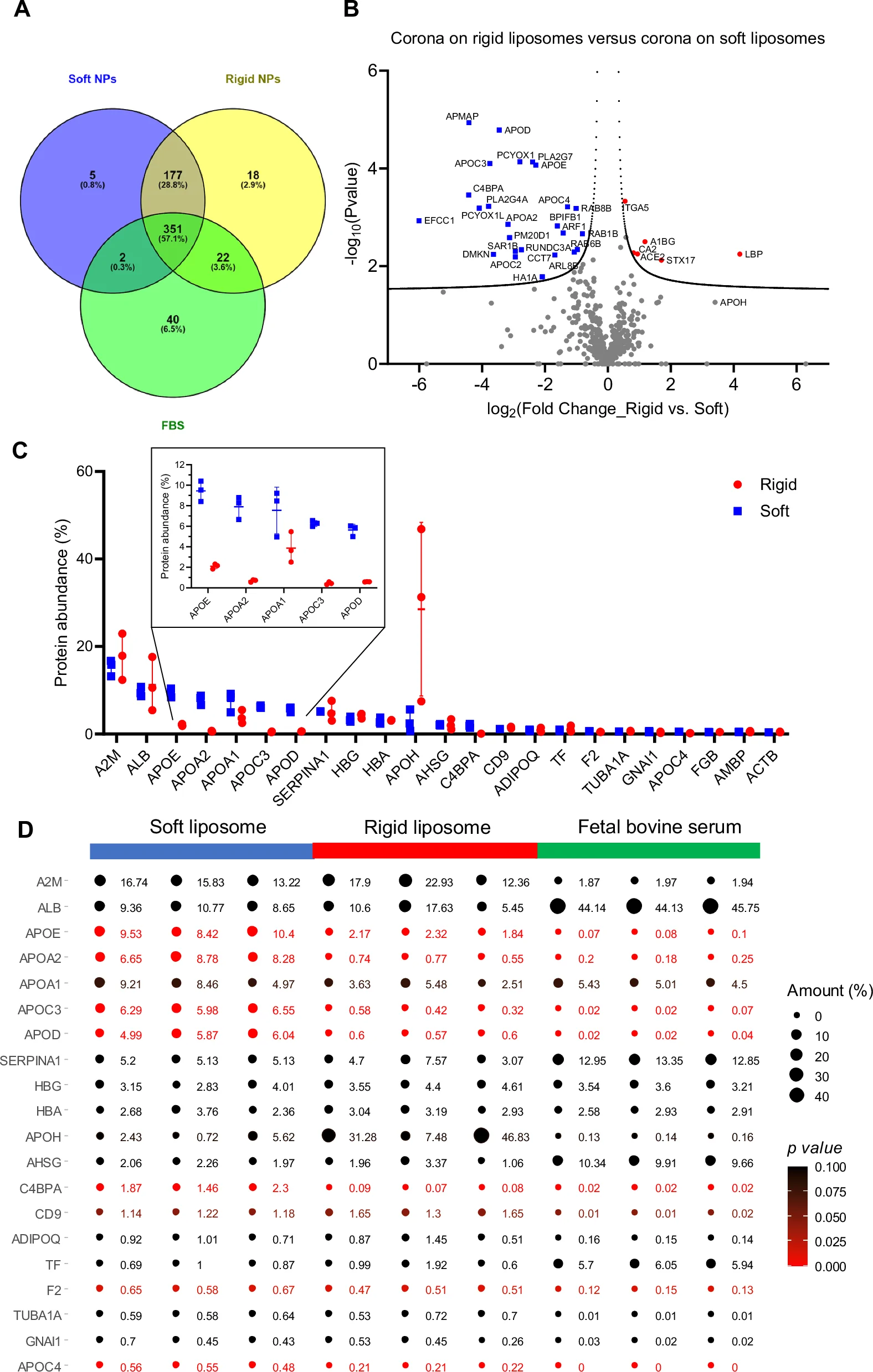
Analysis of the protein corona on rigid and soft liposomes. **A** Venn diagram of the corona proteins identified on soft or rigid liposomes and in FBS. **B** Volcano plot of differentially enriched corona proteins on rigid liposomes in respect to soft liposomes. The differential protein abundance was analyzed in Perseus using a two-sided *t* test with permutation-based false discovery rate correction. The black lines indicate the significance thresholds (FDR = 0.05, s0 = 0.1). Red dots and blue squares represent highly enriched and depleted corona proteins, respectively, identified on rigid liposomes in respect to soft liposomes. Six proteins were significantly enriched on the rigid liposomes, whereas twenty-five proteins were significantly depleted. All enriched and depleted proteins are shown in the plot and their specific abundances are listed in Supplementary Table 1. **C** Abundance of top 20 most abundant proteins in the corona formed on rigid or soft liposomes. In the insert, a zoomed-in graph highlights highly abundant apolipoproteins in the two coronas. The results obtained from 3 independent corona isolations and proteomic analyses are shown, together with their mean, indicated by a line, and SD. **D** Analysis of the top 20 most abundant proteins in the corona formed on soft liposomes. For each liposome, the mass spectrometry results obtained for 3 replicate corona isolations are reported, together with results for FBS in 3 replicate samples, for comparison. The relative abundance of the top 20 most abundant proteins identified in the corona formed on soft liposomes is indicated, together with their relative abundance in the rigid liposome corona and in FBS. The balloon area shows the protein abundance and the colors from black to red correspond to the *p* values of the one-way ANOVA analysis between soft and rigid samples (*n* = 3). Source data are provided as a Source Data file.

Next, label-free liquid chromatography tandem mass spectrometry (LC-MS/MS) was used to identify the isolated proteins and compare the composition of the hard corona formed on rigid and soft liposomes. Consistent with the SDS-PAGE results, Pearson correlation and Principal Component Analysis (PCA) confirmed strong differences in protein composition between the coronas formed on the two liposomes as well as in respect to FBS (Supplementary Fig. 10B-C), while replicate samples within each group showed relatively high reproducibility (Venn diagram in Fig. 6A and Supplementary Fig. 10A. The complete analysis of the proteomic results is also included as Supplementary Data).

When comparing the different samples, the majority of identified proteins were shared by both rigid and soft liposomes. Near 200 proteins were identified only in the corona samples but not in FBS, where they fell below detection limits, likely due to their low abundance. This is consistent with what is usually reported upon corona formation, where typically a very strong enrichment of low abundant proteins is observed. The top 20 most abundant proteins accounted for around 85% of the total protein corona abundance in both rigid and soft liposomes. While these proteins were present in both coronas, they were generally more enriched in the soft liposomes (Fig. 6C, D, and Supplementary Fig. 10D). This confirmed that nanoparticle rigidity leads to selective enrichment of specific corona proteins on the surface of the two liposomes.

Among 528 overlapping proteins detected in both coronas, 25 proteins were significantly depleted in the corona formed on the rigid liposomes in respect to the soft liposomes, and 6 proteins were significantly enriched. However, the enriched proteins had very low abundance (individual log_2_(LFQ intensity) lower than 20, and combined abundance of only 0.26% of total recovered proteins) (Fig. 6B & Supplementary Table 1). Instead, 11 of the 25 significantly depleted proteins in the rigid corona were relatively highly abundant on the soft liposomes (log_2_(LFQ intensity) higher than 20), and 7 of them ranked within the top 21 abundant proteins in the soft liposome corona (Fig. 6C, and Supplementary Table 1). Given the lower uptake of the soft liposomes in respect to the rigid liposomes, it is likely that these include proteins with a stealth effect and “don’t eat me” signals, as reported for proteins such as clusterin, CD47 or histidine-rich glycoprotein (HRG)^40–42^. Among the most abundant proteins in the soft liposome corona, 6 were apolipoproteins, including APOA2, APOC3 and APOE, that collectively accounted for 33% of the total protein abundance in the soft liposome corona, compared to only 4% in rigid liposomes. Apolipoproteins are known to be commonly enriched in the corona forming on many different types of nanomaterials^12,14,15,43^. Conversely, the most abundant proteins in rigid liposomes were largely comparable to those in the soft liposomes, expect for apolipoprotein H (APOH, also known as beta-2-glycoprotein 1). Despite a strong variability among replicates, APOH was highly enriched in the rigid liposome corona, making up—on average—28.5% of the total proteins (rank 1), compared to only 2.9% in soft liposomes (rank 11). Its abundance in the rigid liposome corona was ~190-fold higher than in FBS (0.15%), being the most enriched protein in the corona formed on the rigid liposomes (Fig. 6C, Supplementary Fig. 10D). When instead looking at proteins that were uniquely detected in either rigid or soft liposomes (Supplementary Table 2, 3), 40 proteins were exclusive to soft liposomes and 7 to rigid liposomes. However, their overall abundance was minimal, contributing <0.3% and <0.1% in the rigid and soft liposome corona, respectively.

Overall, proteomic analysis confirmed that the two liposomes adsorbed very different coronas. The top 20 most abundant corona proteins were common, although most of them were more abundant on the soft liposomes, except for APOH, which was the most abundant protein in the rigid liposome corona. Instead, the unique proteins distinguishing the two coronas included a set of low-abundance proteins, contributing—for both liposomes—to only less than 0.3% of the corona.

### Corona receptors and effect of liposome corona on cell uptake

**Fig. 7:**
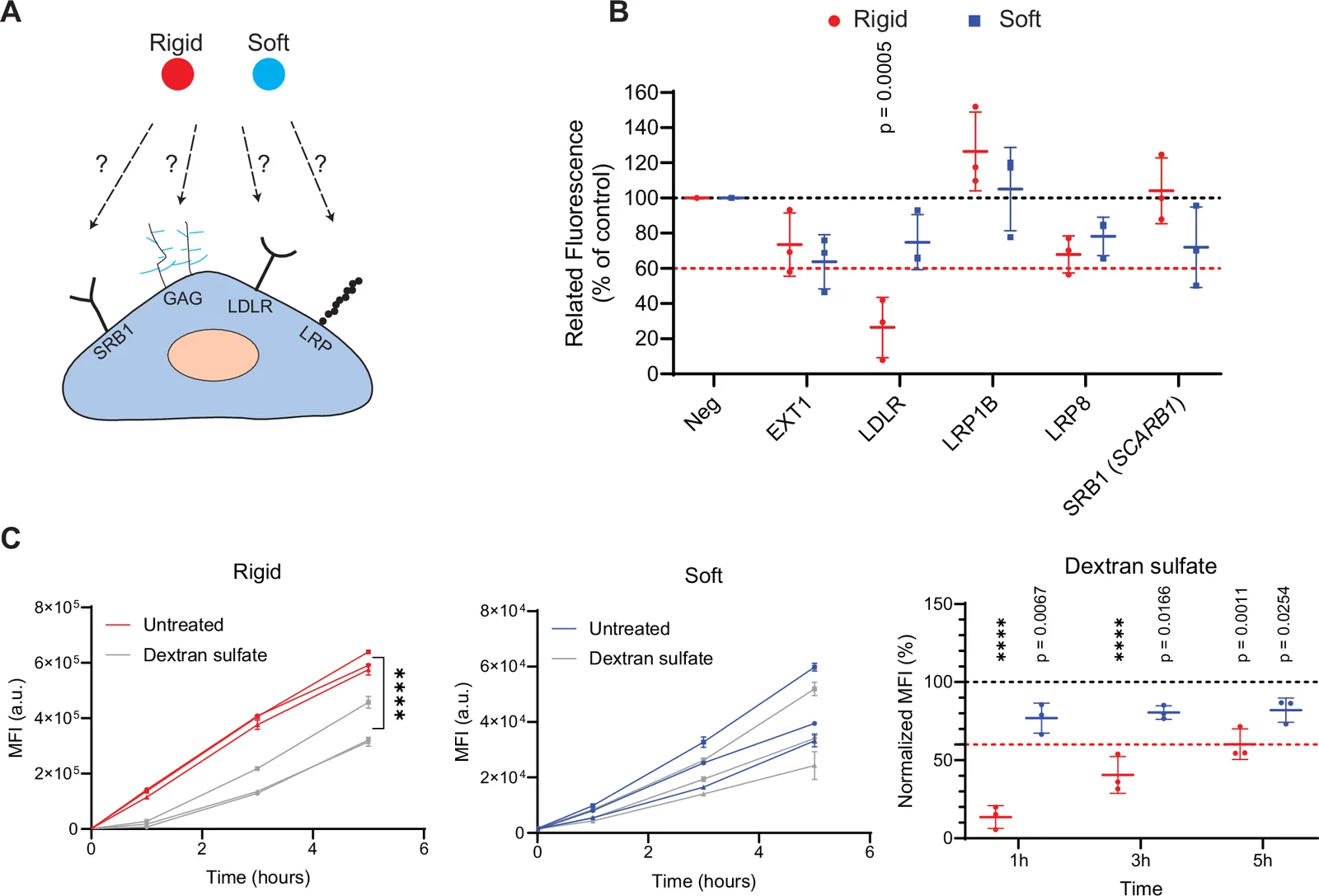
Characterization of receptors involved in liposome uptake. **A** Schematic illustration of a cell incubated with soft or rigid liposomes to investigate the role of SRB1, glycosaminoglycans (GAG), LDLR and LRP receptors in their internalization. **B** Uptake of 100 µg/mL rigid and soft liposomes in cMEM by HeLa cells (5 h incubation), after silencing the expression of known corona receptors. Data are presented as the mean ± SD of the median cell fluorescence intensity (MFI) obtained in 3 independent experiments (*n* = 3, each with 3 replicate samples), after normalization for the uptake in untreated control cells silenced with a scrambled siRNA (Neg). **C** Raw and normalized uptake kinetics of 100 µg/mL rigid and soft liposomes in HeLa cells in the presence of 10 µg/mL dextran sulfate. The data are the mean ± SD of the results obtained for 2 or 3 replicate samples in 3 individual experiments (*n* = 3). On the right, the same data are shown after normalization for the uptake in untreated cells. The normalized values of each experiment are shown, together with their mean, indicated by a line, and SD for 3 independent experiments (*n* = 3). Here and in panel (**B**), a black dashed line and a red dashed line are drawn as a reference of 100% and 60% uptake respectively, as an indicative threshold for inhibition efficacy. An unpaired two-sided one-way analysis of variance (ANOVA) with Dunnett’s correction for multiple comparisons was used for statistical analysis for panel (**B**) and normalized figure in panel (**C**), and an unpaired two-sided Welch’s *t* test was used for raw data in panel (**C**) (*p* values are reported when *p* < 0.05, which was considered significant. ****p < 0.0001). Source data are provided as a Source Data file.

Given the selective enrichment of several apolipoproteins in the corona formed on the two liposomes, as a next step we investigated whether some apolipoprotein receptors were involved in the uptake of soft and rigid liposomes. These included the low-density lipoprotein receptor LDLR and the scavenger receptor class B member 1, SRB1, that were already identified as corona receptors for different nanomaterials^11,13,18,44,45^. In addition, we tested whether known APOH receptors were involved in liposome uptake, especially for the rigid liposomes. APOH has been reported to bind to heparin^45^, a structure analog of heparan sulfate, which is one of the most common glycosaminoglycans (GAGs) on the cell surface^18^. GAGs, especially heparan sulfate, are frequently reported as co-receptors together with lipid particle receptors and are emerging as nanoparticle receptors in multiple studies, including for exosomes^18,46–48^. To investigate the role of heparan sulfate in the uptake of the two liposomes, exostosin glycosyltransferase 1 (*EXT1*), a gene involved in glycosaminoglycan metabolism, was also silenced to deplete heparan sulfate on the cell membrane^18^. *LRP1B* and *LRP8* were also included, since APOH is known to bind receptors of the LDL receptor-related protein (LRP) family (Fig. 7A, B)^49^. When silencing *EXT1* and *LRP8*, around 30–40% uptake reduction was observed for both liposomes, while no reduction was observed for *LRP1B*. Silencing *SCARB1* expression, which encodes SRB1, slightly reduced the uptake of soft liposomes (~30% reduction). Instead, silencing *LDLR* reduced uptake of both liposomes, but with much stronger effects for the rigid liposomes (~80% reduction versus 30%). Competition with dextran sulfate (Fig. 7C), commonly used to test the role of heparan sulfate in uptake, reduced the uptake of rigid liposomes up to 80% in the first hour, with decreasing effects over time (up to 40% reduction after 5 h), while instead it had only minor effects on the uptake of soft liposomes. Of note, DLS analysis showed that for the rigid liposomes incubation with dextran sulfate induced agglomeration (Supplementary Fig. 11A), hence complicating interpretation of these results.

Given the different response upon *LDLR* silencing for the two liposomes, we further investigated its role via competition experiments with LDL (Supplementary Fig. 11B). When incubating cells with liposomes in the presence of increasing amounts of LDL, for the soft liposomes a small uptake reduction was observed, consistent with the results obtained after *LDLR* silencing. In contrast, addition of LDL increased uptake of the rigid liposomes. Similar results were obtained when changing the design and experimental conditions used for the competition study (Supplementary Fig. 11C). DLS results confirmed that both liposomes remained stable after incubation with LDL (Supplementary Fig. 11D), thus excluding confounding effects on nanoparticle stability.

To assess whether the enrichment of APOH in the rigid liposome corona could explain their higher uptake, we compared the uptake of liposomes pre-coated with either BSA or APOH and then added to cells in cMEM or in serum free MEM. In serum free medium, uptake of the two liposomes pre-coated with BSA or APOH was comparable and overall much lower than what was observed for the rigid liposomes in cMEM (Supplementary Fig. 11E), suggesting that APOH alone cannot explain the higher uptake observed for rigid liposomes. Instead, in cMEM uptake was higher for the rigid liposomes regardless the pre-coating. Furthermore, when adding increasing concentrations of APOH to block its receptors (Supplementary Fig. 11F), the uptake of corona-coated liposomes in serum free medium was not reduced, while in cMEM it was reduced to comparable extent for both liposomes (30–40% reduction, Supplementary Fig. 11G).

Overall, no clear differences were observed in the role of the tested receptors in the uptake of the two liposomes, except for the stronger effect of *LDLR* silencing on the uptake of the rigid ones. Similarly, it was not possible to confirm whether enrichment of APOH in corona and uptake via APOH receptors were responsible for the higher uptake of the rigid liposomes. Indeed, competition experiments are challenging when testing families of receptors which are clearly connected with each other, as the lipoprotein receptor family^18^. For the same reason, silencing their expression can induce upregulation of other receptors in the same family, making it difficult to disentangle their roles^50^. Additionally, competition experiments for nanoparticles can be complicated by the potential binding of the competitor to the nanoparticle corona, further complicating interpretation.

**Fig. 8:**
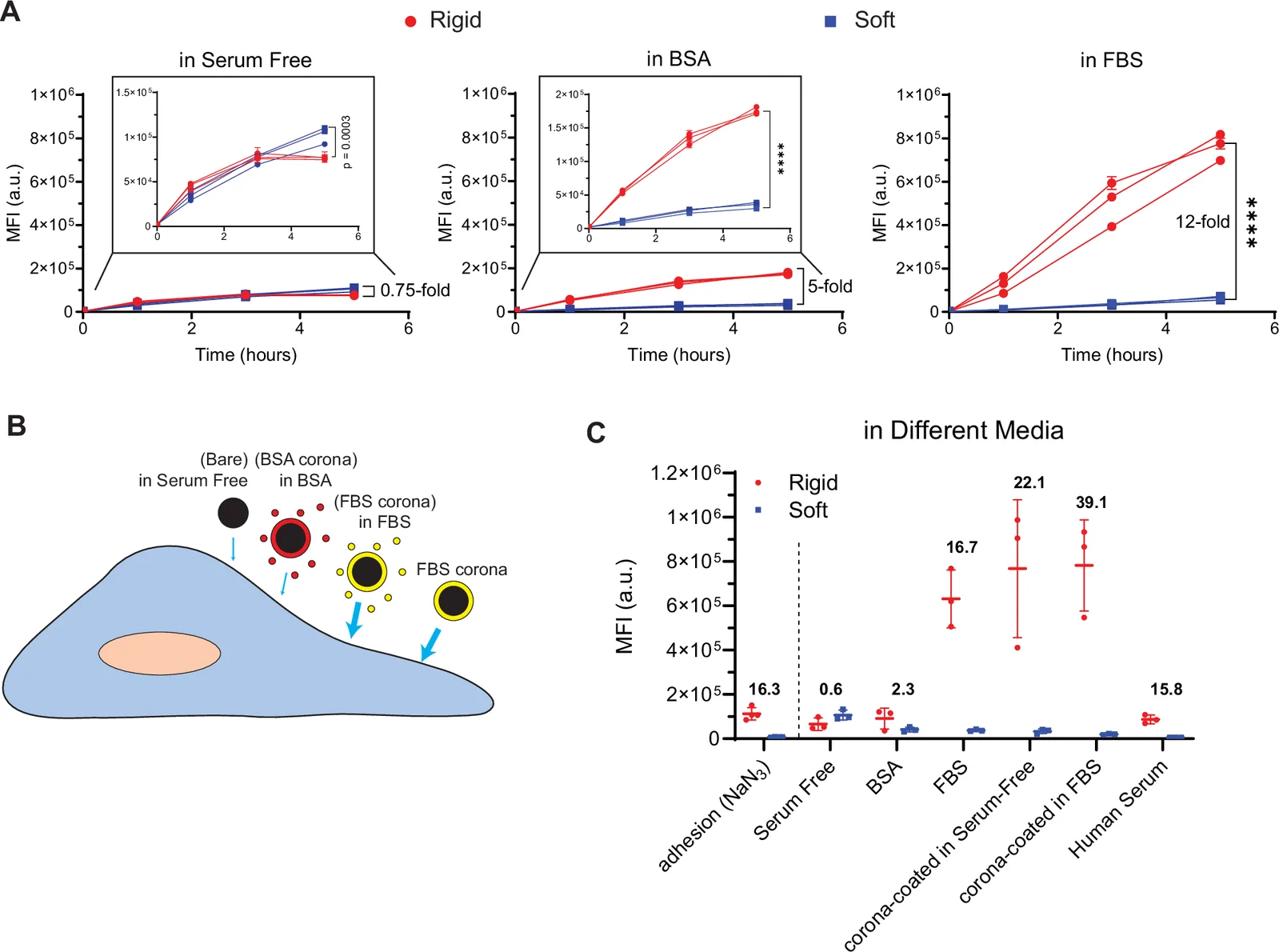
Liposome uptake behavior in different media. **A** Uptake kinetics of 100 µg/mL rigid and soft liposomes in serum free medium, 4 mg/mL BSA or 4 mg/mL FBS (roughly corresponding to standard cell culture medium with 10% FBS). The insets show enlarged views of the results obtained in serum free medium and with BSA. The results are the mean ± SD of the MFI obtained for two replicate samples in 3 independent experiments (*n* = 3). The fold difference in the uptake of rigid liposomes in respect to soft liposomes at 5 h is indicated. **B** Schematic illustration of the uptake experiments performed for liposomes in different media. The thickness of the blue arrows illustrates the relative uptake differences. **C** Uptake of liposomes added to cells in different conditions. Liposomes were incubated with cells for 5 h in serum free medium, or in medium containing 4 mg/mL BSA, FBS, or human serum. In addition, corona-coated liposomes were isolated and added to cells in either serum free medium or in medium with 4 mg/mL FBS (in all cases, liposomes and corona-coated liposomes were added to cells at 100 µg/mL lipid). Data from cells incubated with liposomes for 5 h in the presence of NaN_3_ to block active uptake are reproduced from Fig. 3E and included for reference as a measurement of liposome adhesion to cells. The results are the mean ± SD of the MFI obtained for two replicate samples in 3 independent experiments (*n* = 3). The fold difference in uptake between the two liposomes is indicated above each set of data points. An unpaired two-sided Welch’s *t* test was used for panel (**A**) (*p* values are reported when *p* < 0.05, which was considered significant. ****p < 0.0001). Source data are provided as a Source Data file.

As a further step to understand whether the differences in the corona adsorbed on the two liposomes could explain their different uptake efficiency, we compared the uptake of liposomes added to cells in different media. When liposomes were added to cells in serum free medium, thus in the absence of a serum corona on their surface, their uptake was comparable and generally rather low (Fig. 8A). This demonstrates that the mechanical properties alone cannot explain the higher uptake observed for rigid liposomes. When added to cells in a solution of BSA, uptake levels remained rather low, but the rigid liposomes had roughly 5 times higher uptake than the soft ones. Instead, when the liposomes were added to cells in the presence of FBS, where a full corona can form on their surface, uptake levels increased substantially and in these conditions uptake of the rigid liposomes was around 12 times higher than for the soft liposomes (Fig. 8A). Consistent with this, in cells incubated with the liposomes in energy depleted conditions, a comparable difference was observed on liposome adhesion to the cell membrane (around 16 time higher for the rigid liposomes). This suggests that the corona formed in serum promotes stronger adhesion to the cell membrane and this is likely to lead to the higher uptake observed. Similar uptake differences were observed for the two liposomes added to cells in human serum, although overall uptake efficiency was much lower, as typically observed for particles added to cells in human serum^51^. Finally, when hard corona-coated liposomes were first isolated by SEC and then added to cells in either serum free medium or reintroduced back in a medium with FBS, the uptake difference further increased to around 22 and 39-fold respectively (Fig. 8C). These results suggest that while soft corona proteins (which are lost upon corona isolation by SEC) and the excess free FBS proteins in solution also affect uptake, it is primarily the nature of the adsorbed hard corona layer formed in FBS which drives the higher uptake observed for the rigid liposomes.

Overall, these results clearly demonstrate that the different coronas formed on the soft and rigid liposomes in serum lead to the observed difference in uptake by cells, because a different uptake is observed only when liposomes are added to cells in medium with serum or for corona-coated liposomes, while no such difference is present in the absence of corona for liposomes added in serum free medium to cells nor in simple BSA solutions. Corona proteins are known to promote interactions with specific cell receptors, as well as to affect the mechanisms by which nanoparticles are internalized by cells^13,16,17^: consistent with this, the different coronas adsorbed on the soft and rigid liposomes are likely to explain the observed differences not only in uptake efficiency, but also in uptake mechanisms, as well as the different involvement of receptors such as LDLR in the uptake of the two liposomes. Further studies are required to fully compare the receptors involved in their uptake, as well as to identify which specific corona proteins or corona patterns may be involved.

In conclusion, the aim of this study was to understand at a fundamental level how nanoparticle mechanical properties affect uptake by cells and in particular determine whether nanoparticle rigidity affects corona formation in serum, an aspect that is - so far - mostly overlooked. To this end, we prepared liposomes of different rigidity by varying the lipid acyl chain saturation and length and optimized their formulation to ensure stability in serum. This resulted in nanoparticles of similar size, zeta potential and surface composition, but very distinct rigidity. The rigid liposomes were generally internalized more than soft liposomes across multiple cell lines and different endocytic pathways were involved in the internalization of the two liposomes in HeLa cells, despite their comparable size and charge.

We next compared the corona adsorbed on the two liposomes in serum and found very clear differences in their composition, particularly in low molecular weight proteins. Of note, while here extensive work was performed to obtain stable liposomes without the addition of PEG in order not to perturb corona formation, based on the differences observed in the corona adsorbed on the soft and rigid liposomes, it would be interesting in future to establish whether nanoparticle mechanical properties may affect the corona formed on pegylated nanoparticles as well. In fact, corona formation has been reported also for pegylated nanoparticles, and it was suggested that it is the nature of the adsorbed corona and the presence of specific stealth proteins such as clusterin to explain their reduce uptake^40^. However, whether this may be further affected by nanoparticle mechanical properties remains to be elucidated.

When comparing the coronas formed on the two liposomes, our results showed that the most abundant corona proteins were comparable for the two liposomes, but their amount was higher on the soft liposomes. Given the lower uptake of these liposomes, these are likely to include proteins reducing uptake by cells, such as stealth proteins or “don’t eat me” signals^40–42^. Instead only APOH was highly abundant on the rigid liposomes, together with multiple low-abundance proteins. Hence, the higher uptake of the rigid liposomes is likely explained by the strong reduction of proteins that may act as stealth proteins in comparison to the soft liposomes, as well as by the enrichment of APOH and the collective action of multiple low-abundance corona proteins that may promote uptake. Future studies aiming at identifying the receptors mediating the uptake of the rigid liposomes may help to further explain the observed uptake differences.

Importantly, by comparing liposome uptake in different media, we clearly showed that the mechanical properties alone do not fully explain the higher uptake of rigid liposomes: only when liposomes were added to cells in the presence of serum, the uptake of the rigid liposomes was much higher than for the soft liposomes. Therefore, the different coronas formed on the two liposomes upon dispersion in serum lead to the observed differences in uptake efficiency. This is a further example illustrating how the modifications nanomedicines encounter upon administration in biological environments strongly affect their biological behavior, hence need to be taken into account when designing new formulations.

## Methods

### Liposome preparation and lipid quantification

Zwitterionic lipids 1,2-dioleoyl-sn-glycero-3-phosphocholine (DOPC), 1,2-dipalmitoyl-sn-glycero-3-phosphocholine (DPPC) and 1,2-distearoyl-sn-glycero-3-phosphocholine (DSPC) along with the anionic lipids 1,2-dioleoyl-sn-glycero-3-phospho-(1’-rac-glycerol) (DOPG), 1,2-dipalmitoyl-sn-glycero-3-phospho-(1’-rac-glycerol) (DPPG), 1,2-distearoyl-sn-glycero-3-phospho-(1’-rac-glycerol) (DSPG) were purchased from Avanti Polar Lipids and dissolved in chloroform. Various liposome formulations were prepared by mixing the zwitterionic and anionic lipids in different molar ratios. The fluorescent lipophilic dye 1,1’-Dioctadecyl-3,3,3’,3’-Tetramethylindocarbocyanine Perchlorate (DiI; Thermo Fisher Scientific) was incorporated at 0.5 mol% of the total lipid mixture. Unilamellar liposomes of 100 nm were prepared using the thin layer evaporation and extrusion method. In brief, the solvent was evaporated under nitrogen gas and the lipid films were dried overnight in a vacuum desiccator. The lipid films were then resuspended in Dulbecco’s Phosphate Buffered Saline (dPBS; Gibco) to a final lipid concentration of 10 mg/mL. To enhance resuspension, the samples were sonicated in a water bath for 10 min. Uniformly sized liposomes were achieved through 8 freeze-thaw cycles using liquid nitrogen and a water bath, followed by 21 extrusions through a 0.1 µm polycarbonate membrane (Cytiva) with the Avanti Mini-Extruder and Heating block (Avanti Polar Lipids). Notably, the water bath and extrusion were carried out at temperatures at least 10 °C higher above the lipid phase transition temperature (T_m_).

To generate a calibration curve based on DiI fluorescence, a portion of the suspension before extrusion was serially diluted in PBS. For each dilution, 60 µL was added in duplicate to a 96-well black plate (Greiner), and the fluorescence intensity was measured using a BioTek Synergy H1 plate reader (Ex = 543 nm, Em = 580 nm, bandwidth  = 10 nm). The calibration curve was obtained based on the fluorescence of  the samples at known concentrations, in order to correlate fluorescence intensity with lipid concentration. After extrusion, liposomes were diluted to concentrations within the curve’s range, and the final lipid concentration was calculated according to the calibration curve.

### Size distribution and zeta potential measurements

The hydrodynamic diameter and zeta potential of the liposomes were measured using a Malvern ZetaSizer Nano ZS (Malvern Instruments). Dynamic light scattering (DLS) was used to obtain the size distribution. Liposomes were diluted in water, PBS and complete Medium (cMEM; MEM supplied with 10% FBS) at final concentrations of 100 µg/mL. Each sample was measured at least 3 times, comprising a minimum of 10 runs per measurement, at 20 °C using disposable folded DTS-1070 capillary cells (Malvern Instruments). The dispersion in cMEM was also incubated in incubators at 37 °C with 5% CO_2_ for 4 h or 24 h and then measured at 37 °C to assess liposome stability in cell culture conditions.

### Nanoparticle tracking analysis

Liposomes were diluted in PBS approximately 100,000 to 200,000 times to achieve a concentration of around 100–200 particles per measuring frame. Around 1 mL of diluted liposome suspension was injected into Zetaview X30 MONO (Particle Metrix). Each measurement consisted of 2 cycles, scanning 11 cell positions per cycle and capturing 30 frames per position, with Sensitivity: 72 and Shutter: 100 at 25 °C. The recorded video was analyzed by the built-in software with the following settings: Maximum Area: 1000, Min area: 10, Brightness: 25, Classes/Decade: 64.

### Determination of phase transition temperature by light scattering

Liposome suspensions in water were added into a Zen 0040 cuvette and measured using the Malvern ZetaSizer Nano ZS with 173° backscatter light at 25 °C to record the default position and attenuation for optimal size measurement. These values were applied to subsequent scanning at all temperatures. The temperature was increased from 25 to 55 °C in 1 °C intervals, with a 3-minute equilibration time at each temperature. Data were collected as the average of the derived count rate from 3 repeated measurements at each temperature and fitted using a nonlinear regression with a Boltzmann sigmoidal model (Eq. 1) in order to calculate the transition temperature^30^:

1 $$y=\frac{{A}_{1}-{A}_{2}}{1+{e}^{(X-{X}_{0})/\Delta X}}+{A}_{2}$$

where *A*_*1*_ is the initial *y* value (count rate of lipids in phase 1); *A*_*2*_ is the final *y* value (count rate of lipids in phase 2); *X*_*0*_ is the center of the distribution, where the *y* value at *X*_*0*_ is halfway between the two extreme values *A*_*1*_ and *A*_*2*_: $y\left({X}_{0}\right)=({A}_{1}+{A}_{2})/2$; and Δ*X* is the width of the slope.

### Determination of phase transition temperature by differential scanning calorimetry (DSC)

DPPC liposomes were prepared as a control. Around 20 µL of either DPPC or DPPC/G liposomes were encapsulated in sealed aluminum DSC pans. The thermal behavior of the liposomes was analyzed using a TA Instruments Q1000 DSC calibrated with indium. The samples were subjected to a heating scan ranging from 10 to 70 °C at a rate of 5 °C per minute in a nitrogen atmosphere.

### Determination of membrane fluidity by monitoring laurdan fluorescence

Laurdan was incorporated at a 1% molar ratio into the lipid film, excluding the DiI dye. Liposomes were prepared following the protocol described above. Liposomes were diluted in DI water to a concentration of around 0.4 mg/mL and added to a black 96 well plate with transparent bottom (Greiner). Laurdan emission spectra were obtained between 400 and 600 nm (excitation at 340 nm, step size of 10 nm, bandwidth of 9 nm) using a BioTek Synergy H1 plate reader. The scans were conducted with the temperature increasing from 30 to 50 °C in 1 °C intervals, allowing a 3-min equilibration time at each temperature. The general polarization (GP_340_) was calculated using the following Eq. (2)^34^:

2 $${{GP}}_{340}=\,\frac{{I}_{440}-{I}_{490}}{{I}_{440}+{I}_{490}}$$

where ${I}_{440}$ and ${I}_{490}$ are the fluorescence detected at 440 and 490 nm when excited with 340 nm light.

### Atomic force microscopy imaging

The Atomic Force Microscopy (AFM) study was conducted using the JPK Nanowizard ultra-speed AFM in quantitative imaging (QI^TM^) mode under physiological salt conditions. We employed qp-BioAC CB3 probes (Nanosensors) with spring constants ranging between 0.03 and 0.09 N/m. Prior to measurements, cantilevers were calibrated using the contact-based and thermal noise methods implemented in the JPK Nanowizard control software (version 6). Coverslips were pre-coated with poly-L-lysine (PLL) by immersing them in a 0.001% PLL solution for 10 min, followed by 2 times rinsing with DI water and overnight drying at 37 °C^52^. For the AFM imaging experiments, 20 µL of 100-fold diluted liposomes in PBS was loaded at the center of the coverslip, with PBS added around it as an imaging buffer. Imaging started directly after the addition of PBS. The liposomes were tested within two days of preparation to ensure freshness and accuracy of the measurements.

To determine liposome deformation, QI images were acquired with imaging forces increasing from 70 pN to 700 pN (up to 80 pN for DOPC/G liposomes). The pixel size (128 × 128 nm^2^) and pixel time (15 ms) were kept constant. The acquired images were subsequently analyzed using JPK Data Processing (JPK-DP) version 6.1 for baseline and background leveling. Further analysis was performed using Gwyddion software (version 2.58) and custom scripts to correct for the cantilever drift in the AFM images and to determine the maximum height of the liposomes at each imaging force^53,54^. A minimum height of 20 nm was set as the criterion for identifying intact liposomes. For DOPC/G liposomes, their height was recorded at imaging forces of 70 and 80 pN, as most collapsed at higher imaging forces. In contrast, for DPPC/G liposomes, only particles that remained intact at the same position and did not collapse on the surface after at least 3 consecutive forces were recorded.

Due to—among others—pressure built up of the adhered vesicles^36^, elastic parameters as deduced from force indentation curves, do not necessarily reflect elastic parameters of the undeformed particles. However, an apparent modulus, here denoted as *M*_*app*_, can be deduced to compare relative elastic parameters between surface adhered liposomes with identical head groups and adhered to the same type of surface. Determination of the apparent modulus was performed by QI-Advanced imaging mode. Each imaging pixel represents a single nanoindentation experiment in QI mode, and QI-Advanced imaging can record those curves for each pixel. The original imaging parameters (loading rate, Z length, sampling, etc.) were kept constant. Imaging was performed at imaging forces of ~100 pN and both topography images and the force curves were processed using the JPK-DP software. The raw force curves were adjusted by subtracting the baseline, and adjustment of the point of contact and the vertical tip position. These processed curves were fitted with the Hertz/Sneddon model built in the JPK-DP, assuming a cone indenter, a half-cone angle of 15 degrees, tip radius of 10 nm and a Poisson’s ratio of 0.5. OriginPro version 2021 (OriginLab Corporation, Northampton, MA, USA) was used for further data processing and statistical analysis.

### Cell culture

Human cervical carcinoma cells HeLa (ATCC CCL-2) and A549 (ATCC CCL-185) were cultured in cMEM under standard conditions of 37 °C and 5% CO_2_. THP-1 (ATCC TIB-202) cells were cultured in RPMI1640 medium (Gibco) supplemented with 10% FBS, 2 mM L-glutamine and 20 mM HEPES (Sigma-Aldrich). SH-SY5Y (ATCC CRL-2266) cells were cultured in DMEM/F12 1:1 medium (Gibco) supplemented with 10% FBS. Cells were grown in T75 flasks and passaged every 2–4 days till maximum 20 passages after defrosting. Regular mycoplasma tests were performed to exclude mycoplasma contamination.

### Liposome uptake studies by flow cytometry

HeLa or A549 cells were seeded in 24-well plates (Greiner) at a density of 50,000 cells per well. THP-1 cells were seeded at a density of 100,000 cells per well and incubated overnight with 100 nM phorbol 12-myristate 13-acetate (PMA) in cell culture medium or medium only for PMA-stimulated THP-1 (sTHP-1) and monocytic THP-1 (mTHP-1), respectively. SH-SY5Y cells were seeded at a density of 120,000 cells per well. After 24 h incubation, the cells were treated with liposomes in cMEM at 37 °C and 5% CO_2_. Cells were first incubated with liposomes for 5 h at varying concentrations to study how uptake changes with concentrations. Subsequently, for the kinetics study, cells were incubated with 100 µg/mL liposomes for the indicated time periods.

Subsequently, cells were washed once with cMEM and twice with PBS to remove excess liposomes outside cells. Then cells were harvested by trypsinization using 0.05% trypsin-EDTA for 4 min at 37 °C. Following trypsinization, the cells were centrifuged at 300 rcf for 5 min. The resulting cell pellets were resuspended in 100 µL PBS and subjected to flow cytometry measurement using CytoFLEX S (Beckman Coulter). Gates were set in the forward and side scattering plots to exclude cell debris and doublets as shown in Supplementary Fig. 6. At least 15,000 single cells were acquired for each sample, unless otherwise indicated. Data were analyzed with FlowJo software (FlowJo, LLC) to calculate the average and standard deviation of the median cell fluorescence intensity (MFI) over replicates.

### Fluorescence imaging

HeLa cells were seeded in 4-chamber 35 mm petri dish (Greiner Bio-One) at a density of 50,000 cells per chamber. After 24 h incubation, cells were treated with 100 µg/mL liposomes in cMEM at 37 °C and 5% CO_2_ for 5 h. To visualize lysosomes and nuclei, cells were stained with 500 nM Lysotracker Green DND-26 (Thermo Fisher Scientific) for 30 min, followed by incubation with 1 µg/mL Hoechst 33342 (Thermo Fisher Scientific) for 5 min. To guarantee better signal, cells were kept in 10 nM Lysotracker during imaging. Images were acquired using Zeiss Cell Discovery 7 (Zeiss) with a 50X Objective, 0.5X Optovar. The channels for each dye were set as Ex = 348 nm, Em = 455 nm, Detection Wavelength 400–500 nm for Hoechst 33342; Ex = 503 nm, Em = 509 nm, Detection Wavelength 450–540 nm for Lysotracker Green DND-26; Ex = 551 nm, Em = 569 nm, Detection Wavelength 547-700 nm for DiI (liposomes). ImageJ Fiji software^55^ was used for image processing (adding scale bar and splitting image channels). To visualize plasma membrane and nuclei, cells were stained with 3 µg/mL Hoechst 33342 (Thermo Fisher Scientific) for 7 min, followed by incubation with 1 µg/mL CellMask Deep red plasma membrane stain (Thermo Fisher Scientific) for 5 min. Cells were kept in phenol red-free cMEM during imaging. Images were acquired using Zeiss Cell Discovery 7 (Zeiss) with a 50X Objective, 1X Optovar. The channel for CellMask Deep red plasma membrane stain was set as Ex = 659 nm, Em = 676 nm, Detection Wavelength 620-700 nm.

### NaN_3_, chemical inhibitors and competition studies

In order to determine whether uptake was energy dependent, cells were incubated in cMEM with 5 mg/mL sodium azide (NaN_3_) for 30 min to deplete cell energy, followed by exposure to 100 µg/mL liposomes in the presence of NaN_3_ for 5 h. Untreated cells incubated with the liposomes in standard conditions served as the control group.

In order to characterize the mechanism of liposome uptake, cells were pretreated with various inhibitors targeting specific endocytic pathways. Cells were pre-incubated with the different inhibitors as follows: chlorpromazine (Sigma-Aldrich, 10 µg/mL, 20 min) to test for clathrin-mediated endocytosis, EIPA (Tocris, 50 µM, 20 min) for macropinocytosis inhibition, cytochalasin D (Focus Biomolecules, 2.5 µg/mL, 20 min) to disrupt actin polymerization, nystatin (Merck, 25 µg/mL, 30 min) for inhibiting caveolin-mediated endocytosis, and hydroxydynasore (MCE, 10 mg/mL, 30 min) to block dynamin-dependent uptake mechanisms. Dextran sulfate (Sigma Aldrich, 10 µg/mL) was pre-incubated with liposomes for 30 min prior to addition to cells to assess whether the uptake involved interactions with cell surface heparan sulfate. Following the pre-incubation, the liposomes (100 µg/mL) were added to the cells in the presence of the inhibitors for 5 h. Untreated cells incubated with the liposomes in standard conditions served as the control group. To confirm stability, liposomes (100 µg/mL) were incubated in cMEM with or without each inhibitor for 5 h, and the size distribution was measured using a Malvern ZetaSizer Nano ZS.

### Corona isolation and characterization (lipid & protein quantification; SDS-PAGE)

To isolate corona-coated liposomes, 1 mg lipid/mL of liposomes was dispersed in full FBS, maintaining the same protein-liposome surface area ratio as used for experiments with cells (100 µg/mL liposomes in medium with 10% FBS). Samples were incubated in a Thermo-shaker (Grant Institute) at 37 °C and 300 rpm for 1 h. Size Exclusion Chromatography (SEC) using a Sepharose CL-4B column (Sigma-Aldrich) was employed to separate liposomes from excess FBS proteins. After incubation at 37 °C, the samples were loaded onto the column and eluted with PBS. MEM without Phenol Red (Gibco) was used for elution if the samples were intended for subsequent cell experiments. A total of 30 eluted fractions of 0.5 mL each were collected. To determine the presence of proteins and liposomes, each fraction was tested using a BioTek Synergy H1 plate reader. Three microliters of each fraction were loaded on a Take-3 plate, and absorbance at 280 nm along with DiI fluorescence (Ex = 543 nm, Em = 580 nm, bandwidth =10 nm) were determined in order to monitor elution of proteins and liposomes, respectively. Fractions exhibiting both absorption of proteins and liposome fluorescence signals were pooled together. The pooled liposome concentration was detected by measuring the fluorescence of DiI in a BioTek Synergy H1 plate reader and calculated according to the calibration curve established during the liposome preparation process. Isolated corona-coated liposomes were diluted in PBS to a final concentration of 100 µg/mL lipids, and their size distribution and zeta potential were measured using a Malvern ZetaSizer Nano ZS.

In order to compare the protein-binding capacity of the different liposome formulation, the pooled fractions were concentrated by centrifugation at 4000 rcf using Amicon 10 kDa ultra centrifugal filter tubes (Merck) until the final volume was less than 100 µL. The final lipid concentration was determined based on DiI fluorescence as described previously. The protein concentration was determined using the DC protein assay kit (Bio-Rad Laboratories, Inc.). In brief, 5 µL of sample was mixed with 25 µL reagent A’ (containing 20 µL reagent S in 1 mL reagent A) and 200 µL reagent B successively. After 15 min incubation, absorbance was measured at 750 nm and compared with a calibration curve created from serial diluted BSA in PBS to calculate the protein concentration. The protein/lipid ratios were then determined. The average and standard deviation of the results from 4 independent experiments were calculated.

In order to separate and visualize the proteins present in the different liposome coronas, sodium dodecyl sulfate-polyacrylamide gel electrophoresis (SDS-PAGE) was applied. Samples corresponding to 0.15 μmol lipid or 15 µg protein were mixed with 4x loading buffer (200 mM Tris-HCl, 400 mM DTT, 8% SDS, 0.4% bromophenol blue and 40% glycerol) and heated at 95 °C for 5 min before loading onto a 10% polyacrylamide gel. FBS (15 µg) was loaded as a control and Precision Plus protein all blue standard (Bio-Rad Laboratories, Inc.) was used as a ladder. The gel was run at 80 V for 20 min, then at 120 V until the samples reached the bottom. Then the gel was stained with 0.1% Coomassie blue R-250 in water/ethanol/acetic acid solution (5:4:1, v/v) in a microwave (800 W) for 1 min, followed by room temperature shaking for 15 min. Excess staining was removed by heating the gel in hot water and incubating in water/ethanol/acetic acid solution (5:5:1, v/v) overnight for better contrast. Images were captured using a ChemiDoc XRS (Bio-Rad Laboratories, Inc.).

### Untargeted proteomics analysis

Corona protein samples underwent in-gel digestion for proteomics with label-free quantification. Briefly, 20 µg samples were diluted in 100 mM ammonium bicarbonate (ABC) to reach total volume of 30 µL and mixed with 10 µL of 4X LDS loading buffer (Abcam). The samples were incubated at 70 °C for 10 min, then loaded onto a 4-12% Bis-Tris Gel (Thermo Fisher Scientific) and run in MES buffer at 100 V for 5 min. The gel was stained with a MS-compatible Coomassie stain (Instant blue, Thermo Fisher Scientific) for 1 h to visualize protein-containing bands. These bands were excised from gel, cut into small pieces, and destained using a sequence of 30% acetonitrile (ACN) in ABC for 30 min, 50% ACN in ABC for 30 min, 100% ACN. The gel pieces were then dried. Proteins within the gel pieces were reduced with 10 mM DTT in ABC at 55 °C for 30 min, followed by alkylation with 55 mM iodoacetamide in ABC at room temperature in the dark for 30 min. The gel pieces were dehydrated with 100% ACN and dried at 37 °C for 30 min. Proteins were digested with sequencing grade modified trypsin (Promega) at a 1:100 dilution at 37 °C overnight. The resulting peptides were extracted from the gel pieces using 5% formic acid in 75% ACN for 20 min. The supernatant was collected and organic solvent was removed with a Speedvac concentrator before injection into the HPLC system. The digested samples were analyzed by Evosep system (Evosep One; Evosep) linked with a quadrupole orbitrap mass spectrometer equipped with a nano-electrospray ion source (Orbitrap Exploris 480; Thermo Fisher Scientific). The mass spectrometer operated in data-independent acquisition mode (DIA) with isolation windows of 16 m/z and a precursor mass range of 400 to 1,000 m/z. LC-MS raw data were processed with Spectronaut (Biognosys) using the standard directDIA workflow, with a bovine Swiss-Prot database (www.uniprot.org, 6047 entries, accessed November 2023). Label-free quantification (LFQ) and intensity-based absolute quantification (iBAQ) were separately applied on raw protein intensities. Local Normalization was applied, and the Q-value filtering was set to the classic setting without imputing. The mass spectrometry data have been deposited to the ProteomeXchange Consortium via the PRIDE^56^ partner repository with the dataset identifier PXD079018.

 Proteins identified in at least 2 out of 3 replicates within each group were selected. Venn diagrams were plotted on Venny 2.1 website^57^. To compute protein abundance, the relative iBAQ (riBAQ) value was calculated by dividing a protein’s iBAQ by the sum of all iBAQ values for all identified proteins within a sample^58^. Rank product method was applied to rank the most abundant proteins within each group^59^. The obtained rank product values for each protein were then sorted from low to high, providing the protein ranking which is displayed in the plots. Bubble plots were used to visualize the top 20 most abundant proteins using R (version 4.3.0) and R studio (version 2023.03.1). The balloon area shows the protein abundance and the colors from black to red correspond to the *p*-values of the one-way ANOVA analysis between soft and rigid samples.

To compare each identified protein between groups, statistical analysis was performed using LFQ intensities. Protein intensity values were log₂-transformed prior to analysis. The Principle Component Analysis (PCA) plot was exported from Spectronaut to visualize sample differences between and within groups. Sample-to-sample similarity was evaluated by calculating Pearson’s correlation coefficients (r) based on the log₂-transformed intensities by Perseus (version 2.0.10.0). Pearson’s r was computed as the covariance between two samples divided by the product of their standard deviations. Differential proteins were identified in Perseus using a two-sided t-test with permutation-based false discovery rate correction (FDR = 0.05, s0 = 0.1), and a volcano plot was drawn to visualize the results.

### RNA interference

Silencer Select siRNAs were purchased from Thermo Fisher Scientific. Scrambled siRNA (Silencer Select negative control no.1) served as a negative control. To silence the expression of endocytosis-related proteins, 13,000 HeLa cells were seeded in 24-well plates 24 h prior to transfection. For each well, 10 pmol siRNA was mixed with 1 µL Oligofectamine (Thermo Fisher Scientific) and incubated at room temperature for 20 min. The cells were pre-washed with SF-MEM and then exposed to Oligofectamine-siRNA complex, which was diluted in SF-MEM to a total volume of 250 µL. After 5 h incubation, MEM supplemented with 30% FBS was added to achieve a final concentration of 10% FBS. Three days after transfection, the cells were exposed to 100 µg/mL liposomes for 5 h before being harvested for flow cytometry analysis.

### LDL competition

To assess the competitive effect of low-density lipoprotein (LDL) on liposome uptake, HeLa cells were seeded at a density of 50,000 cells per well in a 24-well plate one day prior to the experiment. The number of liposomes was estimated based on their theoretical radius (50 nm) and density (1 g/cm^3^), yielding approximately 1.91 ×  10^11^ liposomes per 100 µg. For the competition study, 100 µg/mL liposomes were co-incubated with unlabeled LDL (Biovision) at varying number excess ratios, where 1× LDL corresponded to 0.73 µg/mL. The incubation was performed in cMEM at 37 °C for 5 h. Liposomes (100 µg/mL) were incubated in cMEM with or without 25x LDL (18.25 µg/mL) for 5 h, and the size distribution was measured using a Malvern ZetaSizer Nano ZS.

To rule out potential biases introduced by the experimental design, we conducted the experiments under four different pre-incubation conditions using a fixed concentration of 25x LDL (18.25 µg/mL): (1) LDL pre-incubation with cells at 37 °C for 30 min, (2) LDL pre-incubation with liposomes at 37 °C for 30 min, (3) no pre-incubation; then LDL and liposomes (100 µg/mL) were added to cells simultaneously, followed by incubation at 37 °C for 5 h.

### Pre-coated liposome uptake

HeLa cells were seeded at a density of 25,000 cells per well in a 48-well plate one day prior to the experiment. APOH (Thermo Fisher Scientific) or BSA (Merck) was added to liposomes at a ratio of 1 mg protein per 1 m² liposome surface area (corresponding to 6 µg protein with 100 µg liposomes)^60^, and was incubated in a thermo-shaker at 37 °C and 300 rpm for 1 h to form APOH/BSA -coated liposome. Protein coated liposomes were incubated with HeLa cells at 50 µg/mL in cMEM or serum-free MEM for 5 h.

### APOH competition

Corona-coated liposomes were obtained by SEC as described before. To assess the competitive effect of APOH, cells were first pre-incubated with different concentrations of APOH at 4 °C for 30 min, allowing APOH occupying potential cell surface receptors. Following pre-incubation, HeLA cells were incubated with 50 µg/mL liposomes or corona-coated liposomes in cMEM containing different concentrations of APOH (corresponding to the pre-incubation concentrations) for 5 h.

### Uptake in different media

To compare liposome uptake behavior in different media, HeLa cells were incubated with liposomes in serum free medium, or in medium containing 4 mg/mL BSA, FBS, or human serum for 5 h. In addition, corona-coated liposomes were isolated and added to cells in either serum free medium or in medium with 4 mg/mL FBS (in all cases, liposomes and corona-coated liposomes were added to cells at 100 µg/mL lipid) for 5 h. For uptake kinetics, HeLa cells were incubated with 100 µg/mL liposomes in serum free medium, or in medium containing 4 mg/mL BSA, FBS for different times.

### Reporting summary

Further information on research design is available in the Nature Portfolio Reporting Summary linked to this article.

## Supplementary information

Supplementary Information

Description of Additional Supplementary Files

Supplementary Data

Reporting Summary

Transparent Peer Review file

## Source data

Source data

## Data Availability

Source data is available for main Figs. 1, 2, 3, 4, 5, 6, 7, 8 and Supplementary Figs. 1, 2, 3, 4, 5, 6, 7, 8, 9, 10, 11 in the associated Source Data file. The mass spectrometry data have been deposited to the ProteomeXchange Consortium via the PRIDE^56^ partner repository with the dataset identifier PXD079018. Other raw data are available from the corresponding author upon request. Source data are provided with this paper.

## Code availability

A script was developed in Python 3.12 to correct for the cantilever drift in the AFM images and determine the maximum height of the liposomes at each imaging force. The script is deposited at GitHub with DOI identifier 10.5281/zenodo.21605582^54^.
