# Supplementary material for: Effect of liposome rigidity on corona formation and its implications for uptake by cells
Source: Nat Commun. 2026 Sep 1;17:10395. doi: 10.1038/s41467-026-77231-7 (PMC13627682; doi:10.1038/s41467-026-77231-7)

Supplementary Information

Effect of liposome rigidity on corona formation and its implications for uptake by cells

Xinyu Ma, Feng Zhao, Miguel Cantero-Reviejo, Sander de Weerd, Ilaria Squillante, Giuseppe Portale, Wouter H. Roos, Anna Salvati**

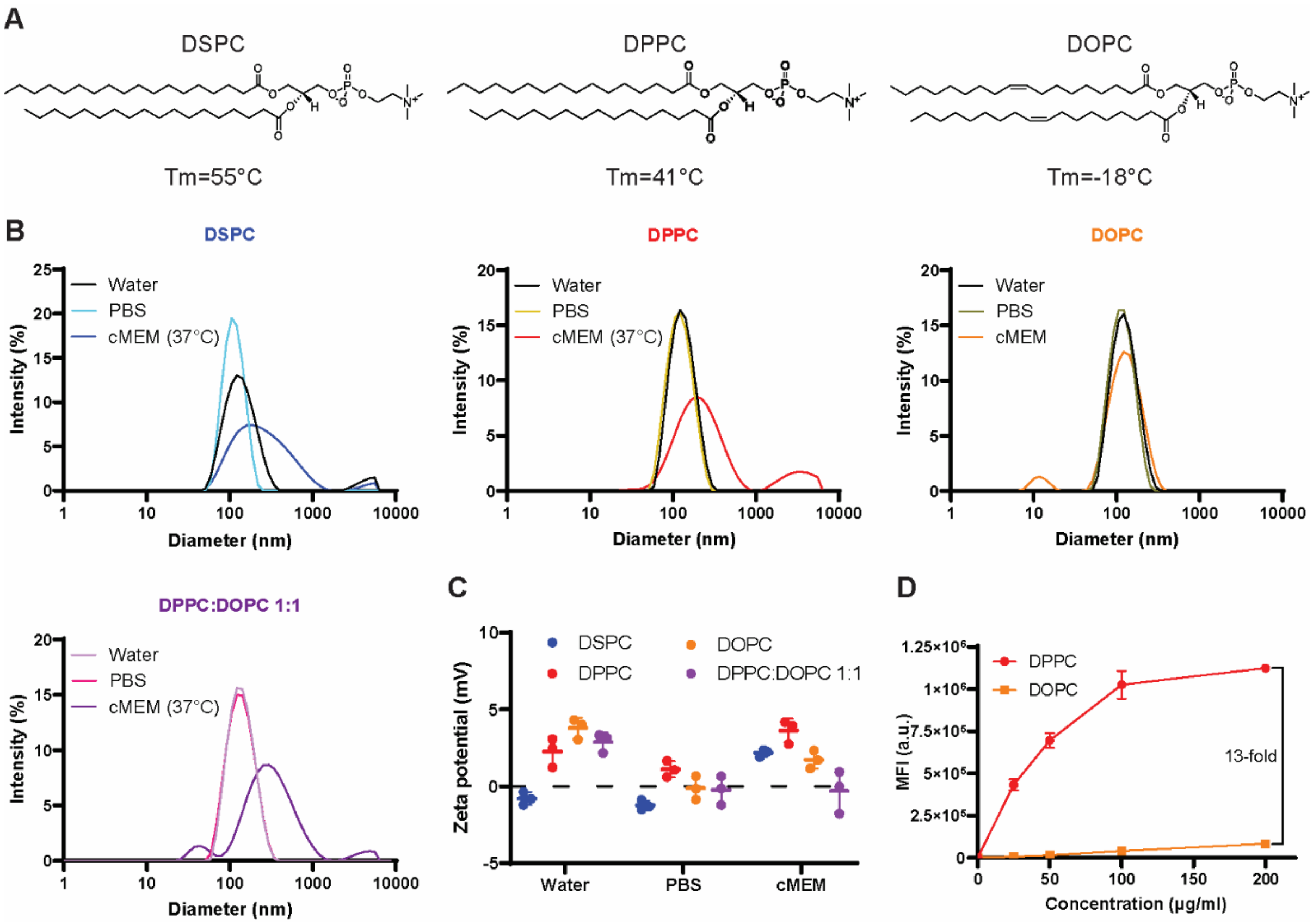

Supplementary Figure 1. Formulation, characterization and uptake of PC liposomes. (A) Chemical structures of DPPC, DOPC and DOPG lipids included in liposomes, together with their structural formulas and transition temperature (T_m). **(B)** Size distribution by intensity (diameter, nm) obtained by DLS and **(C)** zeta potential measurements of liposome dispersions in different media (100 µg/mL liposomes in water, PBS and cMEM at 20° C or 37°C as indicated specifically). In **C**, the results obtained for three technical measurements of each dispersion are shown together with their average and standard deviation. **(D)** Uptake of liposomes at different concentrations. HeLa cells were incubated with different concentrations of DPPC or DOPC liposomes for 5 hours in cMEM**.** Data are presented as mean ± SD of the median fluorescence intensities obtained by flow cytometry for 3 replicate samples.

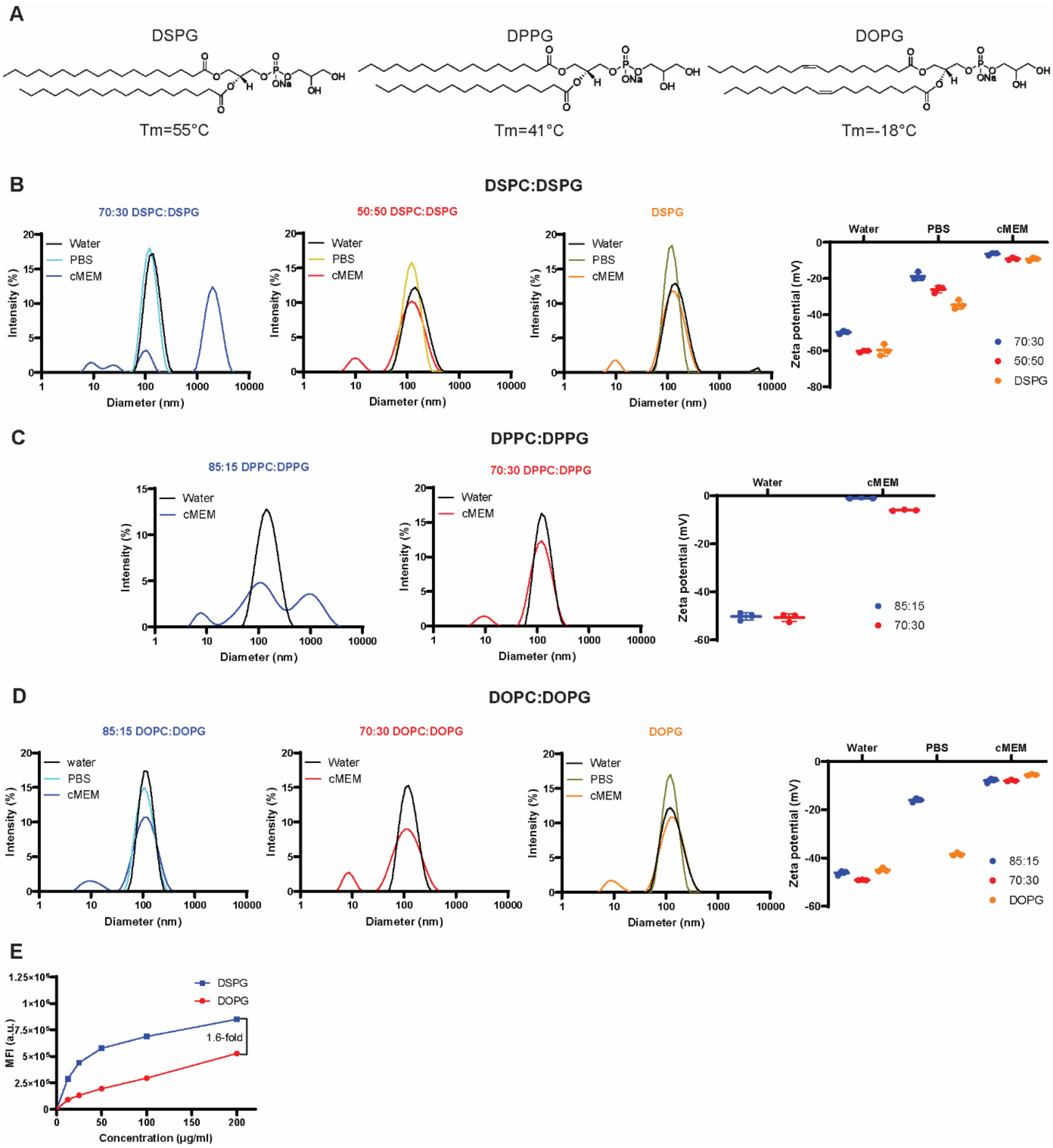

Supplementary Figure 2. Formulation, characterization and uptake of PC/PG liposomes. (A) Chemical structures of DPPG, DOPG and DOPG lipids included in liposomes, together with their structural formulas and transition temperature (T_m). **(B-D)** Size distribution by intensity (diameter, nm) obtained by DLS and zeta potential measurements of **(B)** DSPC:DSPG; **(C)** DPPC:DPPG; **(D)** DOPC:DOPG liposome dispersions in different media (100 µg/mL liposomes in water, PBS and cMEM). Zeta potential results obtained for three technical measurements of each dispersion are shown together with their average and standard deviation. **(E)** Uptake of liposomes at different concentrations. HeLa cells were incubated with different concentrations of DSPG or DOPG liposomes for 5 hours in cMEM. Data are presented as mean ± SD of of the median fluorescence intensities obtained by flow cytometry for 3 replicate samples.

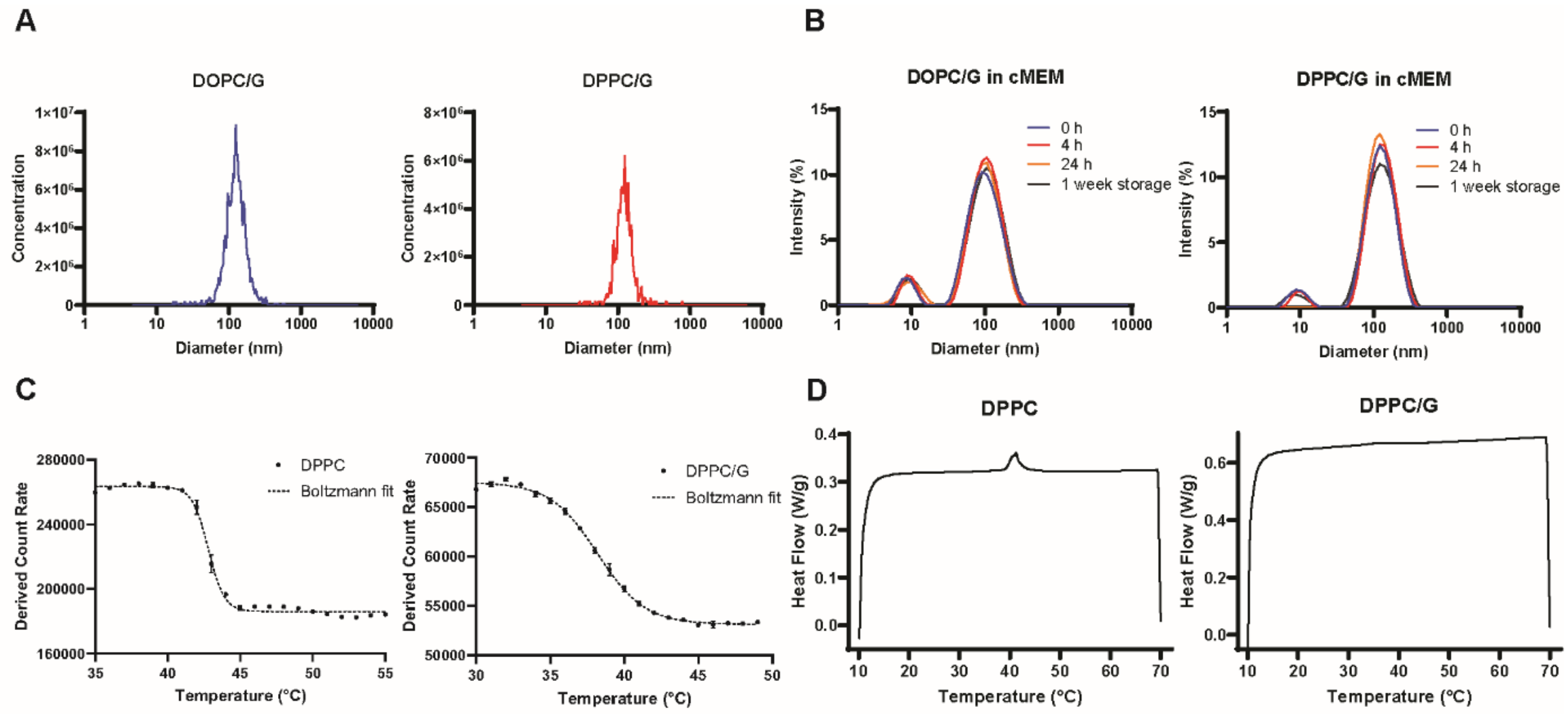

Supplementary Figure 3. Characterization of optimized liposomes. (A) Size distribution of DOPC/G and DPPC/G liposomes in PBS measured by NTA. **(B)** Size distribution of DOPC/G and DPPC/G liposomes (100 µg/mL in cMEM) after 0-, 4- or 24-hours incubation at 37°C and 5% CO_2, or after storage at 4°C for 1 week. **(C)** Derived count rate changes of 1 mM DPPC and DPPC/G liposome in PBS at increasing temperature as obtained by DLS. Each data point is the mean and standard deviation from three independent measurements of the same sample. **(D)** Differential Scanning Calorimetry (DSC) traces of 10 mg/mL DPPC and DPPC/G liposomes in PBS.

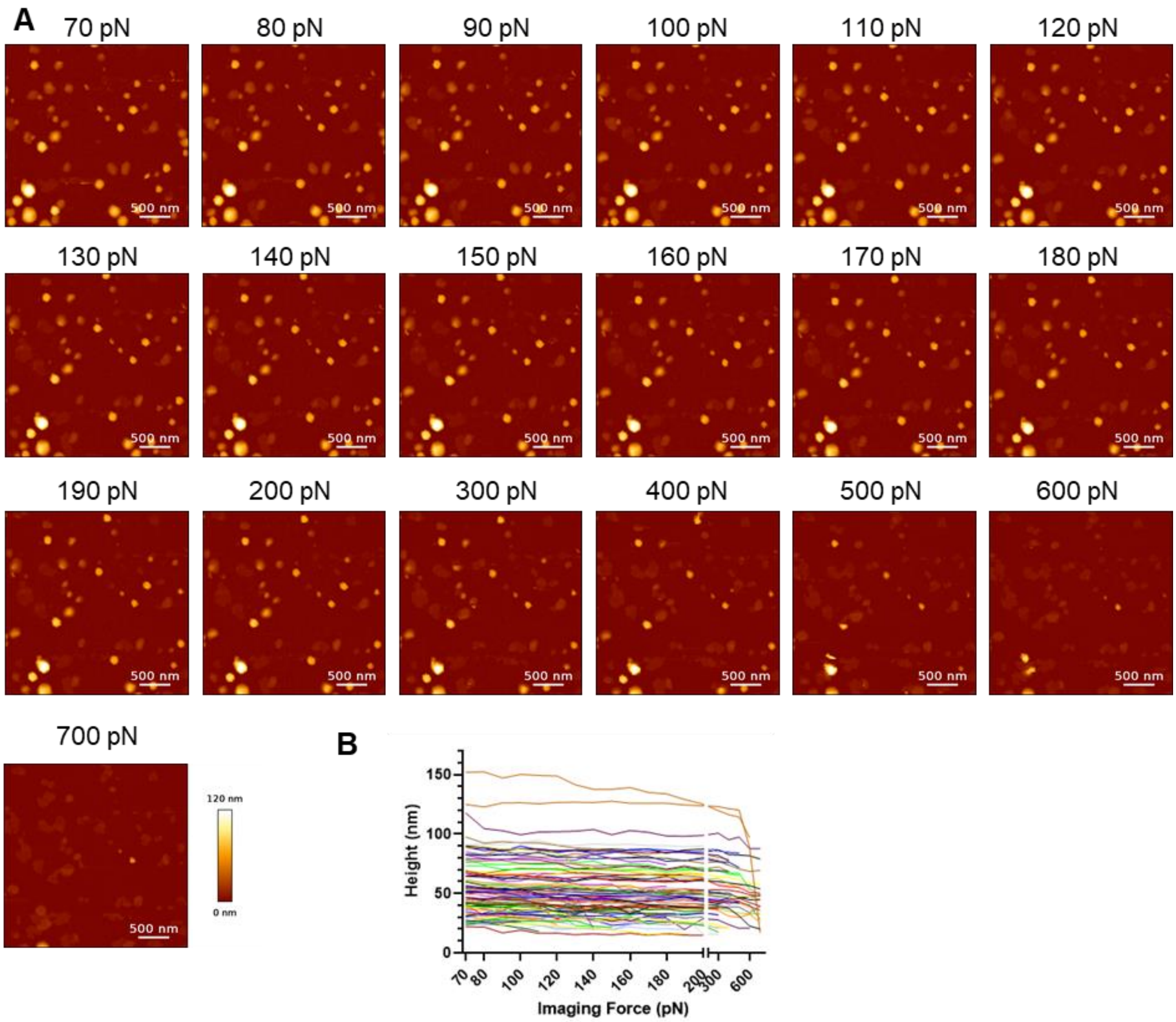

Supplementary Figure 4. Characterization of DPPC/G liposomes mechanical properties by AFM. (A) Topographic images of DPPC/G liposomes under constantly increasing imaging forces from 70 pN to 700 pN. Scale bar: 500 nm. **(B)** Height of individual DPPC/G liposome as a function of the imaging force (n = 88 particles).

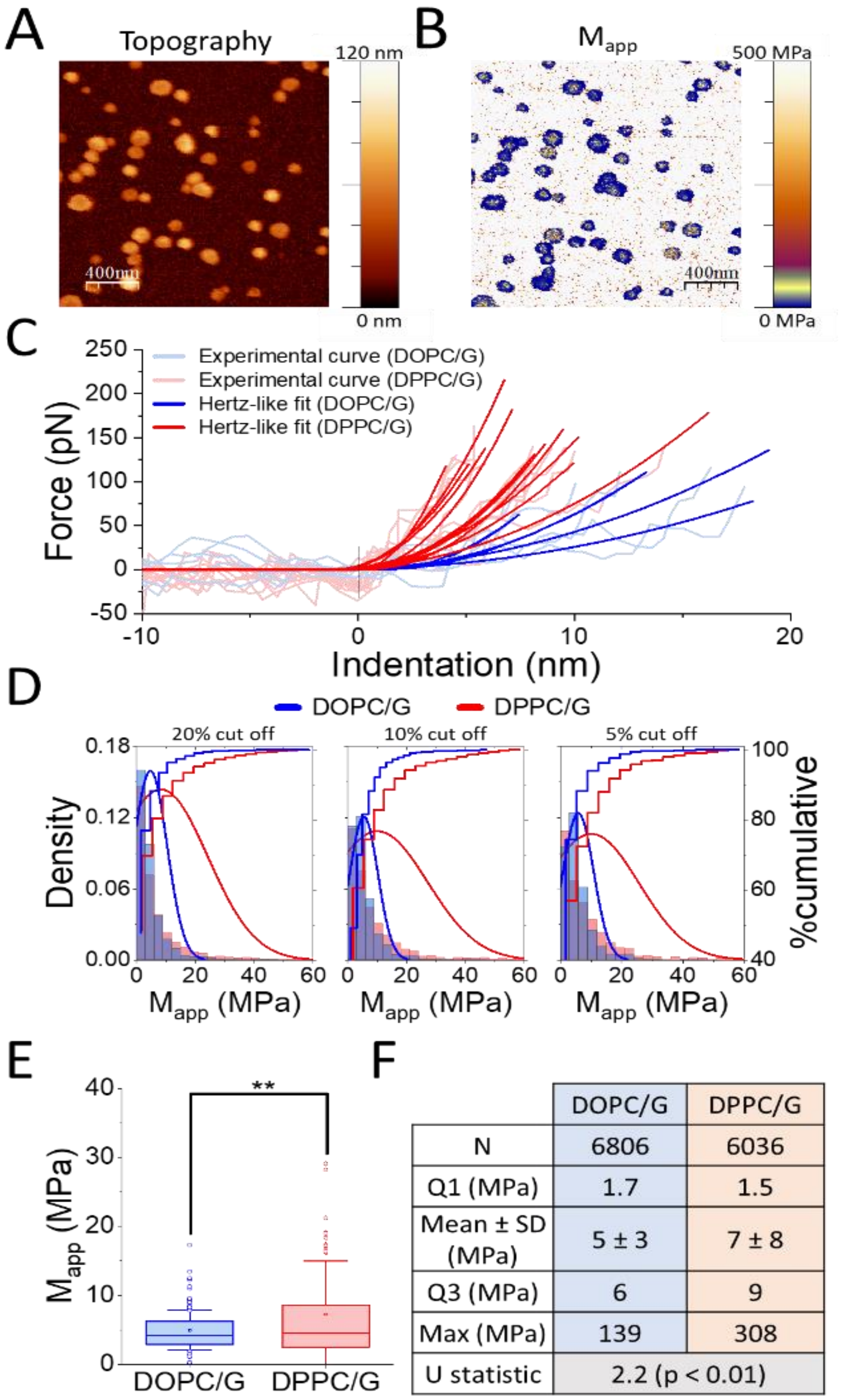

	DOPC/G	DPPC/G
N	6806	6036
Q1 (MPa)	1.7	1.5
Mean ± SD (MPa)	5 ± 3	7 ± 8
Q3 (MPa)	6	9
Max (MPa)	139	308
U statistic	2.2 (p < 0.01)	

Supplementary Figure 5. Characterization of the liposomes by nanoindentation. (A) Example of topographic image and **(B)** apparent modulus M_{app} map of DPPC/G vesicles. **(C)** Example force vs distance curves used for M_{app} determination in each pixel. Shadowed curves are the original data, solid lines depict the fitted Hertz-like model. **(D)** To minimize surface-induced artifacts,[1]only the curves on top of the particles were taken into account and as a control three different cut-off values were compared: Histograms of the M_{app} distribution in DPPC/G (red) and DOPC/G (blue) vesicles for 20%, 10% and 5% of the maximum height. Respectively this corresponded to an average of 39, 23 and 11 pixels per liposome that were used for the M_{app} determination. The binned line shows the cumulative percent of the data distribution. Smooth line shows the gaussian distribution of the data. No statistical difference ($p<0.05$) was observed between the three subgroups for both DOPC/G and DPPC/G particles. Therefore, we selected the 20% cut-off data for the last two figure panels, as these contain more pixels. **(E)** Statistical analysis of the M_{app} of the liposomes using parametric analysis (Tukey's test). Every data point represents the average M_{app} in the 20% cut off for each individual particle. DOPC/G and DPPC/G display a comparable pixel/vesicle ratio, namely ~38 and ~42 respectively. N = 179 and N = 148 for DOPC/G and DPPC/G particles respectively. Data was collected in 3 independent experiments each. **(F)** Non-parametric analysis (Mann-Whitney test) of the data.

We used all M_{app} as data points to study the overall distribution. The N-number in the table is the total number of pixels considered for the respectively 148 (DPPC/G) and 179 (DOPC/G) particles. Both tests show that M_{app} is significantly smaller ($p < 0.01$) for the DOPC liposomes than for the DPPC/G formulation. In the DOPC/G sample, many liposomes (likely the softest and most brittle ones) had already collapsed before force indentation curves could be recorded and therefore the M_{app} of the DOPC/G particles will be mainly based on the more resilient particles. Therefore, the difference in M_{app} between DOPC/G and DPPC/G particles is probably higher than above results let assume.

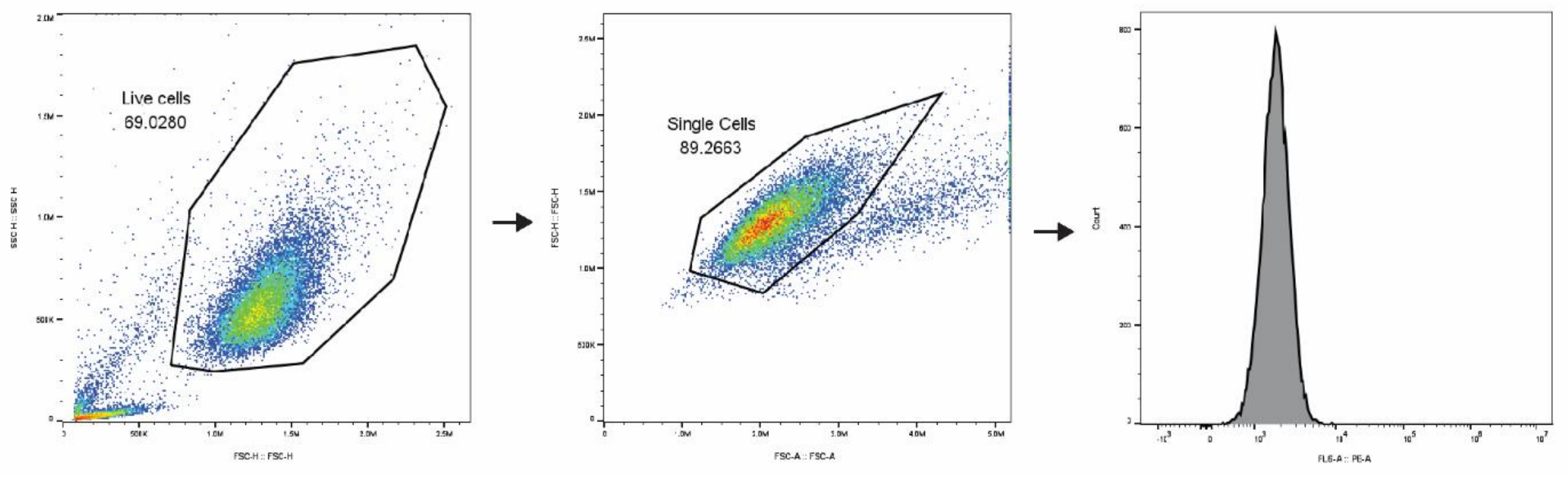

Supplementary Figure 6. Flow cytometry gating strategy. Cells were gated in forward scatter-height (FSC-H) vs side scatter-height (SSC-H), followed by gating for single cells in forward scatter-area (FSC-A) vs FSC-H. The median fluorescence of single cells in the nanoparticle channel (Yellow) was obtained using FlowJo.

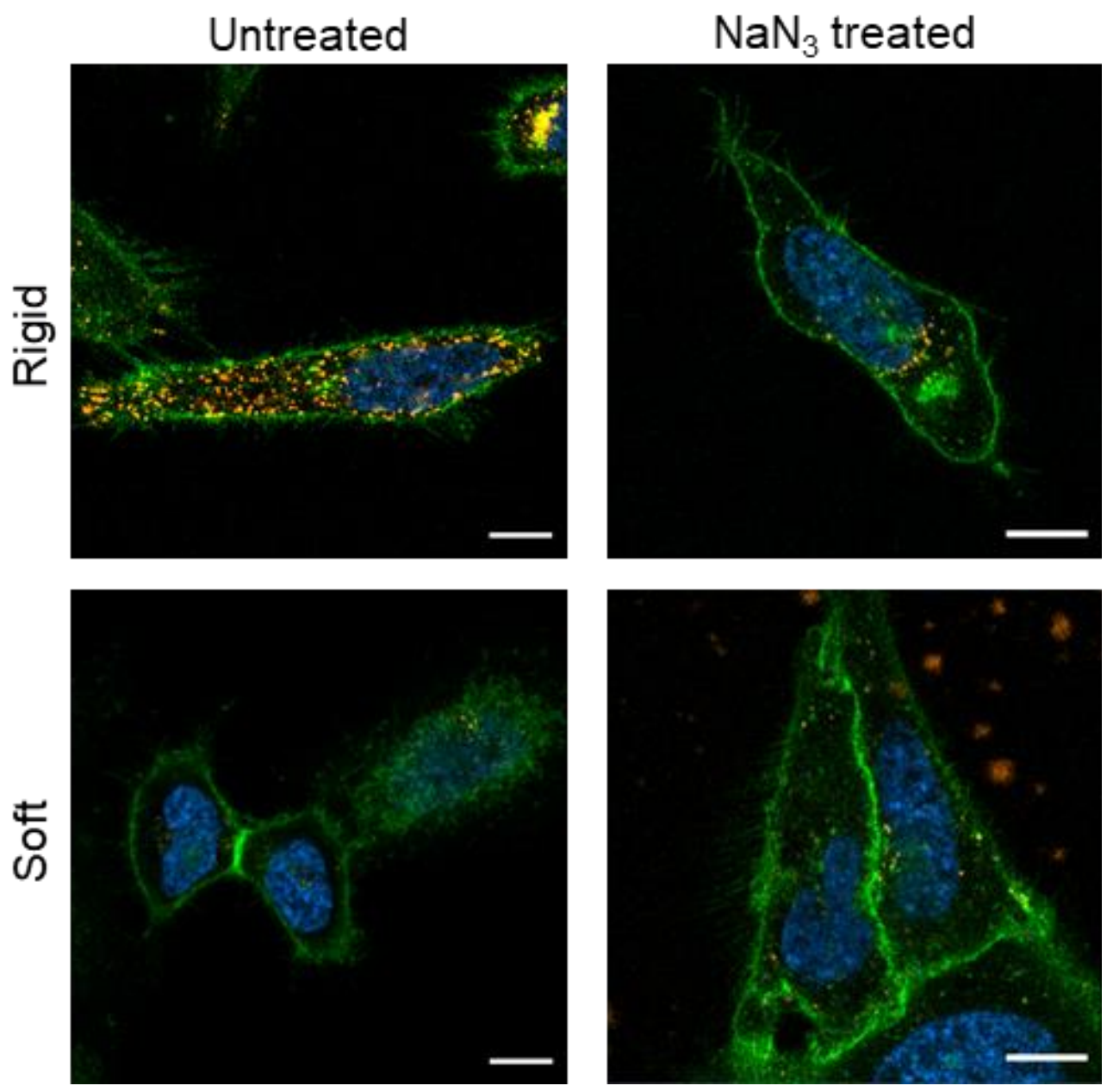

Supplementary Figure 7. Confocal fluorescence images of HeLa cells after incubation with 100 µg/mL rigid or soft liposomes in cMEM in standard conditions (Untreated) or in presence of 5 mg/mL sodium azide (NaN_3 treated) for 5h. Blue: Hoechst-stained nuclei. Red: liposomes. Green: CellMask Plasma Membrane Stain. Scale bar: 10 µm.

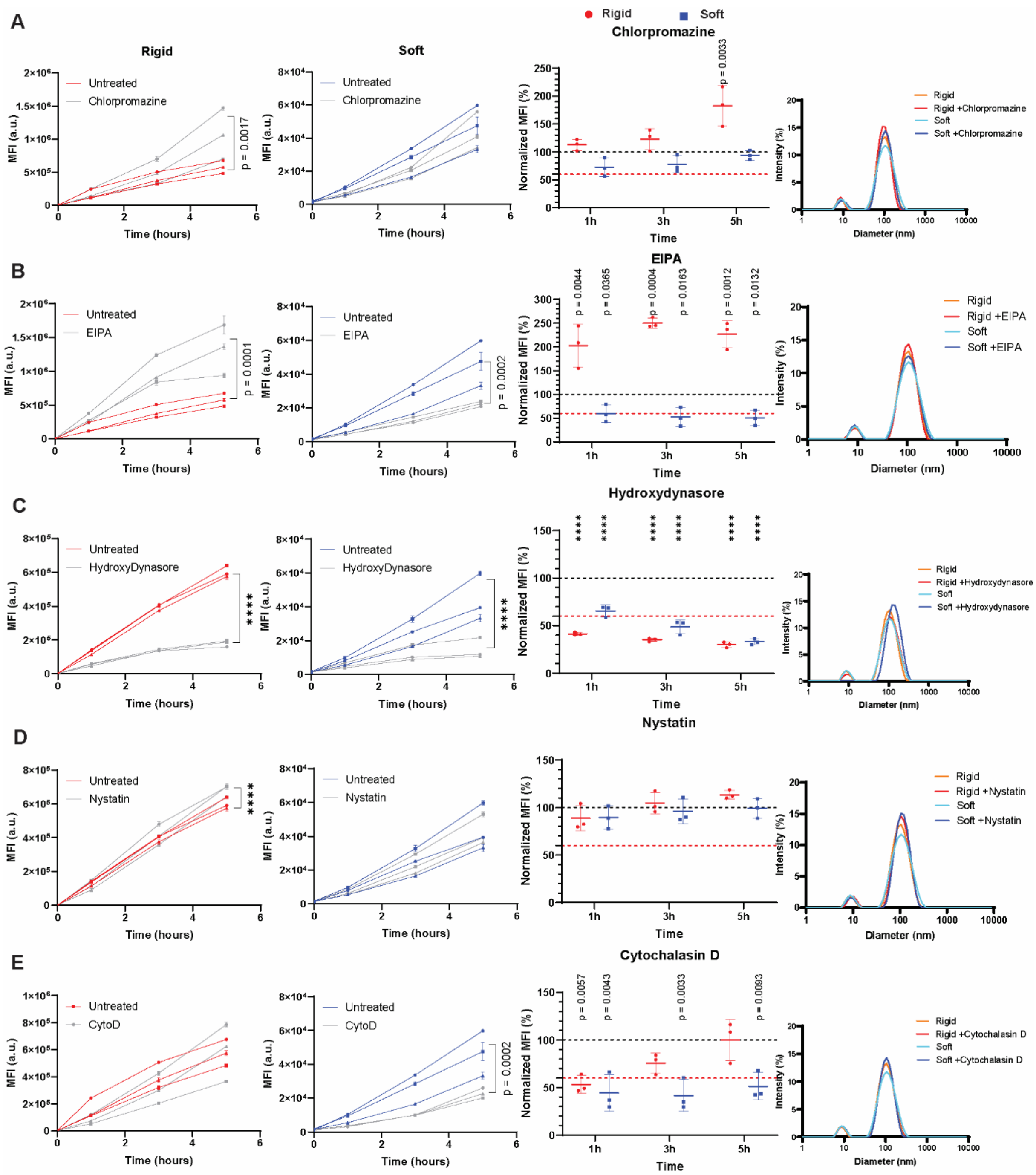

Supplementary Figure 8. Characterization of liposome uptake mechanisms. HeLa cells were incubated with soft or rigid liposomes (100 µg/mL) in cMEM in standard conditions or in the presence of **(A)** chlorpromazine (10 µg/mL); **(B)** EIPA (50 µM); **(C)** hydroxydynasore (10 mg/mL); **(D)** nystatin (25 µg/mL) or **(E)** cytochalasin D (2.5 µg/mL). The results obtained in 3 independent experiments are shown (n = 3), and each data point is the mean ± SD of the MFI from 3 replicate samples. In each line, the same data are shown after normalization for the uptake in standard conditions. The normalized values from 3 independent experiments (n = 3) are presented together with their mean ± SD. A black dashed line and a red dashed line are drawn in each panel as a reference of 100% and 60% uptake respectively, as an indicative threshold for inhibition efficacy. The same normalized data are shown again in Figure 4 in the main manuscript, together with a schematic summary of the findings. The last figure of each line shows the size distribution by intensity (diameter, nm) of 100 µg/mL liposomes after 5-hour incubation in cMEM with or without the presence of inhibitor obtained by DLS. An unpaired two-sided Welch's t-test was used for statistical analysis for the final time point of the kinetics, and an unpaired two-sided one-way analysis of variance (ANOVA) with Dunnett's correction for multiple comparisons was used for normalized data (p values are reported when $p < 0.05$, which was considered significant. ****$p < 0.0001$).

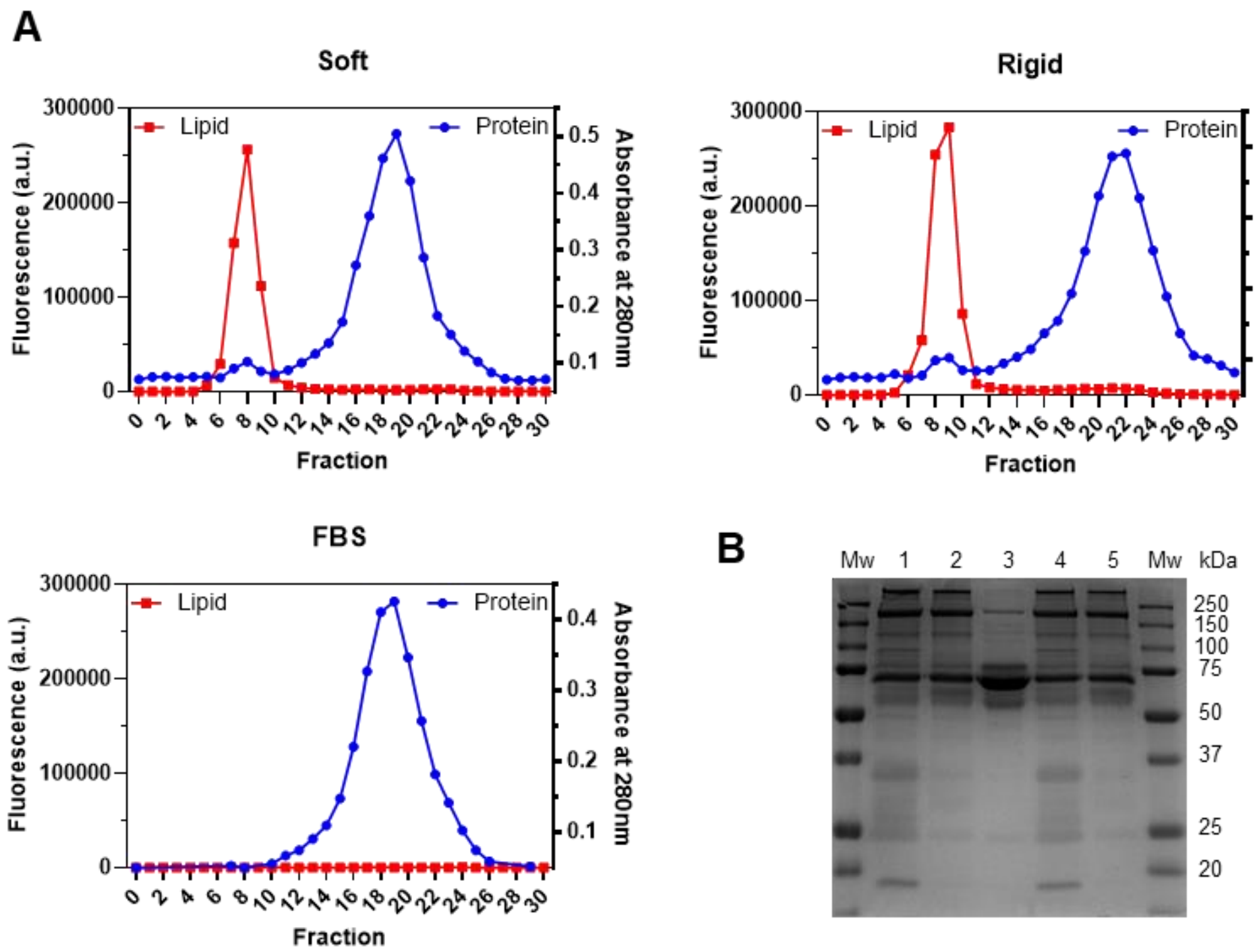

Supplementary Figure 9. Isolation and characterization of corona-coated liposomes. (A) Elution profiles by SEC of soft or rigid liposomes after 1h incubation with full FBS. Briefly, 1 mg liposomes were mixed with 1 mL full FBS, the obtained corona-coated liposomes were separated by SEC and the elution profiles of lipids and proteins were determined. FBS samples eluted by SEC were also tested as a control to exclude contamination of particles of size and density comparable to liposomes. **(B)** SDS-PAGE image of corona proteins adsorbed on rigid and soft liposomes. 15 µg protein (Lane 1: soft, Lane 2: rigid) or 0.15 µmol of lipids (Lane 4: soft, Lane 5: rigid) were loaded in each lane. In addition, 15 µg full FBS was loaded as control (Lane 3). Corona isolation and SDS-PAGE were repeated for a total of 4 independent experiments with similar results and the result of one representative experiment is shown. Part of the same gel (Lanes 3-5) is shown in Figure 5D as well.

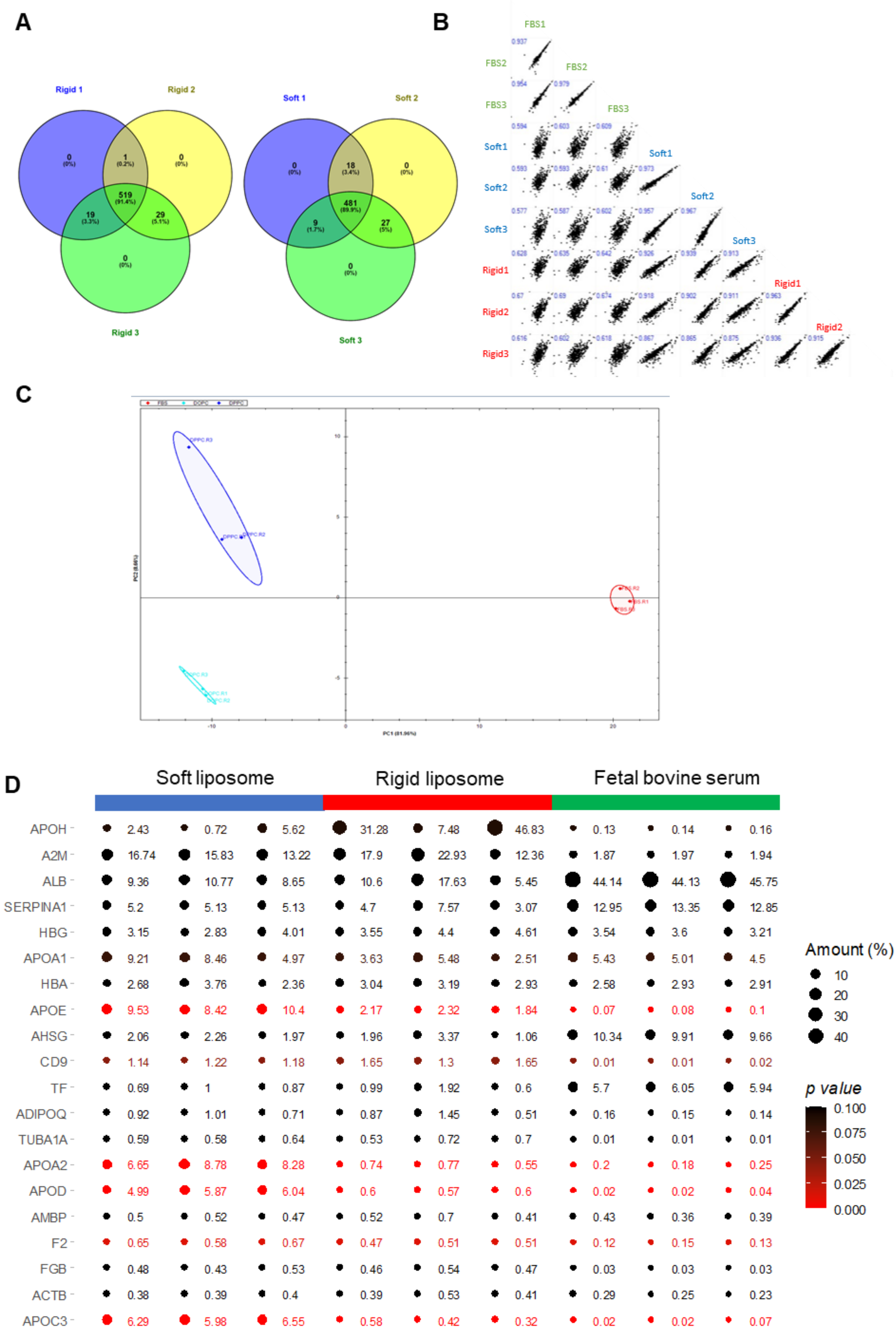
A
Rigid 1
Rigid 2
Rigid 3
0 (0%)
1 (0.2%)
519 (91.4%)
19 (3.3%)
29 (5.1%)
Soft 1
Soft 2
Soft 3
18 (3.4%)
481 (89.9%)
9 (1.7%)
27 (5%)
B
FBS1
FBS2
FBS3
Soft1
Soft2
Soft3
Rigid1
Rigid2
Rigid3
C
D
Soft liposome
Rigid liposome
Fetal bovine serum
APOH 2.43 0.72 5.62 31.28 7.48 46.83 0.13 0.14 0.16
A2M 16.74 15.83 13.22 17.9 22.93 12.36 1.87 1.97 1.94
ALB 9.36 10.77 8.65 10.6 17.63 5.45 44.14 44.13 45.75
SERPINA1 5.2 5.13 5.13 4.7 7.57 3.07 12.95 13.35 12.85
HBG 3.15 2.83 4.01 3.55 4.4 4.61 3.54 3.6 3.21
APOA1 9.21 8.46 4.97 3.63 5.48 2.51 5.43 5.01 4.5
HBA 2.68 3.76 2.36 3.04 3.19 2.93 2.58 2.93 2.91
APOE 9.53 8.42 10.4 2.17 2.32 1.84 0.07 0.08 0.1
AHSG 2.06 2.26 1.97 1.96 3.37 1.06 10.34 9.91 9.66
CD9 1.14 1.22 1.18 1.65 1.3 1.65 0.01 0.01 0.02
TF 0.69 1 0.87 0.99 1.92 0.6 5.7 6.05 5.94
ADIPOQ 0.92 1.01 0.71 0.87 1.45 0.51 0.16 0.15 0.14
TUBA1A 0.59 0.58 0.64 0.53 0.72 0.7 0.01 0.01 0.01
APOA2 6.65 8.78 8.28 0.74 0.77 0.55 0.2 0.18 0.25
APOD 4.99 5.87 6.04 0.6 0.57 0.6 0.02 0.02 0.04
AMBP 0.5 0.52 0.47 0.52 0.7 0.41 0.43 0.36 0.39
F2 0.65 0.58 0.67 0.47 0.51 0.51 0.12 0.15 0.13
FGB 0.48 0.43 0.53 0.46 0.54 0.47 0.03 0.03 0.03
ACTB 0.38 0.39 0.4 0.39 0.53 0.41 0.29 0.25 0.23
APOC3 6.29 5.98 6.55 0.58 0.42 0.32 0.02 0.02 0.07
Amount (%)
10
20
30
40
p value
0.100
0.075
0.050
0.025
0.000

Supplementary Figure 10. Analysis of the protein corona on rigid and soft liposomes. (A) Venn diagram of three replicates of the corona proteins identified on soft and rigid liposomes**. (B)** Pearson correlation plot of proteomic data. The Pearson correlation plot displays the correlation coefficients between protein expression profiles of different samples. **(C)** Principal Component Analysis of proteomic data. The PCA plot shows the clustering of protein expression profiles across different samples, with each point representing a sample and colored by soft liposome (DOPC), rigid liposome (DPPC) or FBS. Principal components 1 and 2 (PC1 and PC2) highlight the major variations in the dataset. **(D)** Analysis of the top 20 most abundant proteins in the corona formed on rigid liposomes. For each liposome, the mass spectrometry results obtained for 3 replicate corona isolations are reported, together with results for FBS in 3 replicate samples, for comparison. The relative abundance of the top 20 most abundant proteins identified in the corona formed on rigid liposomes is indicated, together with their relative abundance in the soft liposome corona and in FBS. The balloon area shows the protein abundance and the colors from black to red correspond to the p values of the one-way ANOVA analysis between soft and rigid samples ($n = 3$).

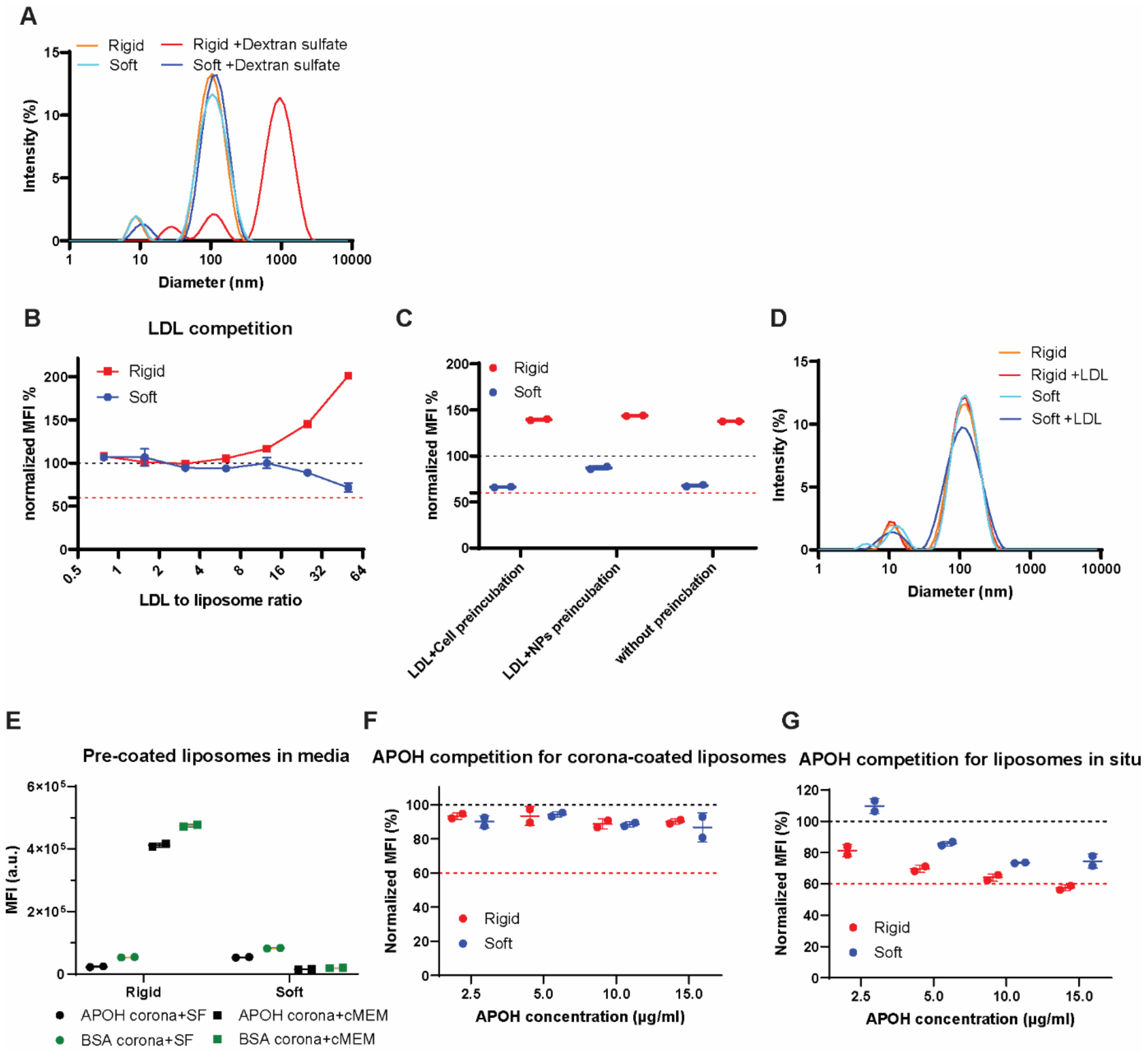

Supplementary Figure 11. Characterization of receptors involved in the uptake of rigid and soft liposomes. (A) Size distribution by intensity (diameter, nm) of 100 µg/mL liposomes in cMEM in standard conditions and in the presence of dextran sulfate (10 µg/mL) obtained by DLS. **(B)** Uptake of 100 µg/mL soft or rigid liposomes in HeLa cells (5-hour incubation) with the presence of different amount of unlabeled LDL (ratio calculated with respect to the number of liposomes). Data are shown after normalization for the uptake in cMEM in standard conditions. Symbols are the mean ± SD of median cell fluorescence intensity over 3 replicate samples. **(C)** Uptake of 100 µg/mL soft or rigid liposomes in HeLa cells (5-hour incubation) with the presence of 25x unlabeled LDL (with respect to the number of liposomes, corresponding to 18.25 µg/mL) under different design and experimental conditions. Data are shown after normalization for the uptake in cMEM in standard conditions. Symbols are the mean ± SD of the median cell fluorescence intensity over 2 replicate samples. **(D)** Size distribution by intensity (diameter, nm) of 100 µg/mL liposomes in cMEM in standard conditions and in the presence of 25x unlabeled LDL obtained by DLS. **(E)** Uptake of 100 µg/mL rigid or soft liposomes pre-coated with either BSA or APOH (6 µg per 100 µg lipids) in serum free medium or cMEM (5-hour incubation). Symbols are the mean ± SD of the median cell fluorescence intensity over 2 replicate samples. **(F-G)** Uptake of 100 µg/mL isolated corona-coated liposomes (F) or liposomes (G) in HeLa cells (5-hour incubation) with the presence of different amount of APOH. Data are shown after normalization for the uptake in cMEM in standard conditions. Symbols are the mean ± SD of the median cell fluorescence intensity over 2 replicate samples.

Supplementary Table 1. Highly enriched proteins in the rigid or the soft liposome corona

	log$_2$(LFQ intensity)					
**Protein**	**Soft_1**	**Soft_2**	**Soft_3**	**Rigid_1**	**Rigid_2**	**Rigid_3**
**Highly enriched proteins in rigid liposome corona**						
LBP	16.523331	15.738708	15.390252	19.954418	18.931015	21.344162
STX17	12.101759	NaN	12.516844	14.253721	13.694474	14.08208
A1BG	18.339703	18.276451	18.522264	19.874735	19.291468	19.506046
ACE2	12.830799	13.157904	12.622716	13.763566	13.707852	13.961637
CA2	14.136489	14.509586	14.440346	15.244297	15.289517	14.989991
ITGA5	12.546182	12.579381	12.633197	NaN	13.132296	13.132955
**Highly enriched proteins in soft liposome corona**						
APOE	24.88352	24.640251	24.871164	22.700844	22.521229	22.294624
APOD	24.655422	24.977339	24.963264	21.577707	21.252748	21.405243
APOC3	23.655361	23.196417	23.358627	20.007132	19.544794	19.397499
APOA2	23.590252	24.150015	23.01392	20.873953	20.184576	20.168909
EFCC1	23.484262	23.696848	23.756548	18.722763	17.925095	16.269407
C4BPA	22.354109	21.79336	22.94066	18.282448	17.560696	17.970695
PCYOX1	20.211531	20.534056	20.416225	17.845959	17.548109	17.37805
RUNDC3A	20.104721	18.958715	19.238012	16.917759	16.022058	17.108585
PLA2G7	20.063211	20.246063	20.102921	17.95331	17.777237	17.501757
APOC2	20.014137	19.82408	19.405972	17.409931	17.263649	15.736637
APOC4	19.900751	20.045408	19.630287	18.582855	18.488428	18.656418
HA1A	19.038597	18.478052	18.970322	17.143522	17.323462	15.756656
PCYOX1L	18.088848	18.203016	18.395805	14.958246	13.851547	13.609455
ARF1	18.079851	18.344389	18.617962	17.173649	16.753172	16.844555
RAB1B	17.776703	17.564512	17.73884	16.921421	16.700214	17.02318
RAB8B	17.580568	17.364229	17.604382	16.537888	16.363323	16.60825
RAB6B	17.397667	17.735579	17.559469	16.860909	16.40004	16.516825
BPIFB1	17.088438	16.713272	16.786636	15.574309	15.211219	14.980154
APMAP	16.960526	17.134483	17.508837	12.740691	12.846383	12.757937
SAR1B	16.667168	17.010756	17.389318	14.434414	13.124429	14.674529
DMKN	16.309198	16.009142	15.499087	13.357327	12.367277	11.169041
CCT7	16.087049	16.040937	15.570899	14.646916	14.856441	14.987195
PLA2G4A	14.253939	14.592994	14.333952	11.296223	10.029065	10.477582
ARL8B	12.895275	13.848411	13.193393	11.910039	11.484323	11.475446
PM20D1	12.808931	13.093013	13.033072	NaN	10.275266	9.4274578

Supplementary Table 2. Unique proteins in the corona formed on soft liposomes

Protein	Protein abundance (%)		
	Soft_1	Soft_2	Soft_3
YWHAH	0.0006%	0.0000%	0.0000%
ATG9A	0.0000%	0.0000%	0.0000%
RGS19	0.0000%	0.0001%	0.0002%
SRSF3	0.0000%	0.0004%	0.0005%
SLC1A1	0.0000%	0.0002%	0.0003%
SH3BGRL3	0.0000%	0.0020%	0.0023%
SAA1	0.0043%	0.0056%	0.0082%

*Proteins are present in at least two out of three soft liposome corona replicates and are not present in rigid liposome corona. The abundance values are rounded to the fourth decimal place in percentage format (0.0001%). If a value is below 0.00005%, it is displayed as 0.0000%.

Supplementary Table 3. Unique proteins in the corona formed on rigid liposomes

Protein	Protein abundance (%)		
	Rigid_1	Rigid_2	Rigid_3
GNAS	0.0053%	0.0031%	0.0017%
C5	0.0048%	0.0133%	0.0034%
AQP1	0.0028%	0.0018%	0.0068%
PSMA3	0.0019%	0.0061%	0.0010%
MARCKS	0.0017%	0.0009%	0.0011%
ARF4	0.0013%	0.0016%	0.0019%
H1-3	0.0012%	0.0000%	0.0012%
CST3	0.0009%	0.0000%	0.0006%
COL3A1	0.0008%	0.0010%	0.0010%
GSTA2	0.0007%	0.0000%	0.0008%
NME2	0.0006%	0.0006%	0.0005%
RAB13	0.0006%	0.0006%	0.0016%
RPL19	0.0005%	0.0004%	0.0013%
COL4A3	0.0004%	0.0000%	0.0006%
LAMP1	0.0003%	0.0005%	0.0008%
SLC6A9	0.0003%	0.0001%	0.0003%
SLC25A4	0.0002%	0.0001%	0.0011%
PGLYRP1	0.0002%	0.0003%	0.0017%
PARK7	0.0002%	0.0000%	0.0005%
PGM1	0.0001%	0.0001%	0.0001%
APEH	0.0001%	0.0002%	0.0001%
SERPINA6	0.0001%	0.0014%	0.0005%
NHERF1	0.0001%	0.0001%	0.0004%
TIMP3	0.0001%	0.0000%	0.0006%
PDE5A	0.0000%	0.0003%	0.0002%
HEBP1	0.0000%	0.0000%	0.0004%
KRT79	0.0000%	0.0000%	0.0004%
F7	0.0000%	0.0000%	0.0000%
PSMC2	0.0000%	0.0000%	0.0000%
GMPR2	0.0000%	0.0000%	0.0000%
SMPD1	0.0000%	0.0001%	0.0000%
PTBP1	0.0000%	0.0000%	0.0000%
MYCBP	0.0000%	0.0001%	0.0001%
HP251	0.0000%	0.0008%	0.0001%
ACE	0.0000%	0.0002%	0.0000%
SOD1	0.0000%	0.0008%	0.0003%
AMPEP	0.0000%	0.0000%	0.0000%
ENPP2	0.0000%	0.0001%	0.0001%
MYH7	0.0000%	0.0002%	0.0000%
IST1	0.0000%	0.0014%	0.0012%

*Proteins are present in at least two out of three rigid liposome corona replicates and are not present in soft liposome corona. The abundance values are rounded to the fourth decimal place in percentage format (0.0001%). If a value is below 0.00005%, it is displayed as 0.0000%.

Supplementary References:

1. Lekka, M. & Laidler, P. Applicability of AFM in cancer detection. *Nature Nanotechnology* **4**, 72-72 (2009).

Source data file for Supplementary Figure 9B

Part of the same gel (Lanes 3-5) is also shown in main Figure 5D.

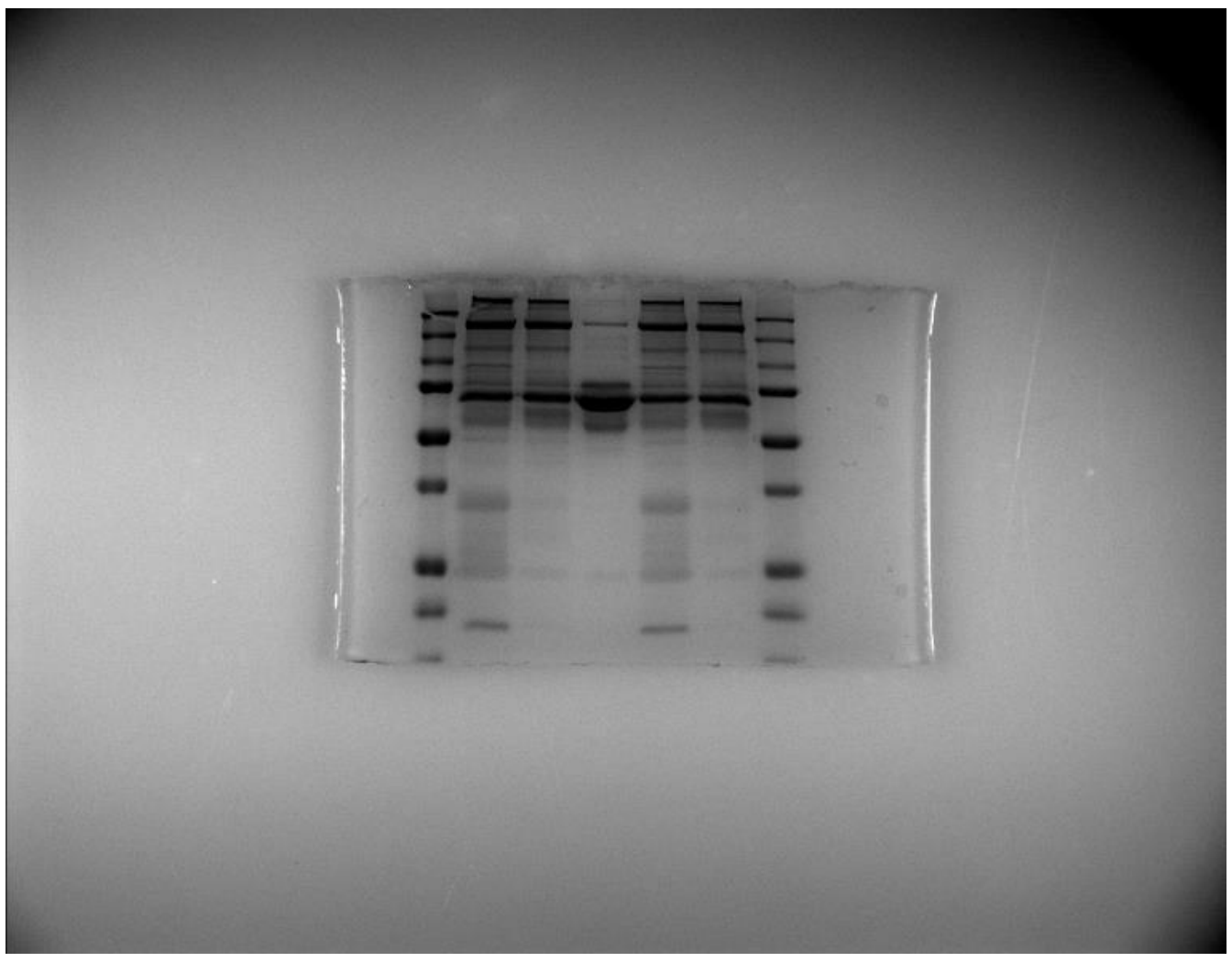

Supplement: Supplementary file 1 [file 41467_2026_77231_MOESM1_ESM.pdf]
