# Supplementary material for: Effect of liposome rigidity on corona formation and its implications for uptake by cells
Source: Nat Commun. 2026 Sep 1;17:10395. doi: 10.1038/s41467-026-77231-7 (PMC13627682; doi:10.1038/s41467-026-77231-7)
Supplement: Supplementary file 2 [file 41467_2026_77231_MOESM2_ESM.pdf]

Description of Additional Supplementary Files

Supplementary Data- Complete proteomics results. The file contains the complete mass spectrometry results and analysis of the protein corona formed on the soft and rigid liposomes in FBS, as well as of the FBS control. Protein intensities were quantified using both iBAQ and LFQ approaches, as described in the Methods. The file includes relative protein abundance (riBAQ) for each replicate sample, and protein abundance ranking for each group based on the Rank Product method as described in the Method. It also includes the most abundant proteins identified on the soft liposomes and their abundance (riBAQ) in all the different samples (also shown in Figure 6C-D and Supplementary Figure 10D), as well as the proteins uniquely identified in either the soft or the rigid liposome samples. Finally, the file also contains the raw data of the differential protein enrichment analysis shown in Figure 6B, including the log2(Fold change_ Rigid vs. Soft) and -log10(p value) used to generate the volcano plot
