# Supplementary material for: Effect of liposome rigidity on corona formation and its implications for uptake by cells
Source: Nat Commun. 2026 Sep 1;17:10395. doi: 10.1038/s41467-026-77231-7 (PMC13627682; doi:10.1038/s41467-026-77231-7)
Supplement: Supplementary file 3 [file 41467_2026_77231_MOESM4_ESM.pdf]

nature portfolio

Corresponding author(s): Anna Salvati and Wouter Roos

Last updated by author(s): Aug 3, 2026

Reporting Summary

Nature Portfolio wishes to improve the reproducibility of the work that we publish. This form provides structure for consistency and transparency in reporting. For further information on Nature Portfolio policies, see our Editorial Policies and the Editorial Policy Checklist.

Statistics

For all statistical analyses, confirm that the following items are present in the figure legend, table legend, main text, or Methods section.

n/a	Confirmed	
☐	☒	The exact sample size (*n*) for each experimental group/condition, given as a discrete number and unit of measurement
☐	☒	A statement on whether measurements were taken from distinct samples or whether the same sample was measured repeatedly
☐	☒	The statistical test(s) used AND whether they are one- or two-sided<br>*Only common tests should be described solely by name; describe more complex techniques in the Methods section.*
☒	☐	A description of all covariates tested
☐	☒	A description of any assumptions or corrections, such as tests of normality and adjustment for multiple comparisons
☐	☒	A full description of the statistical parameters including central tendency (e.g. means) or other basic estimates (e.g. regression coefficient) AND variation (e.g. standard deviation) or associated estimates of uncertainty (e.g. confidence intervals)
☐	☒	For null hypothesis testing, the test statistic (e.g. *F*, *t*, *r*) with confidence intervals, effect sizes, degrees of freedom and *P* value noted<br>*Give P values as exact values whenever suitable.*
☒	☐	For Bayesian analysis, information on the choice of priors and Markov chain Monte Carlo settings
☒	☐	For hierarchical and complex designs, identification of the appropriate level for tests and full reporting of outcomes
☐	☒	Estimates of effect sizes (e.g. Cohen's *d*, Pearson's *r*), indicating how they were calculated

Our web collection on statistics for biologists contains articles on many of the points above.

Software and code

Policy information about availability of computer code

Data collection	DLS, zeta potential, transition temperature data was acquired on ZetaSizer Nano ZS (Malvern) with Zetasizer (V7.13)<br>Nanoparticle tracking analysis data were acquired on the Zetaview X30 MONO (Particle Metrix) with ZetaView (v8.05.16)<br>Differential Scanning Calorimeter data were acquired on Q1000 DSC (TA instruments, v9.8)<br>Absorbance and fluorescence reads were acquired on BioTek Synergy H1 plate reader with BioTek Gen5 (v3.14)<br>Atomic Force Microscopy (AFM) data were acquired with JPK Nanowizard AFM (v6)<br>Flow cytometry data were acquired on the Cytoflex S (Beckman Coulter) with CytExpert (v2.4)<br>Fluorescence images were acquired on Zeiss Cell Discovery 7 with Zeiss Zen (v2.5)<br>SDS-PAGE images were acquired on ChemiDoc XRS (Bio-Rad) with GeneSys (v1.8.11.0)
Data analysis	JPK Data Processing (v6.1), Gwyddion (v2.58) were used to analyze AFM images. A script was developed in Python 3.12 to correct for the cantilever drift in the AFM images and determine the maximum height of the liposomes at each imaging force. The script is deposited at GitHub with DOI identifier 10.5281/zenodo.21605582.<br>FlowJo (v10.8.1) was used to analyze flow cytometry data.<br>Zeiss Zen (v3.10) and ImageJ Fiji (1.54p) were used to open fluorescence images, split channels and add scale bar.<br>Image J Fiji (1.54p) was used to extract intensity profiles for each lane of SDS-PAGE image.<br>Spectronaut (v16) was used to process raw MS data.<br>Perseus (v2.0.10.0), Venny (v2.1), R (v4.3.0) and R studio (v2023.03.1) were used to analyze and plot MS related images.<br>Excel (2021), OriginPro (v2021) and GraphPad Prism (v10) were used for statistical analysis.<br>GraphPad Prism (V10), Microsoft PowerPoint (2021) and Adobe Illustrator (2025) were used for figure preparation.

For manuscripts utilizing custom algorithms or software that are central to the research but not yet described in published literature, software must be made available to editors and reviewers. We strongly encourage code deposition in a community repository (e.g. GitHub). See the Nature Portfolio guidelines for submitting code & software for further information.

Data

Policy information about availability of data

All manuscripts must include a data availability statement. This statement should provide the following information, where applicable:

- Accession codes, unique identifiers, or web links for publicly available datasets
- A description of any restrictions on data availability
- For clinical datasets or third party data, please ensure that the statement adheres to our policy

Source data for all main and all supplementary fiugres are provided with the paper. The raw data are available from the corresponding author upon request. The mass spectrometry proteomics data have been deposited to the ProteomeXchange Consortium via the PRIDE partner repository with the dataset identifier PXD079018.

Research involving human participants, their data, or biological material

Policy information about studies with human participants or human data. See also policy information about sex, gender (identity/presentation), and sexual orientation and race, ethnicity and racism.

Reporting on sex and gender	This study did not involve human participants or vertebrate animals. Experiments were conducted using established human cell lines and commercially available serum products. Cell lines of both female and male origin were used, and consistent results were observed across different cell types. Donor sex information for pooled commercial serum samples was not available. Gender identity and sexual orientation were not applicable to this study.
Reporting on race, ethnicity, or other socially relevant groupings	N/A
Population characteristics	N/A
Recruitment	N/A
Ethics oversight	N/A

Note that full information on the approval of the study protocol must also be provided in the manuscript.

Field-specific reporting

Please select the one below that is the best fit for your research. If you are not sure, read the appropriate sections before making your selection.

☒ Life sciences ☐ Behavioural & social sciences ☐ Ecological, evolutionary & environmental sciences

For a reference copy of the document with all sections, see nature.com/documents/nr-reporting-summary-flat.pdf

Life sciences study design

All studies must disclose on these points even when the disclosure is negative.

Sample size	Sample sizes were determined based on established practices in the field for in vitro experiments. For each condition, experiments were performed with 2–3 technical replicates and 3–4 independent biological replicates to ensure reproducibility.
Data exclusions	No data was excluded.
Replication	All attempts at replication were successful.
Randomization	No sample randomization or allocation strategy was applied.
Blinding	Blinding was irrelevant. Knowing the tested condition would not have influenced the measurement, as these were all performed using the same settings of the instrument.

Reporting for specific materials, systems and methods

We require information from authors about some types of materials, experimental systems and methods used in many studies. Here, indicate whether each material, system or method listed is relevant to your study. If you are not sure if a list item applies to your research, read the appropriate section before selecting a response.

Materials & experimental systems

n/a	Involved in the study
☒	☐ Antibodies
☐	☒ Eukaryotic cell lines
☒	☐ Palaeontology and archaeology
☒	☐ Animals and other organisms
☒	☐ Clinical data
☒	☐ Dual use research of concern
☒	☐ Plants

Methods

n/a	Involved in the study
☒	☐ ChIP-seq
☐	☒ Flow cytometry
☒	☐ MRI-based neuroimaging

Eukaryotic cell lines

Policy information about cell lines and Sex and Gender in Research

Cell line source(s)	HeLa Cells: ATCC, CCL-2<br>A549 Cells: ATCC, CCL-185<br>THP-1 Cells: ATCC, TIB-202<br>SH-SY5Y Cells, ATCC, CRL-2266
Authentication	None of the cell lines used were authenticated by us
Mycoplasma contamination	All cell lines used were regularly tested to exclude mycoplasma contamination and no contamination was detected.
Commonly misidentified lines (See ICLAC register)	None of the cell lines used are listed in ICLAC as misidentified

Plants

Seed stocks	N/A
Novel plant genotypes	N/A
Authentication	N/A

Flow Cytometry

Plots

Confirm that:

☒ The axis labels state the marker and fluorochrome used (e.g. CD4-FITC).

☒ The axis scales are clearly visible. Include numbers along axes only for bottom left plot of group (a 'group' is an analysis of identical markers).

☒ All plots are contour plots with outliers or pseudocolor plots.

☒ A numerical value for number of cells or percentage (with statistics) is provided.

Methodology

Sample preparation	As written in the manuscript, cells were seeded in 24-well plates at a density of 50,000 cells per well, and incubated with liposomes under different conditions. Subsequently, cells were washed once with cMEM and twice with PBS to remove excess liposomes outside cells. Then cells were harvested by trypsinization using 0.05% trypsin-EDTA for 4 mins at 37°C. Following trypsinization, the cells were centrifuged at 300 rcf for 5 mins. The resulting cell pellets were resuspended in 100 µL PBS and subjected to flow cytometry measurement.
Instrument	CytoFLEX S (Beckman Coulter).
Software	Data were acquired with CytExpert (v2.4) and were anaylzed with FlowJo (v10.8.1)

Cell population abundance	Flow cytometry experiments were performed without cell sorting. Cells were analyzed as a single, unsorted population following sequential gating to exclude debris, doublets, with at least 15000 events collected.
Gating strategy	Cells were gated in FSC-H vs SSC-H, followed by single cell gate in FSC-A vs FSC-H.

☒ Tick this box to confirm that a figure exemplifying the gating strategy is provided in the Supplementary Information.
