# Supplementary material for: Effect of liposome rigidity on corona formation and its implications for uptake by cells
Source: Nat Commun. 2026 Sep 1;17:10395. doi: 10.1038/s41467-026-77231-7 (PMC13627682; doi:10.1038/s41467-026-77231-7)
Supplement: Supplementary file 4 [file 41467_2026_77231_MOESM5_ESM.pdf]

nature portfolio

Peer Review File

Effect of liposome rigidity on corona formation and its implications for uptake by cells

Corresponding Author: Dr Anna Salvati

This file contains all reviewer reports in order by version, followed by all author rebuttals in order by version.

Version 0:

Reviewer comments:

Reviewer #1

(Remarks to the Author)
The authors prepared two liposome samples with different phase transition temperature and used them as model nanoparticles to examine how nanoparticle elasticity affects cellular uptake and what roles protein corona plays in this. This is an interesting topic. The following concerns should be clarified before publication.

1, The authors used AFM to monitor the difference in mechanical strength for these two liposome samples. In the literature, most prior reports on this topic use Young's modulus as the index for elasticity. Can the AFM results be used to calculate the Young's moduli for these liposome samples, to make it easier to compare the results in this work with those in the literature?

2, A prior work (Nature Communications, volume 9, Article number: 130 (2018)) shows that mammalian cells uptake liposomes through both membrane fusion and endocytosis. In this work, the liposomes are dyed with DiI, a membrane dye, and cellular uptake is monitored by measuring fluorescence intensity. If cells uptake soft liposome through membrane fusion (and endocytosis) as suggested in that prior work, will the dilution of DiI in fused membrane affects the fluorescence emission efficiency of DiI?

Reviewer #2

(Remarks to the Author)
This study investigates the effect of liposome rigidity on protein corona formation and its implications for cellular uptake. While the work presents some interesting observations, it lacks mechanistic insight and novelty. Several key issues need to be addressed:
1) To evaluate the impact of liposome rigidity on corona formation, other variables must be controlled. Using different lipid compositions not only alters rigidity but also changes hydrophobicity, even when the head groups are identical. The authors should measure and report the hydrophobicity of the liposomes to support their assumption of identical surface properties.
2) The physicochemical properties of the two liposomes should be compared in a table, including size, polydispersity index (PDI), and zeta potential. In Figure 1C, both liposomes show shoulder peaks, please explain the reason for this observation.
3) The stiffness of the two liposomes should be quantified using Young’s modulus, as reported in previous studies. This would place the system within a defined stiffness range (e.g., kPa or mPa) and clarify how different the two formulations actually are.
4) The stability of both liposomes should be evaluated to ensure they remain intact in media and during cellular interaction.
5) The use of size-exclusion chromatography for separating liposomes may disturb the protein corona. The authors should clarify whether the isolated coronas represent soft, hard, or mixed layers, as this distinction is critical for interpretation.
6) The mechanistic studies on how the protein corona affects cellular uptake are inconclusive and should be further elaborated.

Reviewer #3

(Remarks to the Author)
In the manuscript entitled “Effect of liposome rigidity on corona formation and its implications for uptake by cells” by Ma et al. from the group of Anna Salvati the authors demonstrate that soft liposomes have a different uptake efficiency, probably other

uptake route and most interestingly a different protein corona.

I completely agree with the authors that rigidity is an understudied subject in the field of nanocarriers. I also find the approach of the group interesting and actually one of the only and undoubtedly unambiguous ones to use liposomes instead of other materials to form the nanocarriers. With lipids the rigidity/softness can be highly varied as the authors have shown as well as there is no doubt that the surface in terms of end groups as well as end group density is exactly the same for all the nanocarriers produced. On a side note I would encourage the authors to do further also use PEGylated lipids to do these investigations in a follow-up study.

First I would like to highly acknowledge that the authors do not shy off from demonstrating their journey to arrive at usable liposomes as the initial liposomes were deemed to be insufficient for further analysis due to stability/aggregation. This is not always done, but there is a highly scholarly added value in demonstrating how to develop the project in such a way that suitable nanocarriers are synthesized first in a concise way also demonstrating all the pitfalls and turns it needed to arrive there.

The investigations and characterizations (DLS, AFM, DSC) also for temperature dependence is highly appreciated by this reviewer. Also the evaluations in cell culture systems as well as the preparation of the protein corona and the proteomics appears to be thoroughly done.

I have just one (major) point that I would like the authors to address:
1. The authors hunt to define the enhanced uptake of rigid liposomes by the higher abundance of Apo H in the protein corona. I would like the authors to change the angle from which this also may be viewed: the composition of proteins attached to the liposomes is enhancing uptake in comparison to naked liposomes as shown by the authors. But: instead of looking for the “uptake enhancer” protein they may rather discuss that the found apoliproteins (especially ApoA1) may excert a stealth effect as shown in other papers (e.g. Nat Nanotechnol. 2016 Apr;11(4):372-7).

I recommend to publish this paper in Nature Communictaions after a minor revision.

Version 1:

Reviewer comments:

Reviewer #1

(Remarks to the Author)
Thanks to the authors for their time and efforts in addressing reviewer concerns. The following concerns need to be clarified before acceptance for publication.
1, The structure of the revised manuscript needs further revision. The authors used two sections, occupying four pages and a half, to show that they have constructed a model nanoparticle system which have two nanoparticles that differ only in stiffness. Maybe the authors need figure out a way to do this in a more concise yet precise way.
2, In figure 5B, the authors compared the protein composition of coronas between the soft and rigid nanoparticles. But the x-axis parameter, log2(fold change), is vague because it failed to show what baseline or control the as-indicated change is referring relative to.
3, The authors analyzed the endocytosis pathways the soft and rigid nanoparticles are internalized by the cells but put the corresponding figure into the supplementary information file. Move that figure into the maintext may be a better way, and a cartoon showing the analysis design and/or result is preferred and friendly to the audience.
4, It is still very surprising that the soft liposome, DOPC/G, here does not undergo membrane fusion with the mammalian cells in uptake assays. Normally, confocal fluorescence microscopy has resolution limit of 200 nm or higher, but the liposomes here are around 100 nm in size. It should thus be difficult to separate liposomes in the close vicinity (with a distance of <100 nm) around the cells from those on the cellular surfaces or those on the cellular surfaces from those in the cytosols.
5, In the section, Characterization of liposome bilayers and mechanical properties, the sentence, “In fact, laurdan emission peak shifts from …”, is vague and needs revision.

(Remarks on code availability)

Reviewer #2

(Remarks to the Author)
The authors have addressed some of the questions raised; however, two key issues remain unresolved.

(1) Quantitative characterisation of liposome rigidity. Referring to the particles simply as “soft” and “hard” is too qualitative and does not add substantial value to the existing literature. The mechanical differences could range from 10 kPa to 100 MPa, or alternatively from 10 kPa to 100 kPa, which would imply fundamentally different biological interpretations. Without quantitative information on the magnitude of stiffness differences, it is difficult to draw meaningful conclusions regarding the relationship between rigidity and biological behaviour.

(2) The role of surface chemistry versus particle mechanics. Surface properties are fundamentally different from bulk particle properties such as size, size distribution, or zeta potential. For example, although the study selected three specific lipids, it remains unclear whether lipids with different chemical structures but similar stiffness would generate the same protein corona profile. This is analogous to current LNP studies, where subtle variations in ionisable lipid structure can lead to substantial differences in chemistry, cellular uptake, and transfection efficiency. Similarly, in the present work, the observed differences in protein corona composition may not arise solely from stiffness effects, but could also be strongly influenced by differences in lipid chemistry and surface characteristics.

(Remarks on code availability)

Reviewer #3

(Remarks to the Author)
The authors answered all my questions in full. The manuscript can be published as is from my side of view.

(Remarks on code availability)
I have opened the github, but I guess that I am not qualified enough to judge about it as a scientific reviewer. Obviously others are much more qualified to do so.

Version 2:

Reviewer comments:

Reviewer #1

(Remarks to the Author)
My previous concerns have been resolved. Thank the authors for their time and effort.

(Remarks on code availability)

Reviewer #2

(Remarks to the Author)
The authors have addressed the questions.

(Remarks on code availability)

Open Access This Peer Review File is licensed under a Creative Commons Attribution 4.0 International License, which permits use, sharing, adaptation, distribution and reproduction in any medium or format, as long as you give appropriate

credit to the original author(s) and the source, provide a link to the Creative Commons license, and indicate if changes were made.
In cases where reviewers are anonymous, credit should be given to 'Anonymous Referee' and the source.
The images or other third party material in this Peer Review File are included in the article’s Creative Commons license, unless indicated otherwise in a credit line to the material. If material is not included in the article’s Creative Commons license and your intended use is not permitted by statutory regulation or exceeds the permitted use, you will need to obtain permission directly from the copyright holder.
To view a copy of this license, visit https://creativecommons.org/licenses/by/4.0/

Editorial policies and requests

The Reporting Summary has been compiled as required, including information on how sex and gender were considered in the study, following the Sex and Gender reporting guidelines.

The Data Availability section has been added. Scripts and proteomic raw data have been deposited in public repositories, and the required information is included in the Data Availability section. In addition the Source Data file has been prepared and is included in the submission. Plots have been made showing the individual data points. Statistical analysis has been added where relevant and details have been added in the corresponding figure captions. The gating strategy for flow cytometry analysis is now shown in a new Supplementary Figure (new Figure S6).

Finally, in light of the extensive experimental work and analysis performed to include single particle indentation measurements by AFM in order to obtain the liposome Youung's modules, a new author has been added in the revised manuscript. The author list change form has been signed by all authors and is also included in the submission.

ANSWERS TO REVIEWER COMMENTS

Reviewer #1 (Remarks to the Author):

The authors prepared two liposome samples with different phase transition temperature and used them as model nanoparticles to examine how nanoparticle elasticity affects cellular uptake and what roles protein corona plays in this. This is an interesting topic. The following concerns should be clarified before publication.

1, The authors used AFM to monitor the difference in mechanical strength for these two liposome samples. In the literature, most prior reports on this topic use Young's modulus as the index for elasticity. Can the AFM results be used to calculate the Young's moduli for these liposome samples, to make it easier to compare the results in this work with those in the literature?

Answer: Indeed in literature one find reports on how AFM has been used to make estimates of the Young's modulus of different samples. However, we prefer a direct measurement of the deformation which we showed in Figure 2 to demonstrate the difference in mechanical properties between the two samples. In fact, in order to determine the Young's module, one has to deform the particle and the resulting force-deformation curve is then fitted to a model to obtain an estimate for the modulus. It is not obvious which model is best for determining the modulus of surface deformed particles such as vesicles.[1-4] Because of this, we opted for a directly measurable parameter to show the different mechanical properties of the two samples, that is the change in deformation due to an increasing imaging force. With this direct approach we do not need to make any assumptions, nor choose between different models. This approach has been used for vesicles[5,6] and for other flexible systems like protein cages[7] and viruses.[8] Hence, we directly measured and compared the deformation between the two sets of particles. The fact that for the soft liposomes not even the deformation could be measured because most vesicles collapsed already when imaging at very low force, while instead for the rigid liposomes several vesicles resisted imaging up to 700 pN, demonstrates clearly how different the mechanical properties of the two samples are regarding the spring constant and brittleness.

Nevertheless, given that both this and Reviewer #2 suggest to add an elasticity parameter, we have now acquired additional images to extract the force indentation curves in order to make a comparison between the two different data sets. These curves are then fitted to a Hertz-like model and we obtain an elasticity parameter, which we termed the apparent modulus M_{app}. The reason that we call it "apparent modulus" is that this reflects an elasticity parameter of particles that are deformed due to mere substrate adhesion (i.e., particles that are already deformed before force indentation curves are performed). This does likely not exactly reflect the elastic parameters of the undeformed liposomes far away from a surface. As it is unclear how close or how distant the values of surface adhered liposomes are compared to liposomes far from a surface, we decided to name the resulting value of the force-indentation curve fits the apparent modulus. As we are comparing liposomes with the same head groups, mixed in the same ratio and adhered to the same type of surface, a relative comparison could be justified as a first order approximation. The new datasets (new Supplementary Figure 5) show a similar height distribution, in accordance to the previous observations in Figure 2, which allow us to confidently compare the new and previous results. In fact, from the height distribution we can already conclude that there is a stronger deformation (hence lower height) for the soft liposomes after deposition on the surface. This effect is probably driven by a lower stiffness, since the height distribution measured by DLS and zeta potential show similar results for both liposomes. The obtained values for the apparent modulus are in line with those reported for comparable liposome head group formulations,[9] even though differences in - for instance - surface preparation between the experiments complicate a direct comparison. Furthermore, the new results show a higher value of the apparent modulus for DPPC/G liposomes than for DOPC/G liposomes, in line with the relative difference that is found for the data in Figure 2.

However, another important aspect worth mentioning is the presence of lipidic bilayers deposited on the surface when imaging the soft liposomes. The presence of these lipid patches clearly visible in the AFM images indicates that many soft liposomes probably collapse already simply upon adhesion to the surface. Hence it is likely that the apparent modulus estimated from indentation measurements on the residual liposomes is actually overestimated, since it is based on those liposomes that resisted after adhering to the surface, hence the liposomes with higher stiffness within the sample.

Based on all the aspects mentioned, we consider the different deformability as shown in Figure 2 not only better as a straightforward result, but also as a better reflection of the difference in mechanical properties between the two liposomes.

As the focus of the manuscript is not on the use of AFM and associated data analysis for rigidity measurements, but on liposome uptake into cells, we prefer to keep the discussion of the AFM part relatively succinct. However, we do think it will be of added value to mention the Hertz-like fits we performed, as this could be of interest not only for the Reviewers, but also potentially for a part of the readership. Therefore, we decided to briefly mention the new data and challenges associated with the analysis. In the revised manuscript the updated and new text now reads as follows:

"Next, atomic force microscopy (AFM) was employed to compare the mechanical properties of the two liposomes. Representative topographic images are shown in Figure 2A (images at all imaging forces are shown in Supplementary Figure S4A). By imaging individual liposomes at increasing imaging forces, the decrease in height can be directly measured and provides a comparison of the difference in mechanical properties between samples. Initially, the height distribution at low imaging force (70 pN) of the DPPC/G and DOPC/G liposomes was

compared, as depicted in the violin plot in Figure 2B. The observed heights were generally lower than the diameters detected by DLS (Fig. 1D), as typically observed because of liposome deformation upon surface attachment.[10] Notably, DOPC/G liposomes showed a lower height, indicating higher deformability, as well as a greater number of lipid patches when imaged at 70 pN (Figure 2A). The presence of these patches also suggested higher deformability and consequently a higher rate of collapse upon adsorption onto the glass. At increased forces only a few liposomes remained intact. Conversely, DPPC/G liposomes demonstrated greater resilience (topographic data for all imaging forces are shown in Supplementary Figure S4A). We tracked the height change of individual DPPC/G liposomes that withstood at least three consecutive imaging forces (Figure 2C shows the normalized heights of individual particles, Figure 2D shows their average and Supplementary Figure S4B provides raw height data for individual particles) and their survival rates (Figure 2E). Even after using forces up to 200 pN, 73% of the DPPC/G liposomes remained intact. Analysis of the height vs force at 200 pN indicates a reduction of 10% of the original height measured at 70 pN. Gradually increasing the force further, up to 700 pN, resulted in total collapse of most DPPC/G liposomes, with an estimated survival population of 23%. These results clearly demonstrated the distinct mechanical properties of the two liposome formulations, with DOPC/G liposomes being much softer and brittle than DPPC/G liposomes. Additionally, an analysis of indentation curves has been performed (Supplementary Figure S5). Due to - among others - pressure built up of the adhered vesicles and fluidity of bilayers, it is challenging to obtain elastic parameters.[10,11] Supplementary Figure S5 shows a relative comparison of the elasticity, denoted as the apparent modulus M_{app}, of the two types of liposomes. This shows that the M_{app} of the DPPC/G liposomes is higher than that of the DOPC/G liposomes, consistent with results of increased imaging force experiments. Based on the AFM results, we refer hereafter to the two liposomes as "soft" and "rigid" liposomes, for simplicity."

The new results have been added in the new Supplementary Figure S5 and in the Materials and methods section we added the following text:

"Due to - among others - pressure built up of the adhered vesicles,[10] elastic parameters as deduced from force indentation curves, do not necessarily reflect elastic parameters of the undeformed particles. However, an apparent modulus, here denoted as M_{app}, can be deduced to compare relative elastic parameters between surface adhered liposomes with identical head groups and adhered to the same type of surface. Determination of the apparent modulus was performed by QI-Advanced imaging mode. Each imaging pixel represents a single nanoindentation experiment in QI mode, and QI-Advanced imaging can record those curves for each pixel. The original imaging parameters (loading rate, Z length, sampling, etc.) were kept constant. Imaging was performed at imaging forces of ~100 pN and both topography images and the force curves were processed using the JPK-DP software. The raw force curves were adjusted by subtracting the baseline, and adjustment of the point of contact and the vertical tip position. These processed curves were fitted with the Hertz/Sneddon model built in the JPK-DP, assuming a cone indenter, a half-cone angle of 15 degrees, tip radius of 10 nm and a Poisson's ratio of 0.5. OriginPro version 2021 (OriginLab Corporation, Northampton, MA, USA) was used for further data processing and statistical analysis."

2, A prior work (Nature Communications, volume 9, Article number: 130 (2018)) shows that mammalian cells uptake liposomes through both membrane fusion and endocytosis. In this work, the liposomes are dyed with Dil, a membrane dye, and cellular uptake is monitored by measuring fluorescence intensity. If cells uptake soft liposome through membrane fusion (and endocytosis) as suggested in that prior work, will the dilution of Dil in fused membrane affects the fluorescence emission efficiency of Dil?

Answer: We thank the Reviewer for referring to this work and bringing up this aspect. Some works in literature, such as the mentioned paper, report that for certain liposome formulations, membrane fusion can serve as an internalization pathway, potentially complicating the interpretation of fluorescence-based quantification, due to dye dilution within the cell membrane.

However, our data clearly demonstrate that both the soft and rigid liposomes used in our work enter cells through energy-dependent pathways rather than membrane fusion. Consequently, the potential concern regarding altered DiI fluorescence efficiency caused by fusion-induced membrane dilution doesn't apply to our study.

More in detail, energy-dependent uptake is clearly demonstrated in Figure 3E-F in the original manuscript, where a strong uptake reduction is observed upon energy depletion by incubation with sodium azide (roughly 80-90% reduction).

Furthermore, in the cited *Nature Comm.* study, membrane fusion was proposed as the predominant mechanism because liposome internalization was insensitive to different endocytosis inhibitors (e.g., filipin, chlorpromazine, dynasore). In contrast, in our work, a strong uptake reduction is observed also upon incubation with multiple transport inhibitors, including inhibitors comparable to those used in the mentioned paper together with additional ones targeting macropinocytosis and the actin cytoskeleton. The results were already included in Supporting Figure S5 in the original manuscript (now Figure S8 in the revised manuscript), and together with the sodium azide results of Figure 3E-F, further confirm that for both liposomes uptake occurs via energy-dependent endocytic mechanisms.

Energy dependent uptake is also confirmed by the confocal microscopy images of Figure 3D. In fact, if membrane fusion was involved, redistribution of DiI from punctate nanoparticle-associated signals to a diffuse cell membrane signal would be expected. Instead, confocal microscopy images of cells incubated with the two liposomes showed distinct punctate DiI signals inside cells, including in vesicles stained by Lysotracker, as expected upon energy-dependent endocytosis and distribution along the endo-lysosomal compartments (Figure 3D), with no diffuse signal along the cell membrane.

To further confirm this and exclude fusion events which would lead to transfer of the fluorescent DiI in the cell membrane, as the Reviewer mentions, we now include additional confocal microscopy images of cells incubated with liposomes for 5 hours in the presence of sodium azide to deplete cell energy. These new results are included in the new Supporting Figure S7 and show that also upon sodium azide incubation, DiI fluorescence is largely absent both on the cell membrane and inside the cells. If the liposomes would enter cells via fusion, the lipophilic dye would be expected to transfer to and accumulate on the plasma membrane even when active uptake is blocked. The absence of such membrane-located signal is a further confirmation that the liposomes used in our work do not fuse with the cellular lipid bilayer, but require active endocytosis to enter.

Collectively, both the energy depletion and transport inhibition studies, as well as the previous and newly added confocal fluorescence images confirm that endocytosis, rather than fusion, is the dominant entry mechanism for both the soft and rigid liposomes used in this work. Consistent with this, the DiI dye remains associated within the liposomes inside endocytic compartments and no transfer into the plasma membrane is observed, hence excluding potential effect of DiI dilution in the cell membrane on the measured cell fluorescence.

In order to further clarify this aspect, the following text has been included, together with reference to the mentioned Nat Comm paper (now reference 9 in the revised manuscript) and new Supplementary Figure S7:

"Confocal fluorescence microscopy (Figure 3D) confirmed liposome internalization and the overall higher uptake for the rigid liposomes, consistent with the results obtained by flow cytometry. LysoTracker staining showed that both liposomes were trafficked to the lysosomes, as usual for most nanomaterials. Other studies suggested that some liposomes may enter cells by passive fusion to the cell membrane.[12] Here instead, energy depletion by incubation with the metabolic inhibitor sodium azide (NaN_3) confirmed that for the liposomes used in this work uptake was energy-dependent rather than by passive fusion (Figure 3E-F). Consistent with this, no membrane associated staining was observed by confocal microscopy in cells incubated with the liposomes in standard conditions (Figure 3D), confirming that the fluorescent lipid DiI used for liposome tracing remained associated within the liposomes inside endocytic compartments. Similarly, no membrane-associated staining was observed in energy-depleted cells either (Supplementary Figure S7). Hence, based on these results, the lower fluorescence of cells incubated with the soft liposomes was not due to effects of DiI dilution in fused cell membranes on its fluorescence emission, but rather due to a lower uptake efficiency."

In addition, in the discussion of the results obtained with the transport inhibitors, the following text has been added:

"Overall, the observed reduction of uptake upon incubation with different transport inhibitors confirmed once more that the liposomes were internalized by cells via active mechanisms of endocytosis (as opposed to passive fusion to the cell membrane)."

Reviewer #2 (Remarks to the Author):
This study investigates the effect of liposome rigidity on protein corona formation and its implications for cellular uptake. While the work presents some interesting observations, it lacks mechanistic insight and novelty.

Several key issues need to be addressed:
1) To evaluate the impact of liposome rigidity on corona formation, other variables must be controlled. Using different lipid compositions not only alters rigidity but also changes hydrophobicity, even when the head groups are identical. The authors should measure and report the hydrophobicity of the liposomes to support their assumption of identical surface properties.

Answer: The Reviewer raises very important points that need clarification.

We fully agree with the Reviewer that varying rigidity without affecting other physico-chemical properties of the nanoparticles is very difficult. The many different nanoparticles models that

researchers have used to try to test the effect of rigidity on nanoparticle biological behaviour (distribution, targeting, cell uptake etc.) all present advantages and disadvantages in this regard. For instance, by varying polymer cross linking density mechanical properties can be varied but size and swelling behaviour are also affected.[13] Similarly, when using liposomes without and with a nanoparticle core, ensuring complete membrane coverage on every nanoparticle core is very challenging.[6]

In our model, instead, by changing lipid composition, liposomes of different mechanical properties can be obtained as indeed we demonstrate (Figure 2) while keeping comparable size, zeta potential and (as Reviewer #3 also highlights and appreciates in relation to our model) comparable "*surface in terms of end groups as well as end group density"*. We believe that keeping size, zeta potential and surface composition comparable for the two samples is a major advantage based on which we selected this specific model because these are some of the key parameters affecting nanoparticle interactions with biological fluids (corona formation) and with cells. Hence, we welcome the opportunity to clarify that we do not assume "identical surface properties" but that the model allows to obtain two liposomes of comparable size, zeta potential and surface composition (head groups etc). The manuscript has been checked to ensure consistency and clarification of this aspect.

It is also true, as the Reviewer mentions, that by changing lipid composition and the phase of the bilayer (solid ordered *vs* liquid disordered phase) the mechanical properties are varied but, as a consequence of the different bilayer fluidity, the hydration of the bilayer is affected as well. The Laurdan assay results, that were already included in our work, indeed show this. In fact, we included this assay because it is commonly used as a measurement of membrane fluidity and this is often used as an indicator of membrane rigidity.[14] At the same time, the Laurdan assay is also used to measure bilayer hydration on the liposome surface[15] (again showing how the two properties are strongly intertwined for this model). In fact, more specifically, the Laurdan assay measures the dipolar relaxation of water molecules at the glycerol level of the membrane. The higher GP value which we observed for rigid liposomes indicates a highly ordered, tightly packed gel phase that excludes water molecules from the interface. Vice versa, the lower GP of the soft liposomes reflects a loosely packed liquid phase, allowing greater water penetration. Therefore, the observed differences in Laurdan GP values confirm the different bilayer fluidity, hence different mechanical properties, but also indicate that indeed, in agreement with what the Reviewer says, the different bilayer phase affects the hydration level of the two liposomes as well.

We now expanded the discussion of the Laurdan assay already included in the original manuscript to fully clarify this and explain that in our model mechanical properties and bilayer hydration are strongly intertwined. Hence, by changing lipid composition liposomes mechanical properties are indeed varied but as a result of the different bilayer fluidity, the hydration of the bilayer is also affected.

In order to clarify this aspect, the text in manuscript has been revised as follows:

"We next compared the fluidity of the two formulations upon incorporation of laurdan in the bilayer.[16] Changes in laurdan fluorescence are commonly used to characterize membrane fluidity and this is often used as an indicator of membrane rigidity and the different mechanical properties of liposomes with bilayers in different phases.[14] In fact, laurdan emission peak shifts from 440 nm to 490 nm when it is incorporated in bilayers in the ordered phase (So, Lo) and liquid disordered phase (Ld) respectively, and this can be used to calculate a Generalized Polarization ratio (GP_{340}) (as shown in Equation 2 in the Methods). Higher GP_{340} values indicate

a more ordered (gel-like) phase, while lower GP_{340} values suggest a less ordered (liquid-crystalline) phase. Of note, as a consequence of the different bilayer phase not only the mechanical properties, but also the hydration level of the bilayer is affected and in fact the laurdan GP ratio is also used as an indicator of the bilayer hydration at the interface of the liposome surface.[15,17] Hence, in this model, nanoparticle mechanical properties and the bilayer hydration level are strongly intertwined."

Similarly, in order to take this into account when discussing the different coronas adsorbed on the two liposomes, the text has been revised as follows:

"These results clearly indicated that liposomes of different rigidity form different protein coronas upon dispersion in serum, as a consequence of the different mechanical properties, bilayer fluidity as well as bilayer hydration levels, which in this model are strongly intertwined."

Finally, in order to highlight the advantages of liposomes with lipid bilayers in different phases as a model to test the effect of nanoparticle mechanical properties on corona formation and uptake by cells, the Introduction has been modified as follows:

"To address this question, here we selected liposomes as a model to obtain nanoparticles of different mechanical properties but comparable surface composition, size and zeta potential. In fact, depending on their composition and transition temperature, lipid bilayers can form different phases: solid ordered phase (So) bilayers are rigid, while liquid disordered (Ld) phase bilayers are soft.[23,24] By selecting lipid mixtures with different lipid chain length and saturation, liposome transition temperature can be modulated.[25,26] Hence, liposomes of very different mechanical properties can be formed, while keeping their charge, surface composition and size constant."

2) The physicochemical properties of the two liposomes should be compared in a table, including size, polydispersity index (PDI), and zeta potential. In Figure 1C, both liposomes show shoulder peaks, please explain the reason for this observation.

Answer: The table containing size, PDI and zeta potential has now been added as suggested by the Reviewer as new Figure 1C.

In relation to the shoulder peaks of Figure 1C (now Figure 1D), we believe the Reviewer refers to the small peak observed for the liposomes dispersed in cell culture medium with serum (here referred to as cMEM). This additional peak at around 10nm is usual and comes from the free serum proteins in solution. Importantly, the primary liposome peak remains well defined and unchanged in position, confirming that both soft and rigid liposomes remain stable also when dispersed in biological medium with serum. This was crucial for the aim of our work and required extensive optimization of the liposome formulation, since as we show in Supporting Figures S1-3 and discuss in the manuscript, obtaining non-pegylated rigid liposomes that remain stable in serum is highly challenging.

Explanation of the presence of the additional peak in the DLS distribution for the dispersions in serum has now been added as follows:

"Importantly, DLS results showed that the two liposomes were also stable upon dispersion in medium with serum (Figure 1D), where a second small peak at around 10 nm is also visible, as usually observed from the free serum protein in solution."

3) The stiffness of the two liposomes should be quantified using Young's modulus, as reported in previous studies. This would place the system within a defined stiffness range (e.g., kPa or mPa) and clarify how different the two formulations actually are.

Answer: We now added new AFM data to estimate an elasticity parameter as suggested by this Reviewer as well as Reviewer #1. These new data are included in new Supplementary Figure S5 and confirm our previous conclusions on the differences in mechanical properties between the particles. However, we would like to stress that a direct comparison to literature values is challenging due to differences in approaches in different works (e.g., differences in surfaces used for particle adhesion for AFM measurements, lipid head groups and liposome composition, etc.). Therefore, we prefer to keep the comparison as a relative comparison of the two used particles.

Furthermore, we welcome the opportunity to clarify that we prefer a direct measurement of the deformation which we showed in Figure 2 to demonstrate the difference in mechanical properties between the two samples. In fact, in order to determine the Young's module, one has to deform the particle and the resulting force-deformation curve is then fitted to a model to obtain an estimate for the modulus. It is not obvious which model is best for determining the modulus of surface deformed particles such as vesicles.[1-4] Because of this, we opted for a directly measurable parameter to show the different mechanical properties of the two samples, that is the change in deformation due to an increasing imaging force. With this direct approach we do not need to make any assumptions, nor choose between different models. This approach has been used for vesicles[5,6] and for other flexible systems like protein cages[7] and viruses.[8] Hence, we directly measured and compared the deformation between the two sets of particles. The fact that for the soft liposomes not even the deformation could be measured because most vesicles collapsed already when imaging at very low force, while instead for the rigid liposomes several vesicles resisted imaging up to 700 pN, demonstrates clearly how different the mechanical properties of the two samples are regarding the spring constant and brittleness.

Nevertheless, given that both this and Reviewer #1 suggest to add an elasticity parameter, we have now acquired additional images to extract the force indentation curves in order to make a comparison between the two different data sets. These curves are then fitted to a Hertz-like model and we obtain an elasticity parameter, which we termed the apparent modulus M_{app}. The reason that we call it "apparent modulus" is that this reflects an elasticity parameter of particles that are deformed due to mere substrate adhesion (i.e., particles that are already deformed before force indentation curves are performed). This does likely not exactly reflect the elastic parameters of the undeformed liposomes far away from a surface. As it is unclear how close or how distant the values of surface adhered liposomes are compared to liposomes far from a surface, we decided to name the resulting value of the force-indentation curve fits the apparent modulus. As we are comparing liposomes with the same head groups, mixed in the same ratio and adhered to the same type of surface, a relative comparison could be justified as a first order approximation. The new datasets (new Supplementary Figure 5) show a similar height distribution, in accordance to the previous observations in Figure 2, which allow us to confidently compare the new and previous results. In fact, from the height distribution we can already conclude that there is a stronger deformation (hence lower height) for the soft liposomes after deposition on the surface. This effect is probably driven by a lower stiffness, since the height distribution measured by DLS and zeta potential show similar results for both liposomes. The obtained values for the apparent modulus are in line with those reported for comparable liposome head group formulations,[9] even though differences in - for instance - surface preparation between the experiments complicate a direct comparison. Furthermore, the new

results show a higher value of the apparent modulus for DPPC/G liposomes than for DOPC/G liposomes, in line with the relative difference that is found for the data in Figure 2.

However, another important aspect worth mentioning is the presence of lipidic bilayers deposited on the surface when imaging the soft liposomes. The presence of these lipid patches clearly visible in the AFM images indicates that many soft liposomes probably collapse already simply upon adhesion to the surface. Hence it is likely that the apparent modulus estimated from indentation measurements on the residual liposomes is actually overestimated, since it is based on those liposomes that resisted after adhering to the surface, hence the liposomes with higher stiffness within the sample.

Based on all the aspects mentioned, we consider the different deformability as shown in Figure 2 not only better as a straightforward result, but also as a better reflection of the difference in mechanical properties between the two liposomes.

As the focus of the manuscript is not on the use of AFM and associated data analysis for rigidity measurements, but on liposome uptake into cells, we prefer to keep the discussion of the AFM part relatively succinct. However, we do think it will be of added value to mention the Hertz-like fits we performed, as this could be of interest not only for the Reviewers, but also potentially for a part of the readership. Therefore, we decided to briefly mention the new data and challenges associated with the analysis. In the revised manuscript the updated and new text now reads as follows:

"Next, atomic force microscopy (AFM) was employed to compare the mechanical properties of the two liposomes. Representative topographic images are shown in Figure 2A (images at all imaging forces are shown in Supplementary Figure S4A). By imaging individual liposomes at increasing imaging forces, the decrease in height can be directly measured and provides a comparison of the difference in mechanical properties between samples. Initially, the height distribution at low imaging force (70 pN) of the DPPC/G and DOPC/G liposomes was compared, as depicted in the violin plot in Figure 2B. The observed heights were generally lower than the diameters detected by DLS (Fig. 1D), as typically observed because of liposome deformation upon surface attachment.[10] Notably, DOPC/G liposomes showed a lower height, indicating higher deformability, as well as a greater number of lipid patches when imaged at 70 pN (Figure 2A). The presence of these patches also suggested higher deformability and consequently a higher rate of collapse upon adsorption onto the glass. At increased forces only a few liposomes remained intact. Conversely, DPPC/G liposomes demonstrated greater resilience (topographic data for all imaging forces are shown in Supplementary Figure S4A). We tracked the height change of individual DPPC/G liposomes that withstood at least three consecutive imaging forces (Figure 2C shows the normalized heights of individual particles, Figure 2D shows their average and Supplementary Figure S4B provides raw height data for individual particles) and their survival rates (Figure 2E). Even after using forces up to 200 pN, 73% of the DPPC/G liposomes remained intact. Analysis of the height vs force at 200 pN indicates a reduction of 10% of the original height measured at 70 pN. Gradually increasing the force further, up to 700 pN, resulted in total collapse of most DPPC/G liposomes, with an estimated survival population of 23%. These results clearly demonstrated the distinct mechanical properties of the two liposome formulations, with DOPC/G liposomes being much softer and brittle than DPPC/G liposomes. Additionally, an analysis of indentation curves has been performed (Supplementary Figure S5). Due to - among others - pressure built up of the adhered vesicles and fluidity of bilayers, it is challenging to obtain elastic parameters.[10,11]

Supplementary Figure S5 shows a relative comparison of the elasticity, denoted as the apparent modulus M_{app}, of the two types of liposomes. This shows that the M_{app} of the DPPC/G liposomes is higher than that of the DOPC/G liposomes, consistent with results of increased imaging force experiments. Based on the AFM results, we refer hereafter to the two liposomes as "soft" and "rigid" liposomes, for simplicity."

In addition, in the Materials and methods section, we added the following text:

"Due to - among others - pressure built up of the adhered vesicles,[10] elastic parameters as deduced from force indentation curves, do not necessarily reflect elastic parameters of the undeformed particles. However, an apparent modulus, here denoted as M_{app}, can be deduced to compare relative elastic parameters between surface adhered liposomes with identical head groups and adhered to the same type of surface. Determination of the apparent modulus was performed by QI-Advanced imaging mode. Each imaging pixel represents a single nanoindentation experiment in QI mode, and QI-Advanced imaging can record those curves for each pixel. The original imaging parameters (loading rate, Z length, sampling, etc.) were kept constant. Imaging was performed at imaging forces of ~100 pN and both topography images and the force curves were processed using the JPK-DP software. The raw force curves were adjusted by subtracting the baseline, and adjustment of the point of contact and the vertical tip position. These processed curves were fitted with the Hertz/Sneddon model built in the JPK-DP, assuming a cone indenter, a half-cone angle of 15 degrees, tip radius of 10 nm and a Poisson's ratio of 0.5. OriginPro version 2021 (OriginLab Corporation, Northampton, MA, USA) was used for further data processing and statistical analysis."

4) The stability of both liposomes should be evaluated to ensure they remain intact in media and during cellular interaction.

Answer: We fully agree with the Reviewer that the stability of both liposomes must be carefully evaluated in biological media and during cellular interaction and indeed extensive work was focused in optimizing the formulations in order to achieve this and in particular to obtain non-pegylated rigid liposomes that remain stable upon dispersion in serum. As further detailed above in our response to comment 2 from this Reviewer, DLS data confirmed that the optimized liposomes were stable also upon dispersion in cell medium with serum (with an additional small peak at around 10 nm, as usually observed upon dispersion in medium with serum, due to the presence of free serum protein in solution).

Since ensuring that liposomes remained stable was a crucial aspect for our work, we also included in our original submission additional DLS data for liposomes dispersed in the cell culture medium with serum and kept for up to 24 h at 37 degrees and 5% CO_2, hence in the conditions applied during the cell studies (Supplementary Figure S3B). Similarly, since inhibitors may potentially affect liposome stability as well, we explicitly evaluated the stability of both liposomes in media containing all the different inhibitors tested. These additional stability data were already included in Supplementary Figure S5 in the original submission, now Figure S8 in the revised versions. Both the size distribution results obtained after 24 h incubation in the cell culture conditions and the characterization of the dispersions obtained when the transport inhibitors were added confirmed liposome stability in the conditions used for cell experiments. In order to better highlight this important aspect, the text has been revised as follows:

-in relation to the stability study after 24 h incubation in cell culture conditions:

"Additionally, both liposomes remained stable in medium with serum for up to 24 h at 37°C and 5% CO_2, hence the conditions used for cell experiments (Supplementary Figure S3B)."

-in relation to the stability study in the presence of transport inhibitors:

"Importantly, additional DLS studies were performed to confirm that the liposome dispersions in medium with serum remained stable also upon addition of the different inhibitors (Supplementary Figure S8). Agglomeration in the presence of the inhibitors could strongly affect uptake studies and impair their interpretation."

5) The use of size-exclusion chromatography for separating liposomes may disturb the protein corona. The authors should clarify whether the isolated coronas represent soft, hard, or mixed layers, as this distinction is critical for interpretation.

Answer: This is another important aspect which we are happy to clarify. During Size exclusion chromatography (SEC), weakly associated and rapidly exchanging proteins, i.e., what is referred to by definition as the soft corona, are typically lost as the sample runs through the column, while the strongly associated molecules remain adsorbed on the surface the liposomes and elute together with the liposomes, much earlier than the free serum proteins in solution. Hence SEC is a common method to isolated hard corona-coated nanoparticles. The following text has been added to clarify this, including reference to the original paper where the term "corona" was first introduced and where indeed nanoparticles with their adsorbed hard corona were isolated precisely by SEC (Ref. 12,36 in the revised manuscript):

"During SEC, nanoparticles with their strongly associated proteins (the so called hard corona) elute first through the column, while the loosely associated proteins (soft corona) and the excess free proteins elute later. Hence, SEC is a common method to separate nanoparticles with their hard corona from the excess the loosely bound soft corona proteins and the free serum proteins in solution.[18,19]"

Multiple efforts in the field are focused in developing methods to isolate nanoparticles with their hard as well as soft corona preserved in order to characterize the soft corona and determine its role in biological interactions. This is indeed an important topic in the field.

However in Figure 6F we show that the strong uptake difference observed when liposomes are dispersed in medium with serum (cMEM), is maintained when hard corona-coated liposomes are isolated by SEC and added to cells in serum free medium. This finding indicates that in our system the key determinant of the different uptake observed for the two liposome is the hard corona, which is preserved on the liposomes following SEC isolation.

This has been further explained in the revised manuscript in order to clarify interpretation. The revised text reads as follows:

"Finally, when hard corona-coated liposomes were first isolated by SEC and then added to cells in either serum free medium or reintroduced back in a medium with FBS, the uptake difference further increased to around 22 and 39-fold respectively (Figure 6F). These results suggest that while soft corona proteins (which are lost upon corona isolation by SEC) and the excess free FBS proteins in solution also affect uptake, it is primarily the nature of the adsorbed hard corona layer formed in FBS which drives the higher uptake observed for the rigid liposomes. Overall, these results clearly demonstrate that the different coronas formed on the soft and rigid

liposomes in serum lead to the observed difference in uptake by cells, because a different uptake is observed only when liposomes are added to cells in medium with serum or for corona-coated liposomes, while no such difference is present in the absence of corona for liposomes added in serum free medium to cells nor in simple BSA solutions."

6) The mechanistic studies on how the protein corona affects cellular uptake are inconclusive and should be further elaborated.

Answer:
We agree with the Reviewer on the importance of a mechanistic understanding of how the protein corona affects cellular uptake. We believe the data we presented clearly demonstrate that the observed differences in uptake between soft and rigid liposomes are a consequence of the different corona formed on the liposomes because of the different liposome mechanical properties. In fact we demonstrate, first, that liposomes of different mechanical properties adsorb different proteins in their corona (Figure 4 and 5); secondly, we demonstrate that the two liposomes are internalized by cells with different efficiency (Figure 3) and by using distinct uptake mechanisms (Supplementary Figure S8). More importantly, we demonstrate that the uptake difference is present only when liposomes are added to cells in medium with serum or for hard-corona coated liposomes added to cells in serum free medium, while for liposomes in serum free medium or in a simple BSA solution, the uptake difference is minimal (Figure 6). Hence, the mechanical properties alone do no explain the different uptake efficiency but rather the mechanical properties affect corona formation and because of this the two liposomes have very different uptake efficiency in cells.

In addition we tested whether some known nanoparticle receptor is involved in different way in the uptake of the two liposomes and we show that the LDLR receptor has a stronger involvement in the uptake of the rigid liposomes (also in Figure 6). It is known that corona proteins can mediate interactions with specific cell receptors. Hence, the different role of LDLR in uptake of the two liposomes is likely due to the observed differences in corona composition for the two liposomes (Figure 4 and 5).

We now revised the manuscript to better clarify the results of Figure 6 on the role of corona in and the different involvement of the LDLR in the uptake of the two liposomes. The revised text reads as follows:

"Overall, these results clearly demonstrate that the different coronas formed on the soft and rigid liposomes in serum lead to the observed difference in uptake by cells, because a different uptake is observed only when liposomes are added to cells in medium with serum or for corona-coated liposomes, while no such difference is present in the absence of corona for liposomes added in serum free medium to cells nor in simple BSA solutions. Furthermore, corona proteins are known to promote interaction with specific cell receptors, as well as to affect the mechanisms by which nanoparticles are internalized by cells:[20-22] consistent with this, the different coronas adsorbed on the soft and rigid liposomes are likely to explain the observed differences not only in uptake efficiency, but also in uptake mechanisms, as well as the different involvement of receptors such as LDLR in the uptake of the two liposomes. Further studies are required to fully compare the receptors involved in their uptake, as well as to identify which specific corona proteins or corona patterns may be involved."

In addition, in response to one of the comments of Reviewer #3 we have also revised the discussion of the corona proteomic results to highlight that the different uptake may be due to

increased amount of corona proteins favouring uptake, but also a lower abundance of proteins reducing uptake. Indeed a major outcome of the proteomic results is that in the soft liposomes many proteins have lower abundance than in the rigid liposomes. Some of these proteins may function as stealth proteins reducing particle uptake, hence their lower abundance in the corona of rigid liposomes may contribute to the observed higher uptake of these liposomes.

In particular the following text has been revised:

"When comparing the coronas formed on the two liposomes, our results showed that the most abundant corona proteins were comparable for the two liposomes, but their amount was higher on soft liposomes. Given the lower uptake of these liposomes, these are likely to include proteins reducing uptake by cells, such as stealth proteins or don't eat me signals.[23-25] Instead only apoH was highly abundant on the rigid liposomes, together with multiple low-abundance proteins. Hence, the higher uptake of the rigid liposomes is likely explained by the strong reduction of proteins acting as stealth proteins in comparison to the soft liposomes, as well as by the enrichment of apoH and the collective action of multiple low-abundance corona proteins that may promote uptake."

Overall, we believe the presented data and in particular the studies of Figures 6 clearly demonstrate the role of the adsorbed serum corona on the observed differences in uptake by cells. Identifying all receptors involved in the uptake of the two liposomes and the role of specific corona proteins or corona patterns in increasing or decreasing uptake is a very interesting topic object of ongoing studies but beyond the scope of this paper, whose aim instead is to demonstrate that (i) soft and rigid liposomes adsorb very different coronas and (ii) only when the serum corona is present the rigid liposomes show much higher uptake than the soft ones, hence demonstrating the role of corona in uptake and how the corona is affected by nanoparticle mechanical properties.

In order to further highlight these key outcomes, the Conclusions have been revised and now read as follows:

"In conclusion, this work demonstrates that rigid and soft liposomes adsorbed very different coronas, hence nanoparticle mechanical properties affect corona formation. Importantly, the higher uptake observed for rigid liposomes results from the observed differences in the corona forming in serum because only when the liposomes are added to cells in the presence of serum a higher uptake is observed. This is a further example illustrating how the modifications nanomedicines encounter upon administration in biological environments strongly affect their biological behavior, hence need to be taken into account when designing new formulations."

Reviewer #3 (Remarks to the Author):

In the manuscript entitled "Effect of liposome rigidity on corona formation and its implications for uptake by cells" by Ma et al. from the group of Anna Salvati the authors demonstrate that soft liposomes have a different uptake efficiency, probably other uptake route and most interestingly a different protein corona.

I completely agree with the authors that rigidity is an understudied subject in the field of nanocarriers. I also find the approach of the group interesting and actually one of the only and undoubtedly unambiguous ones to use liposomes instead of other materials to form the nanocarriers. With lipids the rigidity/softness can be highly varied as the authors have shown

as well as there is no doubt that the surface in terms of end groups as well as end group density is exactly the same for all the nanocarriers produced.

Answer: We thank the Reviewer for this positive and encouraging comments on our work and for recognizing the advantages of using liposomes to study the effect of rigidity.

On a side note I would encourage the authors to do further also use PEGylated lipids to do these investigations in a follow-up study.

Answer: We thank the Reviewer for this interesting suggestion. In the present study, we intentionally focused on non-PEGylated liposome in order to assess the effect of rigidity on corona formation and cellular uptake without the additional complexity introduced by PEG. In fact pegylation is used to strongly reduce protein binding, hence to prevent corona formation. Nevertheless, it is also now demonstrated that many pegylated nanoparticles also do have a corona (although reduced) and interestingly some studies, including the Nature Nanotech paper mentioned by this same Reviewer in a later comment, suggested that it is the nature of the adsorbed corona, and the presence of specific corona proteins like clusterin,[24,26] to explain the uptake reduction observed for pegylated nanoparticles, rather than the overall reduction of protein adsorption. Based on these findings on pegylated corona as well as based on the outcomes of our study, we fully agree that further studying whether nanoparticle mechanical properties may affect the corona of pegylated nanoparticles as well it is surely of great interest. A sentence on this, including references to the mentioned works on pegylated corona, has been added as follows:

"Of note, while here extensive work was performed to obtain stable liposomes without the addition of PEG in order not to perturb corona formation, based on the differences observed in the corona adsorbed on the soft and rigid liposomes, it would be interesting in future to establish whether nanoparticle mechanical properties may affect the corona formed on pegylated nanoparticles as well. In fact, corona formation has been reported also for pegylated nanoparticles, and it was suggested that it is the nature of the adsorbed corona and the presence of specific stealth proteins such as clusterin to explain their reduce uptake.[24] However, whether this may be further affected by nanoparticle mechanical properties remains to be elucidated."

First I would like to highly acknowledge that the authors do not shy off from demonstrating their journey to arrive at usable liposomes as the initial liposomes were deemed to be insufficient for further analysis due to stability/aggregation. This is not always done, but there is a highly scholarly added value in demonstrating how to develop the project in such a way that suitable nanocarriers are synthesized first in a concise way also demonstrating all the pitfalls and turns it needed to arrive there.

Answer: We thank the Reviewer for this highly encouraging comment and to explicitly comment on this aspect.

Indeed, achieving the optimal liposome formulation that balanced distinct rigidity with stability was not trivial and required extensive optimization. We appreciate that the Reviewer recognizes the scholarly value of reporting these data, as establishing stable systems is a crucial prerequisite for the interpretation of all subsequent biological findings which we report (the additional DLS data for the liposomes over time in biological media (Supplementary Fig S3), as well as upon addition of the transport inhibitors (Supplementary Fig S5, now Fig S8 in the revised manuscript) also belong to the same efforts).

We believe that transparently reporting the extensive efforts which we spent in optimizing the starting formulation is integral part of our findings, and we do believe that the data included on the unstable formulations and the strategy which we followed to achieve stable samples by both reducing the lipid transition temperature and adding some charge for stability could be very useful for the community, as well as essential in the context of our work and aims.

The investigations and characterizations (DLS, AFM, DSC) also for temperature dependence is highly appreciated by this reviewer. Also the evaluations in cell culture systems as well as the preparation of the protein corona and the proteomics appears to be thoroughly done.

Answer: We are grateful for the Reviewer's positive assessment of our experimental design and execution. We aimed to provide a comprehensive dataset by combining different techniques to rigorously define the physicochemical properties of the liposomes. We strongly agree that thorough characterization, both synthetic and biological identities, is the prerequisite for interpreting complex biological results. We are delighted that the Reviewer appreciates the width and depth of the characterization which we carried out.

I have just one (major) point that I would like the authors to address:1. The authors hunt to define the enhanced uptake of rigid liposomes by the higher abundance of Apo H in the protein corona. I would like the authors to change the angle from which this also may be viewed: the composition of proteins attached to the liposomes is enhancing uptake in comparison to naked liposomes as shown by the authors. But: instead of looking for the "uptake enhancer" protein they may rather discuss that the found apoliproteins (especially ApoA1) may excert a stealth effect as shown in other papers (e.g. Nat Nanotechnol. 2016 Apr;11(4):372-7).

Answer: This is a very good point and we welcome the opportunity of revising the manuscript to highlight it more clearly. The Reviewer is fully right in that the higher uptake of the rigid liposomes could be due to either corona proteins acting as "uptake enhancers" but also the absence of corona proteins acting as "uptake reducers" or don't eat me signals etc. As we comment in the abstract and in the discussion one major outcome of the corona studies is in fact the lower abundance of several apolipoproteins in the corona formed on the rigid liposomes and this could explain the higher uptake of these liposomes ("the enhanced uptake of rigid liposomes is likely driven by the lower abundance of multiple apolipoproteins, and possibly the higher abundance of apolipoprotein H and of a pattern of low-abundant corona proteins"). The example of clusterin mentioned above in relation to the corona of pegylated particles is indeed a beautiful example of corona proteins reducing uptake and was reported exactly the *Nature Nanotech* paper to which the Reviewer refers here. Similarly in past work where we correlated corona composition and uptake by cells, we found that histidine rich glycoprotein (HRG) in the corona negatively correlated with uptake and an artificial corona of HRG indeed reduce particle uptake in respect to a full serum corona.[23]

The Discussion and Conclusions have been revised to better highlight this result and the likely role of corona proteins acting as stealth proteins reducing uptake in the observed uptake differences. The revised text reads as follows:

"When comparing the coronas formed on the two liposomes, our results showed that the most abundant corona proteins were comparable for the two liposomes, but their amount was higher on soft liposomes. Given the lower uptake of these liposomes, these are likely to include proteins reducing uptake by cells, such as stealth proteins or don't eat me signals.[23-25] Instead only apoH was highly abundant on the rigid liposomes, together with multiple low-abundance proteins. Hence, the higher uptake of the rigid liposomes is likely explained by the strong

reduction of proteins acting as stealth proteins in comparison to the soft liposomes, as well as by the enrichment of apoH and the collective action of multiple low-abundance corona proteins that may promote uptake.”

I recommend to publish this paper in Nature Communications after a minor revision.

Answer: We thank the Reviewer for appreciating our work.

References:

1. Striska, L. et al. Analysis of the Hertz Contact Model for Evaluating Mechanical Properties of Polymers Using the Finite Element Method. *Polymers (Basel)* **17**(2025).
2. Dimitriadis, E.K., Horkay, F., Maresca, J., Kachar, B. & Chadwick, R.S. Determination of elastic moduli of thin layers of soft material using the atomic force microscope. *Biophys J* **82**, 2798-810 (2002).
3. Gavara, N. & Chadwick, R.S. Determination of the elastic moduli of thin samples and adherent cells using conical atomic force microscope tips. *Nat Nanotechnol* **7**, 733-6 (2012).
4. Caselli, L. et al. A plasmon-based nanoruler to probe the mechanical properties of synthetic and biogenic nanosized lipid vesicles. *Nanoscale Horizons* **6**, 543-550 (2021).
5. Alqabandi, M. et al. The ESCRT-III isoforms CHMP2A and CHMP2B display different effects on membranes upon polymerization. *BMC Biol* **19**, 66 (2021).
6. Montizaan, D. et al. Role of Curvature-Sensing Proteins in the Uptake of Nanoparticles with Different Mechanical Properties. *Small* **19**(2023).
7. Llauro, A. et al. Mechanical stability and reversible fracture of vault particles. *Biophys J* **106**, 687-95 (2014).
8. Cantero, M., Cvirkaite-Krupovic, V., Krupovic, M. & de Pablo, P.J. Mechanical tomography of an archaeal lemon-shaped virus reveals membrane-like fluidity of the capsid and liquid nucleoprotein cargo. *Proc Natl Acad Sci U S A* **120**, e2307717120 (2023).
9. Benne, N. et al. Atomic force microscopy measurements of anionic liposomes reveal the effect of liposomal rigidity on antigen-specific regulatory T cell responses. *Journal of Controlled Release* **318**, 246-255 (2020).
10. Vorselen, D., MacKintosh, F.C., Roos, W.H. & Wuite, G.J.L. Competition between Bending and Internal Pressure Governs the Mechanics of Fluid Nanovesicles. *Acs Nano* **11**, 2628-2636 (2017).
11. Vorselen, D. et al. The fluid membrane determines mechanics of erythrocyte extracellular vesicles and is softened in hereditary spherocytosis. *Nature Communications* **9**(2018).
12. Guo, P. et al. Nanoparticle elasticity directs tumor uptake. *Nature Communications* **9**, 130 (2018).
13. Ribovski, L. et al. Low nanogel stiffness favors nanogel transcytosis across an in vitro blood–brain barrier. *Nanomedicine: Nanotechnology, Biology and Medicine* **34**, 102377 (2021).
14. Takechi-Haraya, Y. et al. Atomic Force Microscopic Analysis of the Effect of Lipid Composition on Liposome Membrane Rigidity. *Langmuir* **32**, 6074-6082 (2016).
15. Parasassi, T., Krasnowska, E.K., Bagatolli, L. & Gratton, E. LAURDAN and PRODAN as polarity-sensitive fluorescent membrane probes. *Journal of Fluorescence* **8**, 365-373 (1998).
16. Parasassi, T., De Stasio, G., d'Ubaldo, A. & Gratton, E. Phase fluctuation in phospholipid membranes revealed by Laurdan fluorescence. *Biophysical Journal* **57**, 1179-1186 (1990).
17. Lester, A., Orlikowska-Rzeznik, H., Krok, E. & Piatkowski, L. Laurdan Adopts Distinct, Phase-Specific Orientations in Lipid Membranes. *J Phys Chem B* **129**, 6233-6240 (2025).
18. Cedervall, T. et al. Understanding the nanoparticle–protein corona using methods to quantify exchange rates and affinities of proteins for nanoparticles. *Proceedings of the National Academy of Sciences* **104**, 2050-2055 (2007).

19. Yang, K., Mesquita, B., Horvatovich, P. & Salvati, A. Tuning liposome composition to modulate corona formation in human serum and cellular uptake. *Acta Biomaterialia* **106**, 314-327 (2020).
20. Dilliard, S.A., Cheng, Q. & Siegwart, D.J. On the mechanism of tissue-specific mRNA delivery by selective organ targeting nanoparticles. *Proceedings of the National Academy of Sciences* **118**, e2109256118 (2021).
21. Francia, V. et al. Corona Composition Can Affect the Mechanisms Cells Use to Internalize Nanoparticles. *ACS Nano* **13**, 11107-11121 (2019).
22. Liu, K. et al. Multiomics analysis of naturally efficacious lipid nanoparticle coronas reveals high-density lipoprotein is necessary for their function. *Nature Communications* **14**, 4007 (2023).
23. Aliyandi, A., Reker-Smit, C., Bron, R., Zuhorn, I.S. & Salvati, A. Correlating Corona Composition and Cell Uptake to Identify Proteins Affecting Nanoparticle Entry into Endothelial Cells. *ACS Biomaterials Science & Engineering* **7**, 5573-5584 (2021).
24. Schottler, S. et al. Protein adsorption is required for stealth effect of poly(ethylene glycol)- and poly(phosphoester)-coated nanocarriers. *Nat Nanotechnol* **11**, 372-7 (2016).
25. Fedeli, C. et al. The functional dissection of the plasma corona of SiO(2)-NPs spots histidine rich glycoprotein as a major player able to hamper nanoparticle capture by macrophages. *Nanoscale* **7**, 17710-28 (2015).
26. Barenholz, Y. Doxil(R)--the first FDA-approved nano-drug: lessons learned. *J Control Release* **160**, 117-34 (2012).

ANSWERS TO REVIEWER COMMENTS

Reviewer #1 (Remarks to the Author):

Thanks to the authors for their time and efforts in addressing reviewer concerns. The following concerns need to be clarified before acceptance for publication.

1, The structure of the revised manuscript needs further revision. The authors used two sections, occupying four pages and a half, to show that they have constructed a model nanoparticle system which have two nanoparticles that differ only in stiffness. Maybe the authors need figure out a way to do this in a more concise yet precise way.

Answer: We thank the Reviewer for the positive comments on the revisions performed.

In light of this comment, we revised the manuscript further to try to shorten the description of liposome optimization (all results of this part were already included in the Supplementary Information, Supp. Fig. 1-3), while still describing these results in a precise way. Additionally, we merged the two sections mentioned into one. The title of the new merged section is now "Optimization and physico-chemical characterization of liposome formulations".

We believe that the characterization of the optimized liposomes constitutes a central part of the work and we hope this revised version is more concise, but still conveys the essential information for the rest of the manuscript in a precise way.

2, In figure 5B, the authors compared the protein composition of coronas between the soft and rigid nanoparticles. But the x-axis parameter, log2(fold change), is vague because it failed to show what baseline or control the as-indicated change is referring relative to.

Answer: We now changed the axis title to "log2 (Fold change_Rigid vs. Soft)" and added a panel title "Corona on rigid liposomes versus corona on soft liposomes". We noted that the caption and text were also not clear and now they have been revised to more clearly explain what the plot shows.

The revised caption of Fig 6B now reads as follows: "Volcano plot of differentially enriched ($p<0.05$) corona proteins on rigid liposomes in respect to soft liposomes. Red and blue dots represent highly enriched and depleted corona proteins identified on rigid liposomes in respect to soft liposomes (log_2 (Fold change_Rigid vs. Soft)>1 and < -1, respectively). Two significantly enriched proteins were identified on the rigid liposomes in respect to the corona on the soft liposomes and are shown in the plot with red dots and their name is indicated. Thirty one proteins were significantly depleted on the rigid liposome and are shown with blue dots (the names are included for the top 20 most depleted proteins)."

Similarly the discussion of these results has been revised as follows: "Among 528 overlapping proteins detected in both coronas, 31 proteins were significantly depleted in the corona formed on the rigid liposomes in respect to the soft liposomes, and 2 were enriched, though their combined abundance accounted for only 0.25% (Figure 6B & Supplementary Table S1). Instead, 7 of the 31 significantly depleted proteins in the rigid corona had an abundance greater than 0.5% on the soft liposomes, ranking within the top 21 abundant proteins in the soft liposome corona (Figure 6C-D, and Supplementary Table S1)."

3, The authors analyzed the endocytosis pathways the soft and rigid nanoparticles are internalized by the cells but put the corresponding figure into the supplementary information

file. Move that figure into the maintext may be a better way, and a cartoon showing the analysis design and/or result is preferred and friendly to the audience.

Answer: This is a good suggestion which we are happy to take on board. We now introduce a new main Figure 4 where we show (part of) the results obtained with endocytosis inhibitors and add a cartoon to summarize the main outcomes in an easier way.

Together with new Figure 4 and its caption, the text has been slightly revised accordingly to introduce the new cartoon summarizing the key outcomes and now reads as follows: "More specifically, the results suggested that for both liposomes uptake was actin- and dynamin-dependent, but cells likely internalize the two liposomes via different mechanisms, with macropinocytosis and (at short time) clathrin-mediated endocytosis involved in the uptake of the soft liposomes (Figure 4B provides a schematic summary of these results)."

4, It is still very surprising that the soft liposome, DOPC/G, here does not undergo membrane fusion with the mammalian cells in uptake assays. Normally, confocal fluorescence microscopy has resolution limit of 200 nm or higher, but the liposomes here are around 100 nm in size. It should thus be difficult to separate liposomes in the close vicinity (with a distance of <100 nm) around the cells from those on the cellular surfaces or those on the cellular surfaces from those in the cytosols.

Answer: We agree with the Reviewer on potential limits of optical resolution in the fluorescence microscopy images. However, in the images of Figure 3, and also in those in Supp. Fig. S7, where labelling to identify the cell membrane is included as well, overall there are not a lot of (red) liposome dots visible on the cell membrane. Furthermore, the flow cytometry results of Figure 3E show up to 90% fluorescence reduction in energy depleted cells, further confirming that uptake is energy dependent and not via fusion. Therefore absence of fusion is confirmed also with a different method that is not limited by optical resolution.

Furthermore, it is good to keep in mind that flow cytometry cannot distinguish between internalized liposomes and eventual adhering liposomes sticking on the cell membrane. Taking this into consideration, the strong drop in cell fluorescence observed by flow cytometry in energy depleted cells (Figure 3E) is also an indication that adhesion of liposomes to the cell surface, if present at all (the images of Supp. Fig S7 in energy depleted cells suggest that almost no liposomes on or close to the cell membrane are visible) is very small in comparison to active uptake. We now mentioned this in the discussion as follows: "The strong drop of cell fluorescence in energy depleted cells also indicated that liposome adhesion to the cell membrane was minimal."

5, In the section, Characterization of liposome bilayers and mechanical properties, the sentence, "In fact, laurdan emission peak shifts from …", is vague and needs revision.

Answer: The sentence has been rephrased to improve clarify and now reads as follows: "In fact, when laurdan is incorporated in bilayers in the ordered phase (So, Lo), it shows maximum emission at around 440 nm, while when inserted in bilayers in the liquid disordered phase (Ld), the maximum emission is at around 490 nm."

Reviewer #2 (Remarks to the Author):

The authors have addressed some of the questions raised; however, two key issues remain unresolved.

(1) Quantitative characterisation of liposome rigidity. Referring to the particles simply as "soft" and "hard" is too qualitative and does not add substantial value to the existing literature. The mechanical differences could range from 10 kPa to 100 MPa, or alternatively from 10 kPa to 100 kPa, which would imply fundamentally different biological interpretations. Without quantitative information on the magnitude of stiffness differences, it is difficult to draw meaningful conclusions regarding the relationship between rigidity and biological behaviour.

Answer: We agree with the Reviewer that the terms hard and soft are qualitative, and this is exactly why we introduced these terms to refer to the two liposomes only late in the manuscript, and more specifically at the end of all results on their physico-chemical characterization, only after we performed a quantitative characterization of their different mechanical properties with 2 different methods.

In fact, in the first version of the manuscript we already had a quantitative characterisation of the deformability of the two liposome (Figure 2), which showed how different the mechanical properties of the two liposomes are: while several DOPC/G liposomes already collapsed simply upon adsorption onto the glass surface and could not withstand imaging forces > 70 pN, 73% of the DPPC/G liposomes survived even when imaging at 200 pN, with only 10% reduction in height. It is only based on these quantitative results that thereafter we introduced the "soft/hard" description.

Furthermore, upon suggestion from Reviewer #1 and this Reviewer #2, in the revised version we also added a second quantitative description (the apparent Young modulus of Supp. Fig. S5 obtained after single particle indentation measurements) which also confirmed the different mechanical properties. (We discussed in detail in the previous revision the limits of this type of measurements for vesicles adsorbed on a substrate and why we consider the deformability at increasing imaging force and the direct measurement of change in height of Figure 2 a better quantification of how different the mechanical properties of the two liposomes are.)

Hence, the terms "hard" and "soft" are used only after measuring these parameters, based on two quantitative characterisations. To further stress this, we following text has been added: "Based on the two methods for the quantitative characterization of nanoparticle mechanical properties by AFM, we refer hereafter to the two liposomes as "soft" and "rigid" liposomes, for simplicity."

(2) The role of surface chemistry versus particle mechanics. Surface properties are fundamentally different from bulk particle properties such as size, size distribution, or zeta potential. For example, although the study selected three specific lipids, it remains unclear whether lipids with different chemical structures but similar stiffness would generate the same protein corona profile. This is analogous to current LNP studies, where subtle variations in ionisable lipid structure can lead to substantial differences in chemistry, cellular uptake, and transfection efficiency. Similarly, in the present work, the observed differences in protein corona composition may not arise solely from stiffness effects, but could also be strongly influenced by differences in lipid chemistry and surface characteristics.

Answer: We agree with the Reviewer that both bulk and surface properties matter. In relation to whether "lipids with different chemical structures but similar stiffness would generate the same protein corona profile", in previous studies we (and others in literature) showed that - for instance - liposome charge strongly affect both corona formation and uptake by cells.[1,2] Here liposomes were made with lipids with same alkyl chains, hence same bilayer phase and mechanical properties, but different polar heads in order to vary the lipid chemistry and specifically change the charge, and this led to very different corona profiles. This is exactly why here, instead, we chose this model with lipids with the exact same polar heads and only different alkyl chain saturation in order to maintain the surface composition in terms of identity of the polar heads and only vary the bilayer phase, hence the mechanical properties.

Nevertheless, as we discussed in the previous revision, when changing the lipid bilayer phase not only the mechanical properties, but also the hydration of the bilayer is affected, as indeed the Reviewer correctly mentioned. In fact, in the gel phase, tight lipid packing excludes water from the interface; while in the liquid disordered phase, looser packing permits greater water penetration. Hence for liposome with different bilayer phases, mechanical properties and bilayer hydration are strongly intertwined and cannot be separated, as they are both consequences of the different bilayer phase.

Overall, as we discussed in the first rebuttal, we agree with the Reviewer that for any particle model, changing mechanical properties without affecting other parameters is very hard and one has to compromise.[3-6] For instance, hydrogels of different cross linking density have been used as a model for particles of different stiffness, however changing cross linking density, changes also their size and swelling behaviour.[3] In other cases, liposomes without and with a nanoparticle core have been used, but in these cases obtaining full bilayer deposition on the core is highly challenging.[4] In this case we considered liposomes with different bilayer phase the best choice to study the effect of particle mechanical properties on corona and uptake by cells because they have comparable size, zeta potential and – importantly – same surface head composition, but very distinct mechanical properties. Reviewer #3 also commented on the advantages of using this model in this regard and indeed other studies have used liposomes with different bilayer phase and composition as a model on the effect of nanoparticle mechanical properties.[5,7]

In the previous round of revision we expanded the discussion to clarify the strong connection between mechanical properties and bilayer hydration in our model in different points in the revised manuscript. More specifically, we included discussion of this aspect when presenting the Laurdan assay results (*"Of note, as a consequence of the different bilayer phase not only the mechanical properties, but also the hydration level of the bilayer is affected and in fact the laurdan GP ratio is also used as an indicator of the bilayer hydration at the interface of the liposome surface.[30,31] Hence, in this model, nanoparticle mechanical properties and the bilayer hydration level are strongly intertwined."*), as well as later when interpreting the corona results (*"These results clearly indicated that liposomes of different rigidity form different protein coronas upon dispersion in serum, as a consequence of the different mechanical properties, bilayer fluidity as well as bilayer hydration levels, which in this model are strongly intertwined."*).

To further explain why despite this, this model was preferred to other models, in the Introduction, the following text has been added: "Modulating nanoparticle mechanical properties without affecting other physico-chemical parameters is very challenging. Different models have been developed and used for this purpose, each presenting some advantages and some limitations.[23-26] Here, liposomes composed of lipids with different lipid chain saturation

were selected as a model system because they allow to modulate nanoparticle mechanical properties without affecting some of the other key physico-chemical properties known to affect nanoparticle biological behavior….. Hence, by varying lipid chain saturation and bilayer phase, liposomes of very different mechanical properties can be formed, while keeping their size, charge and surface composition constant."

Additionally, when discussing the Laurdan assay results, the previously added text has been further revised and expanded and now reads as follows: "It is important to keep in mind that as a consequence of the different bilayer phase both the mechanical properties, as well as the hydration level of the bilayer are affected. In fact the laurdan GP ratio is also used as an indicator of the bilayer hydration at the interface of the liposome surface.[33,34] In the gel phase, tight lipid packing excludes water from the interface, while in the liquid disordered phase, looser packing permits greater water penetration. Therefore, in this model, nanoparticle mechanical properties and the bilayer hydration level are both varied, because they are strongly intertwined and cannot be separated, since they are both consequences of the different bilayer phase."

Similarly, when discussing the corona, the text has been modified again and now reads as follows: "These results clearly indicated that the two liposomes adsorbed different protein coronas upon dispersion in serum. The different coronas resulted from the observed differences in nanoparticle mechanical properties (Figure 2 and Supplementary Figure S5), as well as potential additional contributions due to differences in bilayer fluidity and bilayer hydration levels, which in this model are strongly intertwined."

We hope this additional revisions fully explain the selection made, while also openly presenting the known limits of this model.

Reviewer #3 (Remarks to the Author):

The authors answered all my questions in full. The manuscript can be published as is from my side of view.

Reviewer #3 (Remarks on code availability):

I have opened the github, but I guess that I am not qualified enough to judge about it as a scientific reviewer. Obviously others are much more qualified to do so.

Answer: We thank the Reviewer for the positive comments on the revisions performed and our manuscript.

References

1. Zhao, F., Nayyar, D., Hwang, Y.S., Bartucci, R. & Salvati, A. Effect of positive charges on liposome stability, uptake efficacy and impact on cells. *Int J Pharm* **681**, 125881 (2025).
2. Yang, K.N., Mesquita, B., Horvatovich, P. & Salvati, A. Tuning liposome composition to modulate corona formation in human serum and cellular uptake. *Acta Biomaterialia* **106**, 314-327 (2020).
3. Ribovski, L. et al. Low nanogel stiffness favors nanogel transcytosis across an in vitro blood–brain barrier. *Nanomedicine: Nanotechnology, Biology and Medicine* **34**, 102377 (2021).
4. Montizaan, D. et al. Role of Curvature-Sensing Proteins in the Uptake of Nanoparticles with Different Mechanical Properties. *Small* **19**(2023).
5. Benne, N. et al. Atomic force microscopy measurements of anionic liposomes reveal the effect of liposomal rigidity on antigen-specific regulatory T cell responses. *Journal of Controlled Release* **318**, 246-255 (2020).
6. Hui, Y. et al. Role of Nanoparticle Mechanical Properties in Cancer Drug Delivery. *ACS Nano* **13**, 7410-7424 (2019).
7. Takechi-Haraya, Y. et al. Atomic Force Microscopic Analysis of the Effect of Lipid Composition on Liposome Membrane Rigidity. *Langmuir* **32**, 6074-6082 (2016).
